# Reverse Differentiation as a Gene Filtering Tool in Genome Expression Profiling of Adipogenesis for Fat Marker Gene Selection and Their Analysis

**DOI:** 10.1371/journal.pone.0069754

**Published:** 2013-07-26

**Authors:** Mujib Ullah, Stefan Stich, Thomas Häupl, Jan Eucker, Michael Sittinger, Jochen Ringe

**Affiliations:** 1 Tissue Engineering Laboratory & Berlin-Brandenburg Center for Regenerative Therapies, Department of Rheumatology and Clinical Immunology, Charité-University Medicine Berlin, Berlin, Germany; 2 Department of Hematology and Oncology, Charité-University Medicine Berlin, Berlin, Germany; The University of Adelaide, Australia

## Abstract

**Background:**

During mesenchymal stem cell (MSC) conversion into adipocytes, the adipogenic cocktail consisting of insulin, dexamethasone, indomethacin and 3-isobutyl-1-methylxanthine not only induces adipogenic-specific but also genes for non-adipogenic processes. Therefore, not all significantly expressed genes represent adipogenic-specific marker genes. So, our aim was to filter only adipogenic-specific out of all expressed genes. We hypothesize that exclusively adipogenic-specific genes change their expression during adipogenesis, and reverse during dedifferentiation. Thus, MSC were adipogenic differentiated and dedifferentiated.

**Results:**

Adipogenesis and reverse adipogenesis was verified by Oil Red O staining and expression of *PPARG* and *FABP4*. Based on GeneChips, 991 genes were differentially expressed during adipogenesis and grouped in 4 clusters. According to bioinformatic analysis the relevance of genes with adipogenic-linked biological annotations, expression sites, molecular functions, signaling pathways and transcription factor binding sites was high in cluster 1, including all prominent adipogenic genes like *ADIPOQ*, *C/EBPA*, *LPL*, *PPARG* and *FABP4*, moderate in clusters 2–3, and negligible in cluster 4. During reversed adipogenesis, only 782 expressed genes (clusters 1–3) were reverted, including 597 genes not reported for adipogenesis before. We identified *APCDD1*, *CHI3L1*, *RARRES1* and *SEMA3G* as potential adipogenic-specific genes.

**Conclusion:**

The model system of adipogenesis linked to reverse adipogenesis allowed the filtration of 782 adipogenic-specific genes out of total 991 significantly expressed genes. Database analysis of adipogenic-specific biological annotations, transcription factors and signaling pathways further validated and valued our concept, because most of the filtered 782 genes showed affiliation to adipogenesis. Based on this approach, the selected and filtered genes would be potentially important for characterization of adipogenesis and monitoring of clinical translation for soft-tissue regeneration. Moreover, we report 4 new marker genes.

## Introduction

Human bone marrow mesenchymal stem cells, also named as multipotent mesenchymal stromal cells (MSC), are easy to isolate and culture expand, and *in vitro* and *in vivo* develop into mesenchymal tissues such as bone, cartilage and fat [1], [2]. In regenerative approaches MSC-based tissue transplants are clinically applied for the restoration of injured and diseased tissues [3]. Before going into clinical application the tissue forming process requires a proper characterization.

Adipose tissue is considered to operate the metabolic regulation, hormonal secretion, energy reservoir and temperature maintenance in a critical manner [4], [5], [6]. However, excess body fat accumulation results in obesity and associated disorders, while potential shortage leads to skin ulcers, irregular body temperature and glucose deficiency [4], [5], [6]. Apart from this, adipogenesis, the formation of adipose tissue, has an important impact on different biological aspects of aging, insulin sensitivity, lipid metabolism, stress response and inflammation [5], [6]. In regenerative medicine, engineered adipose tissue will be used for instance for the restoration of soft tissue of burn and cancer patients, and in cosmetic surgery [7].

The process of adipogenesis includes the commitment of MSC into the adipogenic lineage and their development to preadipocytes and terminally differentiated adipocytes [8], and is controlled via a series of cellular, chemical, biochemical, nutritional, hormonal and signaling sensing [4], [9], [10], [11]. Moreover, distinct genes, factors and a whole array of signal cascades play a key role in driving and regulating adipogenesis on the cellular and molecular level [12], [13]. In line, factors like fatty acid binding protein-4 (FABP4) and the transcription factor peroxisome proliferator-activated receptor-γ2 (PPARG2) have already been accepted as important markers in the context of adipogenesis [12], [13], [14]. However, many of these factors need further evaluation to clarify the events during adipogenesis along with new adipogenic markers determination.

Strikingly, many of the molecular markers for adipogenic differentiation were selected on the basis of significantly changed gene expression [12], [13], [14] after stimulation of mesenchymal stem cells (e.g. primary MSC or C3H10T1/2 cells) or preadipocytes (e.g. 3T3-L1 cells) [15] with an adipogenesis stimulating medium including insulin, dexamethasone, indomethacin and 3-isobutyl-1-methylxanthine (IBMX) [9], [16]. The cumulative action of these factors in an appropriate ratio is essential for adipogenic differentiation and adipose tissue maturation [10], [16], [17]. Here, insulin accelerates lipid storage and lipogenesis but is mostly dispensable for adipogenesis of bone marrow-derived MSC, whereas glucocorticoids or their synthetic agonists like dexamethasone, which stimulate glucocorticoid receptor pathways and activate receptors for adipocyte regulation, are essential [9], [12], [17], [18]. Indomethacin influences adipogenic differentiation and fat maturation [19]. In more detail, indomethacin not only accelerates adipogenesis by increasing *CCAAT/enhancer binding protein-β (C/EBPβ)* and *PPARG* gene expression, but also inhibits *cyclooxygenase 1* and *2* genes (*COX1* and *2*) to probably enhance adipogenesis by an inverse relationship [19]. IBMX acts as a phosphodiesterase inhibitor that stimulates cAMP response element-binding proteins and initiates and drives adipocyte formation via cyclic adenosine monophosphate dependent mechanisms [17], [20], [21], [22].

Clearly, in parallel, the adipogenic supplements take part in cellular processes other than adipogenesis. Thus, since some genes act in non-adipogenic cellular events, the number of genes with significantly changed expression during insulin, dexamethasone, indomethacin and IBMX induced adipogenesis does not reflect the actual number of adipogenic-specific markers. In other words, not all of the marker genes selected on the basis of changed gene expression after stimulation with the essential medium supplements actually represents adipogenic genes. Therefore, they are not sufficient for a proper description of adipogenesis. This emphasized the need of additional studies to narrow down the list of true adipogenic markers with new perspectives to understand adipogenesis.

We hypothesized that the expression of true adipogenic marker genes is significantly up- or downregulated during adipogenesis of MSC and reversed to the level of undifferentiated MSC during dedifferentiation of the adipogenic differentiated cells. Thus, we used the standard approach for adipogenesis of MSC (15 days) and extended them by isolation of the adipogenic differentiated cells from their secreted matrix and subsequent dedifferentiation in expansion medium (35 days). We analyzed the whole processes on the cellular and genome-wide molecular level.

Adipogenic differentiation of human MSC resulted in 991 genes with significantly changed expression values. Based on the expression values during adipogenesis and dedifferentiation, K-means clustering of these genes resulted in 4 clusters. These clusters were individually analyzed. According to its low number of lipid and fat specific biological annotations, expression sites, molecular functions, signaling pathways and transcription factor binding sites, the 209 genes of cluster 4 play a very minor role in adipogenesis. In line with our hypothesis and confirming the benefit of our approach, during dedifferentiation the expression of these genes was not reverted to the undifferentiated state. Therefore, the true marker list could be narrowed down to cluster 1–3 genes. Among those genes, we filtered *adenomatosis polyposis coli down-regulated-1* (*APCDD1*), *chitinase 3-like 1 (cartilage glycoprotein-39)* (*CHI3L1*), *retinoic acid receptor responder (tazarotene induced) 1* (*RARRES1*) and *sema domain*, *immunoglobulin domain (Ig)*, *short basic domain*, *secreted*, *(semaphorin) 3G* (*SEMA3G*) as possible new adipogenic marker genes, which were not mentioned in the context of adipogenesis so far.

## Materials and Methods

### Ethics statement

All subjects participating in this study provided written informed consent to participate in this study, which was approved by the local ethical committee of the Charité-University Medicine Berlin.

### Human MSC isolation, expansion and adipogenic differentiation

Human MSC were isolated from iliac crest bone marrow aspirates of three informed and consenting patients (64, 78 and 78 years old) who were examined to exclude hematopoietic neoplasms and were histologically diagnosed as normal. As already described [23], aspirates were mixed with culture medium consisting of DMEM (Biochrom, Berlin, Germany), 10% fetal bovine serum (FBS; Thermo Scientific Hyclone, Logan, USA), 2 ng/ml basic fibroblast growth factor (PeproTech, Hamburg, Germany), 4 mM L-glutamine, 100 U/ml penicillin and 100 µg/ml streptomycin (all Biochrom), and were seeded at a density of 2×10^5^ nucleated cells per cm^2^. After 48 h cultivation in monolayer, non-adherent cells were washed out by the first media exchange. During cell expansion up to passage 4 (P4), culture medium was changed three times weekly and after reaching 90% confluence, cells were detached by the addition of 0.05% trypsin/1 mM EDTA (both Biochrom), and re-plated at a density of 5×10^3^ cells/cm^2^.

For adipogenic differentiation, 2×10^4^ MSC (n = 3 patients, P4) were incubated for 3 days in induction medium followed by 2 days in maintenance medium in 3 consecutive cycles. The maintenance medium consisted of DMEM (4.5 g/l glucose; Biochrom) containing 10% FBS, 10 µg/ml insulin (Novo Nordisk, Mainz, Germany), 100 U/ml penicillin and 100 µg/ml streptomycin. The induction medium consisted of maintenance medium supplemented with 1 µM dexamethasone, 0.2 mM indomethacin and 0.5 mM IBMX (all Sigma-Aldrich, Taufkirchen, Germany). For controls only the maintenance medium was used.

### Isolation and dedifferentiation of adipogenic differentiated cells

For dedifferentiation or reverse adipogenesis, the adipogenic differentiated cells (n = 3 patients) were isolated from their secreted extracellular matrix by incubation with 0.05 trypsin/1 mM EDTA in phosphate-buffered saline (PBS; Biochrom) for 8 min at 37°C. Then, 5×10^3^cells/cm^2^ were seeded and culture expanded (dedifferentiated) for 35 days or 4 passages in monolayer culture in MSC culture expansion medium as described above.

### Histological evaluation of adipogenic differentiated and dedifferentiated cells

To assess the content of lipid vacuoles in adipogenic differentiated and dedifferentiated cells, Oil Red O staining was performed. Briefly, the cell monolayer was washed with PBS after removing the medium and then stained with Oil Red O (Roth, Karlsruhe, Germany) for 30 min at room temperature in the dark. Red lipid droplets were evaluated using a light microscope.

### RNA extraction from cell cultures

To ensure high quality of RNA, cell cultures were homogenized in TriReagent (Sigma-Aldrich). Subsequently, for protein separation from nucleic acid, 1-bromo-3-chloropropane was added (133 µl/ml TriReagent), incubated for 15 min, and centrifuged. Then, the upper phase being free of proteins was transferred to the same volume of 70% ethanol. The RNA was further purified applying Qiagen's RNeasy Mini Kit (Qiagen, Hilden, Germany) including DNAse digestion. Finally, total RNA was eluted with RNase-free water and their quality and quantity was determined using the Bioanalyzer (Agilent Technologies, Boeblingen, Germany) and NanoDrop (NanoDrop, Wilmington, USA). The total RNA was used for quantitative real-time RT-PCR analysis as well as for microarray gene expression profiling.

### Quantitative RT-PCR analysis

First, cDNA was synthesized from the extracted total RNA (2.5 µg) with the iScript cDNA reverse transcription synthesis kit (BioRad, Munich, Germany). Then, the expression of genes of interest was analyzed using TaqMan quantitative real-time RT-PCR (qRT-PCR). The gene expression assays for TaqMan probes and primer sets (Applied Biosystems, Darmstadt, Germany) were performed in triplicates in optical plates on a Mastercycler® ep realplex^2^ S system (Eppendorf, Hamburg, Germany). Quantitative gene expression was analyzed for *APCDD1* (assay ID: Hs00537787_m1), *CHI3L1* (Hs01072228_m1), *FABP4* (Hs01086177_m1), *PPARG* (Hs01115513_m1), *RARRES1* (Hs00161204_m1), *SEMA3G* (Hs00220101_m1) and *glyceraldehyde-3-phosphate dehydrogenase* (*GAPDH*; Hs99999905_m1). The expression of genes of interest was normalized to the endogenous *GAPDH* expression level and relative quantification values were calculated in percent of *GAPDH* via using the 2^−ΔΔ*Ct*^ formula [24].

### Genome-wide gene expression profiling

For genome-wide expression profiling, Affymetrix HG-U133 plus 2 GeneChips (Affymetrix, Santa Clara, USA) were selected and analysis was performed according to Affymetrix recommendations. Briefly, 1 µg total RNA were used to synthesize biotin-labelled cRNA and 15 µg of fragmented cRNA were hybridized to GeneChips for 16 h at 45°C. Washing, staining and scanning of the GeneChips was performed using Affymetrix equipment, expression raw data were processed with Affymetrix GeneChip Operating Software (GCOS) 1.4 for signal calculation, and pairwise chip comparison was performed with GCOS 1.4 software after generating DAT, CEL and EXP files.

Expression profiling was performed for total 12 samples (n = 3 donors), subdivided in 4 time points: 3× (undifferentiated MSC), 3× (adipogenic differentiated cells at day 15), 3× (early state of dedifferentiated cells at day 7) and 3× (late state of dedifferentiated cells at day 35). Parts of the gene expression profiling raw data derived from these cultures have already been used and processed in a study on MSC transdifferentiation in a totally different way and as a result, cell cycle genes that regulate MSC differentiation, dedifferentiation and transdifferentiation were reported [23].

The microarray data sets have been submitted to Gene Expression Omnibus (GEO) database and are accessible via the GEO ID: GSE36923.

### Data normalization, selection criteria and analysis strategy

To eliminate experimental or data acquisition variations, gene expression raw data were normalized, log transformed and statistically analyzed with GCOS 1.4 software.

As introduced, first we were interested in genes whose expression was significantly up- or downregulated during the course of adipogenic differentiation. Thus, in the first step, for comparative gene expression analysis each of the 3 GeneChips on day 15 (differentiated state) was compared with each of the 3 GeneChips on day 0 (undifferentiated state). Genes were selected as differentially expressed on the basis of specific change call and fold change (FC) criteria. The change call limit was 100% (9 of 9 possible significant change calls for 3× day 15 versus 3× day 0), and the FC limit >2 or <−2 for the mean FC of nine comparisons. This way, genes that were differentially expressed during adipogenesis could be selected.

Next, we were particularly interested in those of the genes selected in step one, whose expression value during dedifferentiation reverted to the expression value before adipogenic induction (undifferentiated MSC). Therefore, in the second step, we compared the day 0 gene expression values of genes identified in step number one with the corresponding values on day 7 (early dedifferentiated state) and day 35 (late dedifferentiated state).

### Classification of genes into clusters and association with biological parameters

In order to classify the selected genes for further evaluation into suitable groups, K-means clustering was performed. Applying the Genesis Expression Similarity Investigation Suite software package 1.7.2 [25], initially Figure of Merit (FOM) analysis was carried out to determine the appropriate number of clusters [26]. Then, based on this information, the K-means clustering tool of the Genesis software was carried out and the selected genes were classified in distinct clusters based on their expression pattern.

The gene list of each individual cluster was uploaded in the Database for Annotation, Visualization and Integrated Discovery (DAVID) 6.7 and analyzed according to the default set of statistical parameters [27], [28]. For each cluster we were interested in the gene ontologies, cellular compartmentalization, molecular functions, sites of expression, functional classification and determination of transcription factor binding sites (TFBS). In addition, the gene lists were screened for genes described in the context of adipogenesis relevant signaling pathways. DAVID and the Kyoto Encyclopedia of Genes and Genomes (KEGG) were used [29]. Each relevant gene was evaluated for its expression value along with its statistical relevance during differentiation and dedifferentiation.

### Statistical analysis

Statistical analysis was performed with SigmaStat 3.5 (Systat Software, USA), while GraphPad Prism4 (GraphPad Software, USA) was applied for drawing graphs. For two group comparisons simple student t-test was used, and for three or more group comparisons one-way ANOVA. Data sets are reported as means ± SEM and asterisks were assigned to the p-values in the order P***<0.001, P**<0.01 and P*<0.05 for statistical significance. The abbreviation ns was used for statistically non-significant data sets.

## Results

### Adipogenic differentiation of human MSC

Human mesenchymal stem cells were isolated from iliac crest bone marrow aspirates and culture expanded up to P4. Based on their morphology, surface marker profile and potential to differentiate into fat, bone and cartilage, the MSC character of the cultures has already been shown elsewhere [23].

**Figure 1:**
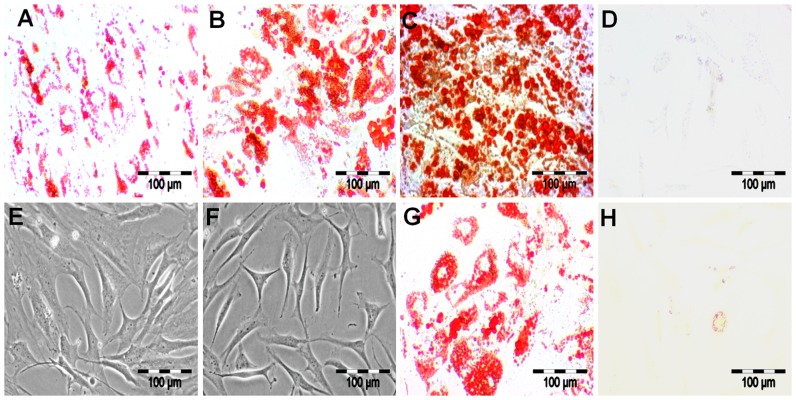
MSC isolation, adipogenic differentiation and dedifferentiation. MSC were induced to adipogenic differentiation for 15 days. (**A**) Oil Red O staining showed the formation of lipid droplets on day 5, (**B**) which increased in size and number, as shown on day 10, and (**C**) reached a peak value on day 15 of adipogenic differentiation. (**D**) Control samples showed no lipid formation even after day 15 of adipogenesis. Oil Red O staining during the conversion of adipogenic differentiated cells into dedifferentiated cells showed (**G**) an intermediate conversion after day 7 and (**H**) complete conversion after day 35 of reverse adipogenesis. Morphology of (**F**) dedifferentiated cells and (**E**) undifferentiated MSC are shown by phase contrast microscopy. Bar: 100 µm.

**Figure 2:**
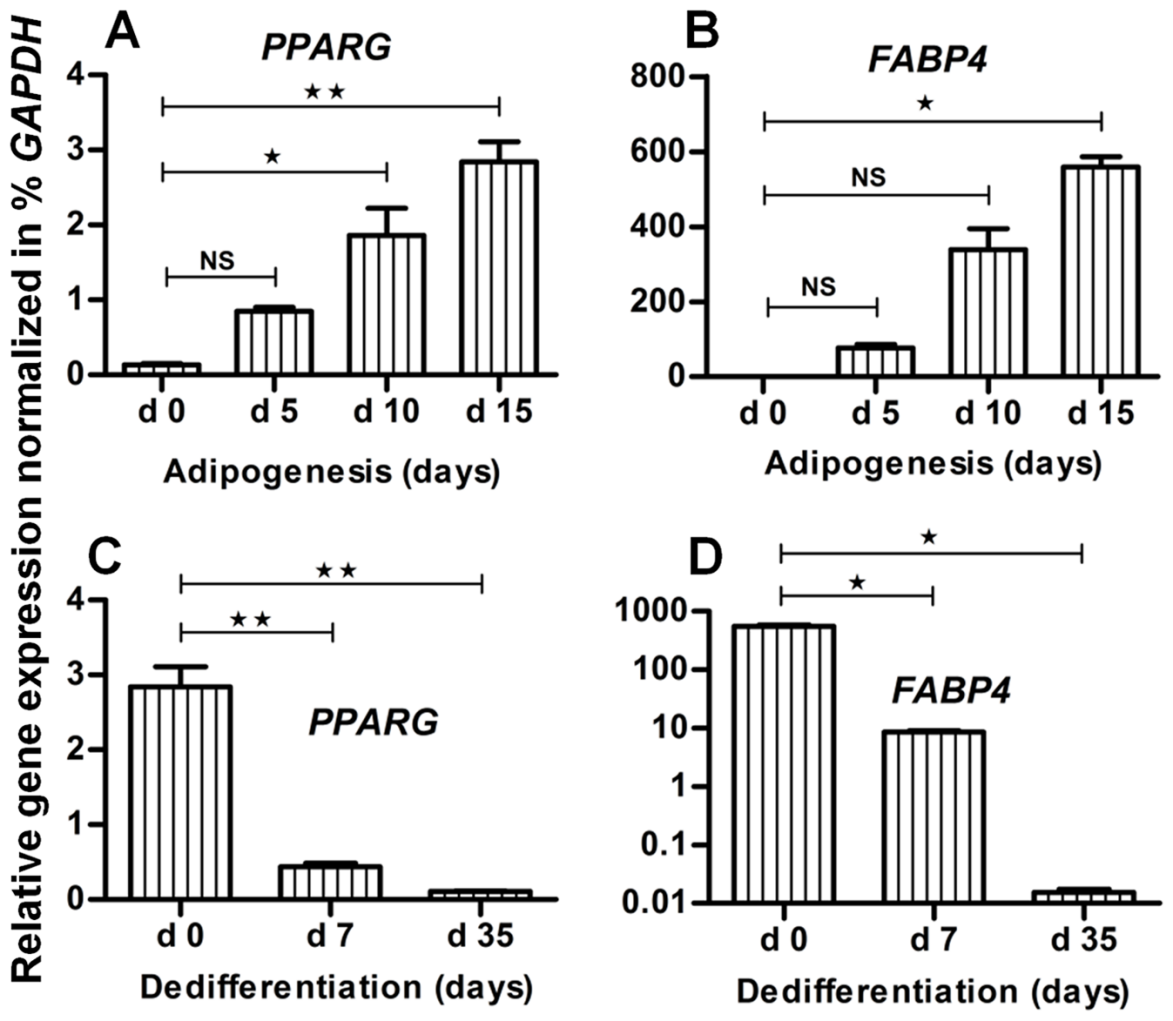
Gene expression profile of fat specific marker genes to assess adipogenesis and reverse adipogenesis. Gene expression analysis was performed using qRT-PCR and the resulted expression data were normalized to *GAPDH* for stepwise assessment of adipogenesis and reverse adipogenesis. Gene expression of adipogenic-specific marker genes (**A**) *PPARG* and (**B**) *FABP4* is given for different stages of adipogenic differentiation i.e. at day 5, day 10 and day 15. Similarly, the gene expression of (**C**) *PPARG* and (**D**) *FABP4* is given for different stages of reverse adipogenesis (dedifferentiation). Error bars, Means ± S.E.M (n = 3); *P<0.05; **P<0.01; ***P<0.001, NS, not significant (student t test performed for statistical analysis).

The MSC were induced towards the adipogenic lineage. On day 5, we observed the formation of Oil Red O stained lipid rich vacuoles (Figure 1A). The quantity and diameter of these vacuoles was continuously increased from day 10 (Figure 1B) to day 15 (Figure 1C). At this stage, we also observed a secretion of lipid droplets into the medium (data not shown). Not stimulated control cultures showed no formation of lipid droplets (Figure 1D). Adipogenesis was also confirmed on the molecular level applying qRT-PCR. Here, the expression of the adipogenic marker genes *PPARG* (Figure 2A) and *FABP4* (Figure 2B) in relation to the expression of the housekeeping gene *GAPDH* was continuously increased during adipogenic culture.

### Dedifferentiation of adipogenic differentiated cells

For dedifferentiation, the adipogenic differentiated cells (day 15) were isolated from their secreted fat matrix and cultured for 35 days in culture medium. As shown in detail elsewhere, the adipogenic differentiated cells were converted to dedifferentiated cells with fibroblast-like morphology, no lipid rich vacuoles and the capacity to develop into fat, bone and cartilage [23].

Briefly, as the dedifferentiated cells were derived from adipogenic differentiated cells, dedifferentiation was assessed on the basis of Oil Red O staining. After 7 days, we observed a slightly decreased size and number of lipid rich vacuoles (Figure 1G; early dedifferentiated state). After 5 weeks of dedifferentiation, we found a negative Oil Red O staining (Figure 1H). During dedifferentiation, the adipogenic differentiated cells were switched from bloated to fibroblast-like cell morphology and showed a phenotype (Figure 1F) comparable to undifferentiated MSC (Figure 1E). Likewise adipogenesis, also dedifferentiation was verified on the mRNA level. The expression of *PPARG* (Figure 2C) and *FABP4* (Figure 2D) in relation to *GAPDH* was continuously decreased during dedifferentiation. Taken together, all the results confirmed an advanced state of dedifferentiation.

### Expression profiling of undifferentiated MSC, differentiated and dedifferentiated cells

First, to identify the expression profile of undifferentiated MSC, adipogenic differentiated and dedifferentiated cells, we performed a genome-wide GeneChip analysis. The raw data are available in the GEO database (ID: GSE36923). Then, the gene profiles of adipogenic differentiated cells at day 15 (n = 3 patients) were compared to undifferentiated MSC (n = 3 patients). Genes were defined as differentially expressed when the mean FC of 9 comparisons was >2 or <−2 and the change call was 100% (9 of 9 comparisons). This resulted in 1459 probe-sets, which were differentially expressed during adipogenesis. Then, the probe-set list was filtered, sorted and double entries for the same genes as well as entries without any gene title or symbol were removed. This resulted in a list of 991 genes representing possible genes of interest in the context of adipogenesis. Among them, 307 were up- and 684 were downregulated (Suppl. Table S1). Finally, the expression values of these genes were compared with the values after 7 days (n = 3 patients) and 35 days (n = 3 patients) in dedifferentiation culture.

### Cluster analysis of the selected genes revealed 4 main groups

**Figure 3:**
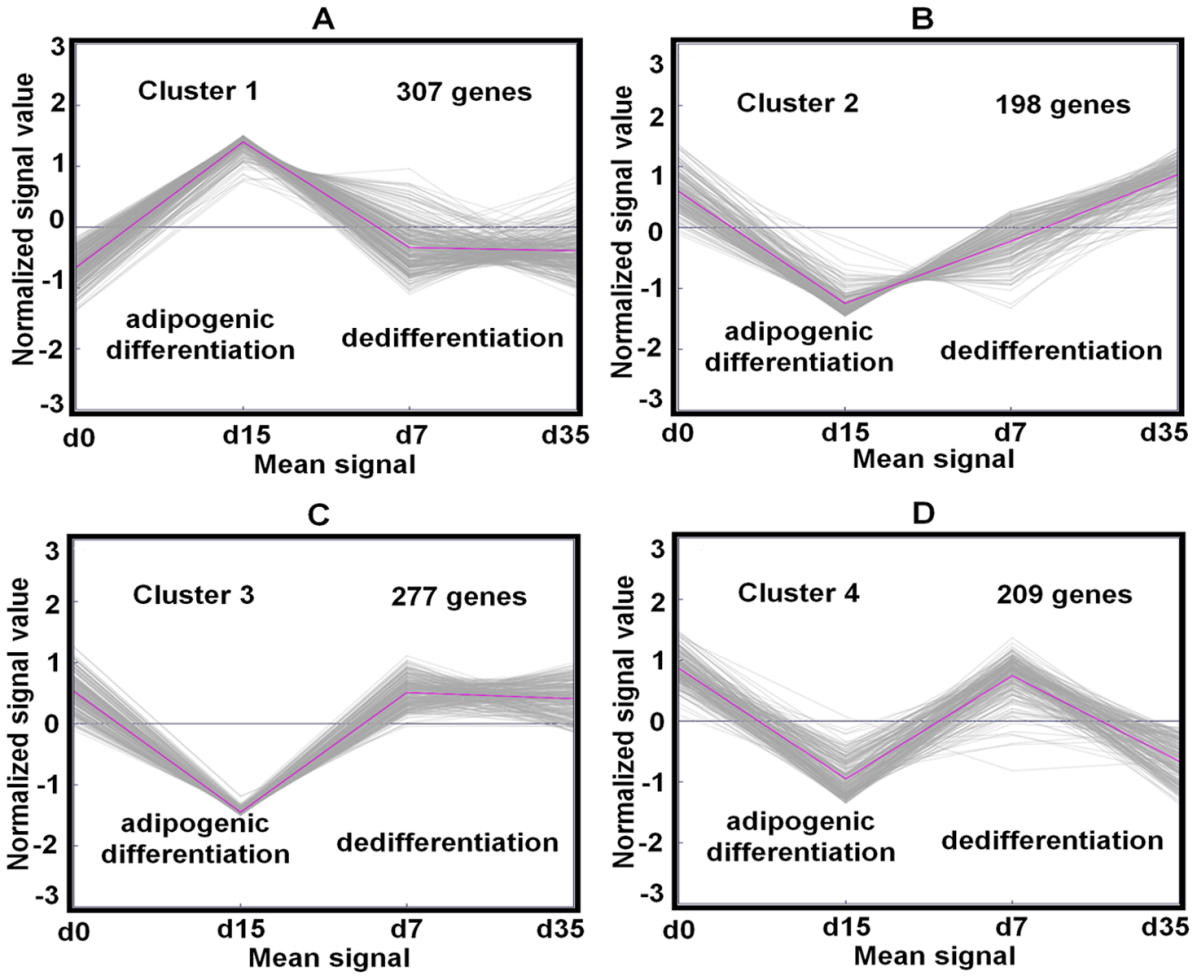
K-means clustering of differentially expressed genes. K-means clustering of 991 genes differentially expressed in adipogenesis resulted in 4 distinct gene groups. (**A–D**) Each group is subdivided into 4 time points, i.e. undifferentiated MSC (day 0), adipogenic differentiated cells (day 15), dedifferentiated cells at early time point (day 7) and dedifferentiated cells at late time point (day 35). (**A**) Genes in cluster 1 are upregulated after 15 days of adipogenic differentiation and downregulated to the level on day 0 during reverse adipogenesis. (**B–D**) Cluster 2–4 genes are downregulated after 15 days of adipogenesis and upregulated to the level on day 0 (**B**) during 35 days or (**C,D**) 7 days of dedifferentiation. Cluster 3 genes maintained this level, cluster 4 genes not. (**A–D**) The gray lines represent the individual gene expression and pink line represents the mean gene expression with respect to each time point in each group. See Suppl. Table S1, for detailed gene lists of each cluster.

In order to break the 991 selected genes into more suitable groups for further evaluation, K-means clustering was performed. The appropriate number of clusters was calculated with the Genesis analytical tool of FOM (Suppl. Figure S3), and the genes were grouped into 4 clusters (Figure 3, Suppl. Table S1). Here, the gray color lines represent the individual gene expression kinetics while the pink color line shows the cumulative average of the specific clusters expression kinetics. The relative temporal gene expression is given on the y-axis while the 4 different time points (day 0: undifferentiated state, day 15: differentiated state, day 7 and 35: early and late dedifferentiated state) are given on the x-axis.

Cluster 1 (Figure 3A) represents a group of 307 genes, such as *PPARG*, *FABP4* and most other prominent markers, whose expression was upregulated during adipogenesis, downregulated in dedifferentiated cells on day 7, became comparable to the value of undifferentiated MSC, and then remained constant until day 35. Therefore, the expression values of cluster 1 genes in dedifferentiated cells reverted to a value nearly equal to those in undifferentiated MSC. The expression of the 198 cluster 2 genes (Figure 3B) like *insulin-like growth factor binding protein-3* (*IGFBP3*) was downregulated during adipogenesis and continuously upregulated during dedifferentiation. On day 35, the expression values of cluster 2 genes in dedifferentiated cells reverted to a value nearly equal to those in undifferentiated MSC. Also the expression of the 277 genes of cluster 3 (Figure 3C), such as *IGFBP*6, was downregulated during adipogenesis but upregulated in dedifferentiated cells on day 7 to the value in undifferentiated MSC, and then remained constant until day 35. Interestingly, cluster 4 (Figure 3D) represents a group of 209 genes, which until day 7 showed the expression time course of cluster 3 genes, but then again were downregulated to the expression value in adipogenic differentiated cells.

In conclusion, the expression of cluster 1–4 genes was significantly up- or downregulated during adipogenesis, but during dedifferentiation only the expression of cluster 1–3 genes was reverted to a value similar to those in undifferentiated MSC; the cells from which they are derived from. Therefore, according to our hypothesis that true markers are differentially expressed during differentiation and that their expression values are reverted to the level of undifferentiated MSC during dedifferentiation, among the 991 genes, first and foremost the 782 (cluster 1–3) genes are relevant. This was the initial result of our approach, extending the standard approach for adipogenesis by isolating the differentiated cells from their secreted matrix and subsequent dedifferentiation. To proof the hypothesis and to get new insights in adipogenesis, in the next steps cluster 1–4 genes were analyzed in the context of biology.

### Association of cluster genes with biological parameters

To retrieve more information about the 4 clusters, the gene list of each individual cluster was uploaded in the online database DAVID. Here, first a list of all biological and functional annotations was created for each cluster. These lists were then sorted and filtered on the basis of the statistical relevant enrichment scores (first priority) and the relevance of the entries in context of adipogenesis or adipose tissue (second priority; Table 1). This way, we divided the biological information of each cluster into the 6 categories: biological annotation, cellular compartmentalization, molecular function, signaling pathways, functional classification, and site of expression. As shown in Table 1, the cumulative view of all these parameters indicated that cluster 1 represents a group of genes that has stronger affiliation to adipogenesis than the other clusters. In more detail, for cluster 1 genes, we found the most relevant entries for lipid and fat specific biological annotations, molecular functions, signaling pathways and the other biological events. Following this argumentation, clusters 2 and 3 representing genes have a minor influence on adipogenesis. The entries for cluster 4 have no or minute relation to adipogenesis, thus, indicating a very minor role in adipogenesis.

**Table 1:** Evaluation of different biological parameters for each cluster.

Cluster 1					
Gene ontologies	Cellular compartmentalization	Molecular function	KEGG signaling pathways	Functional categories	Expression site
GO:0008610∼lipid biosynthetic process (24)	GO:0005739∼mitochondrion (59)	GO:0048037∼cofactor binding (32)	hsa03320:PPAR signaling pathway (13)	phosphoprotein (129)	Placenta (75)
GO:0006631∼fatty acid metabolic process (23)	GO:0031090∼organelle membrane (49)	GO:0008289∼lipid binding (22)	hsa04910:Insulin signaling pathway (13)	acetylation (71)	Liver (72)
GO:0032868∼response to insulin stimulus (13)	GO:0000267∼cell fraction (48)	GO:0031406∼carboxylic acid binding (19)	hsa00071:Fatty acid metabolism (8)	oxidoreductase (43)	Skin (48)
GO:0010876∼lipid localization (8)	GO:0005829∼cytosol (48)	GO:0000287∼magnesium ion binding (15)	hsa01040:Biosynthesis of unsaturated fatty acids (6)	transferase (32)	Adipose tissue (17)
GO:0055088∼lipid homeostasis (7)	GO:0005783∼endoplasmic reticulum (44)	GO:0019842∼vitamin binding (14)	hsa04920:Adipocytokine signaling pathway (6)	lipoprotein (16)	Fetal liver (11)
GO:0006869∼lipid transport (7)	GO:0031975∼envelope (31)	GO:0009055∼electron carrier activity (11)	hsa00564:Glycerophospholipid metabolism (6)	Apoptosis (14)	Fetal brain cortex (11)
GO:0030258∼lipid modification (6)	GO:0005794∼Golgi apparatus (30)	GO:0005504∼fatty acid binding (9)	hsa00062:Fatty acid elongation in mitochondria (4)	Acyltransferase (7)	Adipocyte (8)
GO:0034440∼lipid oxidation (5)	GO:0005615∼extracellular space (22)	GO:0004091∼carboxylesterase activity (7)	hsa00061:Fatty acid biosynthesis (3)	diabetes mellitus (6)	
GO:0045444∼fat cell differentiation (5)	GO:0005792∼microsome (16)	GO:0016229∼steroid dehydrogenase activity (5)			
GO:0010883∼regulation of lipid storage (4)	GO:0009986∼cell surface (12)				

Cluster 2					
Gene ontologies	Cellular compartmentalization	Molecular function	KEGG signaling pathways	Functional categories	Expression site
GO:0007155∼cell adhesion (14)	GO:0005886∼plasma membrane (63)	GO:0005509∼calcium ion binding (21)	hsa04060:Cytokine-cytokine receptor interaction (10)	glycoprotein (93)	Placenta (44)
GO:0008283∼cell proliferation (13)	GO:0005576∼extracellular region (39)	GO:0030246∼carbohydrate binding (13)	hsa05200:Pathways in cancer (9)	phosphoprotein (90)	Liver (32)
GO:0007267∼cell-cell signaling (13)	GO:0009986∼cell surface (9)	GO:0019838∼growth factor binding (9)	hsa04612:Antigen processing and presentation (7)	disulfide bond (63)	Uterus (26)
GO:0048545∼response to steroid hormone stimulus (8)	GO:0043235∼receptor complex (5)	GO:0005125∼cytokine activity (7)	hsa04020:Calcium signaling pathway (6)	transmembrane protein (21)	Skin (26)
GO:0001775∼cell activation (8)		GO:0005543∼phospholipid binding (6)	hsa04940:Type I diabetes mellitus (5)	lipoprotein (15)	Kidney (23)
GO:0006935∼chemotaxis (7)		GO:0005539∼glycosaminoglycan binding (5)	hsa04115:p53 signaling pathway (4)	cytokine (7)	Pancreas (16)
GO:0000165∼MAPKKK cascade (6)				Lectin (6)	Endothelial cell (4)
GO:0043627∼response to estrogen stimulus (5)				membrane protein (4)	Myometrium (2)
GO:0031960∼response to corticosteroid stimulus (4)					

Cluster 3					
Gene ontologies	Cellular compartmentalization	Molecular function	KEGG signaling pathways	Functional categories	Expression site
GO:0045449∼regulation of transcription (52)	GO:0005886∼plasma membrane (77)	GO:0008270∼zinc ion binding (46)	hsa04514:Cell adhesion molecules (CAMs) (12)	alternative splicing (145)	Brain (133)
GO:0042127∼regulation of cell proliferation (26)	GO:0005576∼extracellular region (45)	GO:0030528∼transcription regulator activity (35)	hsa05200:Pathways in cancer (12)	phosphoprotein (143)	Placenta (64)
GO:0007155∼cell adhesion (23)	GO:0000267∼cell fraction (25)	GO:0003723∼RNA binding (18)	hsa04510:Focal adhesion (9)	glycoprotein (90)	Liver (55)
GO:0007049∼cell cycle (19)	GO:0044451∼nucleoplasm part (15)	GO:0008233∼peptidase activity (15)	hsa04612:Antigen processing and presentation (7)	disulfide bond (68)	Epithelium (51)
GO:0006928∼cell motion (18)	GO:0009986∼cell surface (13)	GO:0003682∼chromatin binding (7)	hsa04144:Endocytosis (7)	acetylation (48)	Skin (35)
GO:0051276∼chromosome organization (16)	GO:0000785∼chromatin (8)	GO:0005518∼collagen binding (5)	hsa04940:Type I diabetes mellitus (6)	transcription regulation (41)	Uterus (34)
GO:0016477∼cell migration (13)	GO:0005773∼vacuole (8)	GO:0003690∼double-stranded DNA binding (5)	hsa04512:ECM-receptor interaction (5)	chromosomal rearrangement (15)	Bone marrow (20)
GO:0051674∼localization of cell (13)	GO:0000228∼nuclear chromosome (6)	GO:0008266∼poly(U) RNA binding (2)		DNA damage (7)	Plasma (11)
GO:0040008∼regulation of growth (12)				growth regulation (4)	Fetal kidney (9)

Cluster 4					
Gene ontologies	Cellular compartmentalization	Molecular function	KEGG signaling pathways	Functional categories	Expression site
GO:0006350∼transcription (39)	GO:0043228∼non-membrane-bounded organelle (41)	GO:0003677∼DNA binding (43)	hsa05200:Pathways in cancer (7)	phosphoprotein (148)	Brain (111)
GO:0007242∼intracellular signaling cascade (23)	GO:0005856∼cytoskeleton (24)	GO:0030528∼transcription regulator activity (25)	hsa04530:Tight junction (5)	alternative splicing (115)	Epithelium (76)
GO:0007049∼cell cycle (20)	GO:0031981∼nuclear lumen (20)	GO:0003779∼actin binding (8)	hsa04370:VEGF signaling pathway (3)	acetylation (45)	Testis (72)
GO:0051276∼chromosome organization (12)	GO:0005654∼nucleoplasm (14)	GO:0032403∼protein complex binding (6)	hsa04630:Jak-STAT signaling pathway (3)	transcription regulation (39)	Placenta (66)
GO:0016568∼chromatin modification (8)	GO:0005694∼chromosome (11)	GO:0046332∼SMAD binding (3)	hsa04010:MAPK signaling pathway (3)	dna-binding (33)	Uterus (35)
GO:0043549∼regulation of kinase activity (8)	GO:0005813∼centrosome (8)			cell cycle (11)	Fetal brain (16)
GO:0007050∼cell cycle arrest (5)	GO:0031012∼extracellular matrix (8)			chromatin regulator (8)	Fetal kidney (11)
GO:0000075∼cell cycle checkpoint (4)	GO:0005874∼microtubule (7)			methylation (8)	Fibroblast (5)

The genes of each cluster were uploaded individually to online databases (DAVID and KEGG) and analyzed for their link to different biological parameters like gene ontology, cellular compartmentalization, molecular function, signaling pathway and site of expression. The parameters were selected on the basis of the enrichment score and relevance for adipogenesis. The numbers given in brackets are the numbers of genes associated to the corresponding GO term and signaling pathways.

**Figure 4:**
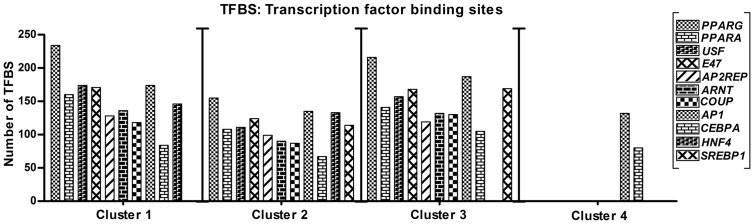
Transcription factor binding sites (TFBS) analysis. Analysis of transcription factor binding sites (TFBS) was performed and the selected adipogenic-specific TFBS showed most of the binding sites in cluster 1–3 genes and only a few significant sites in cluster 4 genes.

Our next step was based on the knowledge that transcription factors play a key role in the induction and regulation of adipogenesis. First, we were interested in the number of their binding sites (TFBS). Thus, applying the DAVID tool for TFBS determination, we analyzed the TFBS set and its corresponding transcription factor set of each cluster (Suppl. Table S2). Then, with the help of the National Centre for Biotechnology Information (NCBI) database PubMed and DAVID, all these TFBS and transcription factors were sorted and analysed regarding their possible influencing role in adipogenesis. Here, on the basis of effective relation to adipose tissue development, we selected a panel of adipogenesis related transcription factors like *activator protein-1* (*AP1*), *aryl hydrocarbon receptor nuclear translocator* (*ARNT*), C*CAAT/enhancer binding protein-α* (*C/EBPA*), *hepatocyte nuclear factor-4* (*HNF4*), *kruppel-like factor-12* (*KLF12* or *AP2REP*), *nuclear receptor subfamily-2*, *group F*, *member 2* (*NR2F2* or *COUPTFII*), *PPARA*, *PPARG*, *transcription factor-3* (*TCF3* or *E47*), *sterol regulatory element binding protein-1* (*SREBP1*) and *upstream transcription factor-1* (*USF*) (Suppl. Table S2). As these factors are well known in the context of adipogenesis, we conclude that by TFBS screening and application of the appropriate analytical tools, we have found significant binding sites for several important transcription factors involved in adipogenic development. Strikingly, most of the TFBS for these transcription factors were found in clusters 1–3 but not in cluster 4 (Figure 4). In more detail, only *AP1* and *C/EBPA* have binding sites in all 4 clusters. *ARNT*, *KLF12*, *NR2F2*, *TCF3*, *PPARA*, *PPARG and USF* have binding sites in clusters 1–3, *HNF4* in clusters 1 and 2 and *SREBP1* in clusters 2 and 3.

### Association of cluster genes with adipogenesis relevant signaling pathways

**Figure 5:**
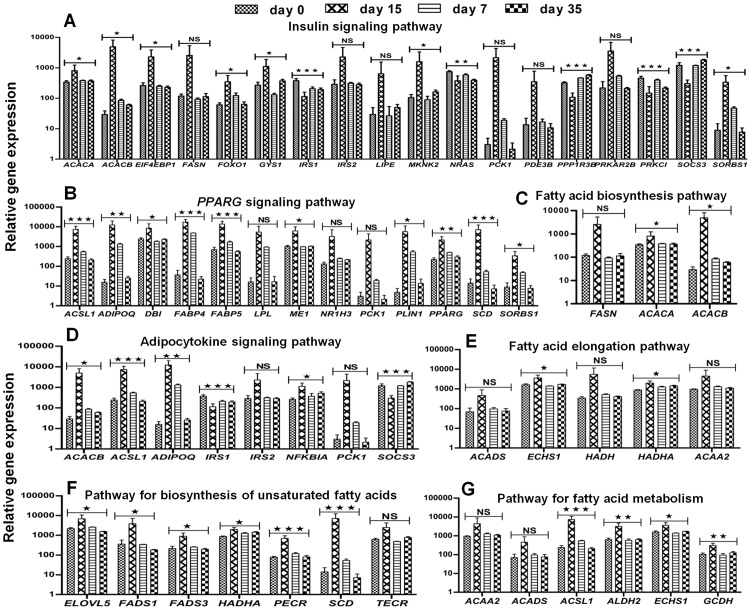
Analysis of adipogenic-specific signaling pathways. The 991 genes that were differentially expressed during adipogenesis were uploaded to the KEGG database to determine their involvement in adipogenic signaling pathways. We found different signaling cascades for adipogenesis like the (**A**) insulin signaling pathway, (**B**) *PPARG* signaling pathway, (**C**) fatty acid biosynthesis pathway, (**D**) adipocytokine signaling pathway, (**E**) fatty acid elongation pathway, (**F**) pathway for biosynthesis of unsaturated fatty acids and (**G**) pathway for fatty acid metabolism. Error bars, Means ± S.E.M.; *P<0.05; **P<0.01; ***P<0.001, NS, not significant (One way ANOVA, performed for statistical analysis).

Next, the gene lists of the 4 clusters were screened for genes known in the context of adipogenesis relevant signaling pathways. Here, the KEGG portal of DAVID was used. Many insulin signaling pathway genes were differentially expressed during adipogenesis. On day 15 (differentiated state), the expression of *ACACA*, *ACACB*, *EIF4EBP1*, *FASN*, *FOXO1*, *GYS1*, *IRS1*, *IRS2*, *LIPE*, *MKNK2*, *PCK1*, *PDE3B*, *PRKAR2B* and *SORBS1* was up-, and of *NRAS*, *PPP1R3B*, *PRKCI* and *SOCS3* was downregulated (Figure 5A; Suppl. Table S1 for detailed data). During dedifferentiation, on day 7 (early state) and day 35 (late state) the expression value of all these genes was almost reversed to the level on day 0 (undifferentiated state). In addition, many genes of the *PPARG* signaling pathway were differentially expressed during adipogenesis. From day 0 to day 15, the expression of *ACSL1*, *ADIPOQ*, *DBI*, *FABP4*, *FABP5*, *LPL*, *ME1*, *NR1H3*, *PCK1*, *PLIN1*, *PPARG*, *SCD* and *SORBS1* was up-, and in the time course of dedifferentiation downregulated to about the level on day 0 (Figure 5B). *ACACA*, *ACACB* and *FASN* are members of the fatty acid biosynthesis signaling pathway, whose expression was significantly upregulated during adipogenesis and reverted to the level in undifferentiated MSC during dedifferentiation (Figure 5C). *ACACB*, *ACSL1*, *ADIPOQ*, *IRS1*, *IRS2*, *NFKBIA*, *PCK1* and *SOCS3* belong to the adipocytokine signaling pathway and were differentially expressed between day 0 and day 15 (Figure 5D). Clearly, during dedifferentiation their expression values were changed in such a way that they almost become similar to the level of undifferentiated MSC. Also genes of the fatty acid elongation pathway, such as *ACADS*, *ECHS1*, *HADH*, *HADHA* and *ACAA2* (Figure 5E), and of the pathway for biosynthesis of unsaturated fatty acids, like *ELOVL5*, *FADS1*, *FADS3*, *HADHA*, *PECR*, *SCD* and *TECR* (Figure 5F), were differentially expressed (upregulated) during adipogenesis. During dedifferentiation, their expression was downregulated and nearly reached the level on day 0. Similarly, the gene expression of *ACAA2*, *ACADS*, *ACSL1*, *ALDH2*, *ECHS1* and *GCDH*, members of the fatty acid metabolic pathway, was upregulated in adipogenic differentiated cells (Figure 5G), and during dedifferentiation almost comes back to the expression level of undifferentiated MSC; the cells they are derived from.

Interestingly, about all of the genes reported here in the context of adipogenic signaling pathways belong to clusters 1–3 but not cluster 4. In summary, it can be stated that based on Table 1 data, transcription factors and its TFBS (Figure 4, Suppl. Table S2), and on participation in signaling pathways (Figure 5), clusters 1–3 are the relevant ones in context of adipogenesis. Thus, in line with our hypothesis and confirming the benefit of our approach of extended adipogenesis, possible true adipogenic marker genes belong to clusters, in which the expression during dedifferentiation was reverted to the undifferentiated state. Fat marker selection only on the basis of significantly changed gene expression as a result of induction with insulin, dexamethasone, indomethacin and IBMX would be misleading. Finally, cluster 1 included about all prominent adipogenic and fat markers, and according to the results had more association with adipogenesis relevant terms and features than clusters 2 and 3.

### Selection and analysis of new marker genes for adipogenesis

991 genes were differentially expressed during adipogenic differentiation of human MSC and therefore, presented possible marker genes to describe adipogenesis (Suppl. Table S1). These genes were subdivided into 4 groups or K-means clusters (Figure 3A–D) with 307 (cluster 1), 198 (cluster 2), 277 (cluster 3) and 209 genes (cluster 4). As shown, the expression of cluster 4 genes was not reverted to the undifferentiated state during dedifferentiation. On the contrary, after 35 days in dedifferentiation culture they had the expression values of adipogenic differentiated cells. So, according to our approach, we excluded the 209 cluster 4 genes and therefore, the marker list could be narrowed down to the 782 cluster 1–3 genes. For the determination of already known and new markers, this list was analyzed applying different bioinformatics tools of the online databases DAVID, Information Hyperlinked Over Proteins (iHOP) [30], KEGG, PubMed and WikiGenes [31].

Genes were considered as already known markers, if according to these databases they are directly associated with terms like adipogenesis, lipid or fat. As a result, we obtained a list of 185 possible marker genes, which have already been published in the context of adipogenic development and adipose tissue (Suppl. Table S1). Since we were interested in new marker genes, we excluded these 185 genes. This resulted in 597 genes (Suppl. Table S1), which were sorted according to their main fold change value in adipogenesis (first priority), and searched gene by gene for an indirect association with adipogenesis (exclusion criterion).

**Figure 6:**
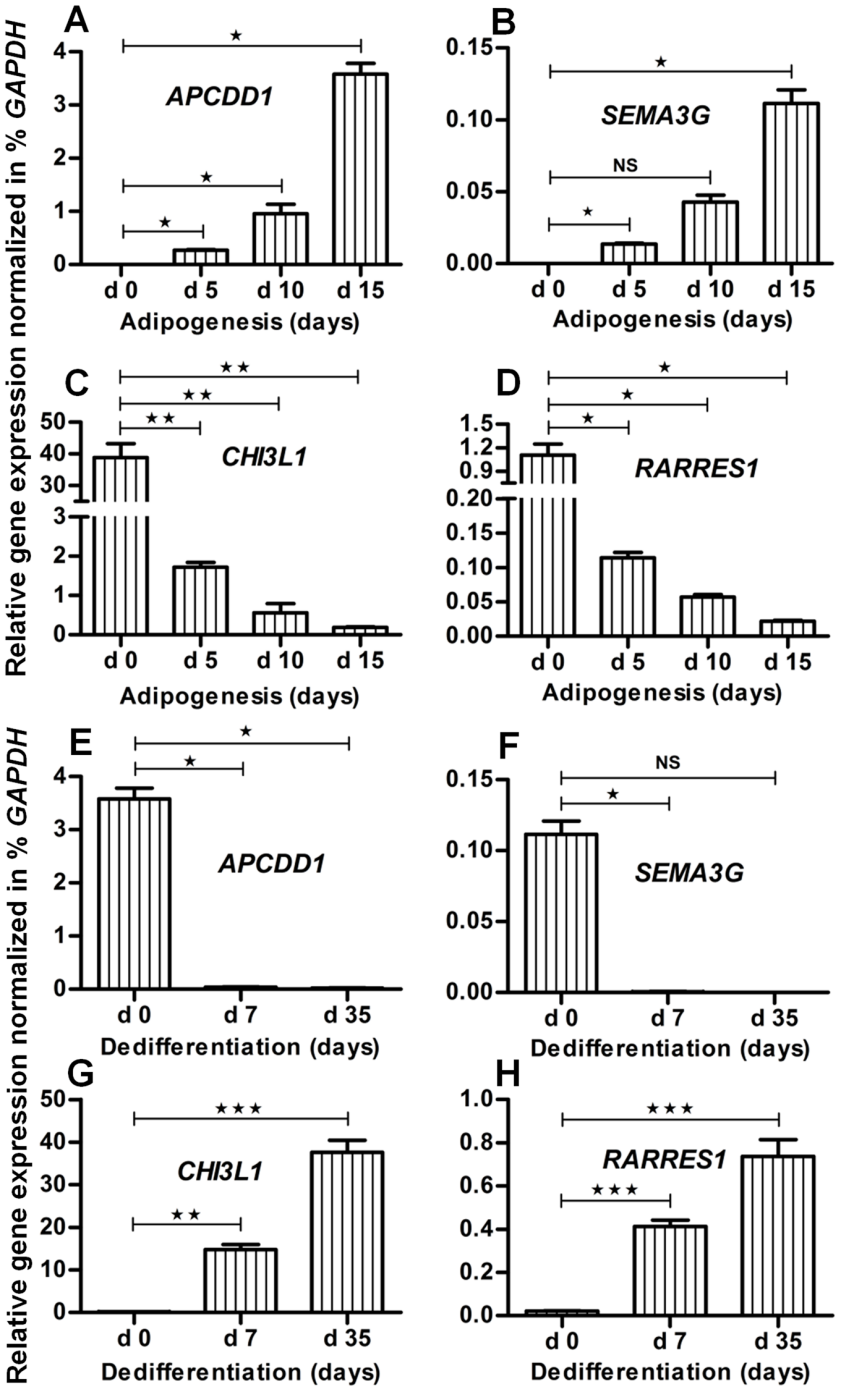
New potential fat marker genes, selected based on the coupling model of adipogenesis and reverse adipogenesis. Gene expression analysis was performed using qRT-PCR and the expression values were normalized to *GAPDH* for stepwise assessment of adipogenesis and reverse adipogenesis (dedifferentiation). Gene expression of new potential fat marker genes (**A**) *APCDD1*, (**B**) *SEMA3G*, (**C**) *CHI3L1* and (**D**) *RARRES1* is given for different stages of adipogenesis, i.e. at day 5, day 10 and day 15. Similarly, the expression of (**E**) *APCDD1*, (**F**) *SEMA3G*, (**G**) *CHI3L1* and (**H**) *RARRES1* is given for different stages of dedifferentiation (reverse adipogenesis). Here the gene expression of adipogenic differentiated cells is represented by day 0 as a reference for dedifferentiation. Error bars, Means ± S.E.M (n = 3); *P<0.05; **P<0.01; ***P<0.001, NS, not significant (student t test, performed for statistical analysis).

As a result, we selected the 4 genes *APCDD1*, *CHI3L1*, *RARRES1* and *SEMA3G* as possible new marker genes for the verification and description of adipogenesis (Suppl. Figure S1). Then, their usability was validated applying qRT-PCR. Nine adipogenic cultures (15 days) were analyzed and showed a consistent and reproducible expression of all 4 markers genes (Suppl. Figure S2). Finally, the adipogenesis and dedifferentiation cultures, which were used for GeneChip experiments, were qRT-PCR analyzed. For the fat markers *PPARG* and *FABP4* the results were already presented in Figure 2. Regarding the new markers, during adipogenesis of human MSC the expression of *APCDD1* (Figure 6A) and *SEMA3G* (Figure 6B) in relation to the expression of the housekeeping gene *GAPDH* was continuously up-, and of *CHI3L1* (Figure 6C) and *RARRES1* (Figure 6D) downregulated from day 0 until day 15. During dedifferentiation of adipogenic differentiated cells, the expression of all 4 new markers was reverted. The expression of *APCDD1* (Figure 6E) and *SEMA3G* (Figure 6F) in relation to *GAPDH* was significantly down-, and of *CHI3L1* (Figure 6G) and *RARRES1* (Figure 6H) upregulated from day 0 (start of dedifferentiation culture) to day 35. In conclusion, we found and validated 4 new possible marker genes, which so far have not been published in the context of adipogenesis.

## Discussion

The aim of this study was to analyze the adipogenic differentiation of MSC and to discover potential new adipogenic-specific marker genes. For the first time, this aim should be achieved not only by cell differentiation but also by reversing this process by dedifferentiation. In this regard, MSC were isolated [2], [23], differentiated into adipogenic lineage cells [23] and finally were dedifferentiated (reverse adipogenesis). Here, bone marrow-derived MSC were used instead of fat tissue-derived MSC with similar properties. The most important reason was that fat tissue-derived MSC potentially are already primed into the adipogenic lineage and express genes relevant for adipogenesis without adding an adipogenic cocktail. Another reason was that bone marrow-derived MSC have already been used in several studies in the context of genome-wide expression profiling and regenerative medicine [2], [12], [13]. Both adipogenesis and reverse adipogenesis were confirmed on histological level by Oil Red O staining and on molecular level by qRT-PCR of the adipogenic marker genes *PPARG* and *FABP4*. Furthermore, genome-wide microarrays were performed to evaluate our hypothesis that by reversing adipogenesis (dedifferentiation) the adipogenic-specific genes alter their expression and resume to a level comparable to undifferentiated MSC. Such genes may reflect a real image of adipogenesis. In this context, we selected 991 genes with significantly changed expression during the course of adipogenesis. Then, we compared the expression of these genes with their expression during dedifferentiation. Subsequently, the list of 991 genes was divided into 4 clusters by K-means clustering on the basis of their expression values to facilitate the evaluation process for a profound insight into adipogenesis.

Overall, cluster 1 showed the highest relevance for adipogenesis, followed by clusters 2 and 3, while cluster 4 showed no or very minute associations with this differentiation lineage. Cluster 1 genes were upregulated during adipogenesis and downregulated during dedifferentiation. Applying web-based tools for text mining revealed an influence of many genes like *PPARG*, *FABP4*, *LPL*, *LIPE*, *ADIPOQ*, *PLIN1*, *PLIN4*, *IRS2*, *C/EBPA*, *APOE* and *APOL2* on diverse adipogenic events [11], [29], [30], which supports our conclusion that cluster 1 genes have major relevance to adipogenesis. For instance, *PPARG* is a well known adipogenic target and acts as a central hub among different signaling cascades to regulate and fine tune the adipogenic differentiation of MSC [11]. *FABP4* takes part in the predisposition of cardiac fats in obese persons [32], and *ADIPOQ* upregulation is the main cause of type 2 diabetes and obesity [33]. Cluster 2 and 3 genes were downregulated during differentiation and upregulated during dedifferentiation to their level in undifferentiated cells (cluster 2 at day 35, cluster 3 at day 7). Some genes like *PARP4* and *SOCS3* found in these clusters were already known to have relevance for adipogenesis. The downregulated expression of *PARP4* and *SOCS3* makes it inhibitory targets for adipogenesis, and also negatively regulates the process of adipogenesis [34], [35]. Moreover, application of web-based tools for text mining showed both a positive and negative correlation of cluster 2 and 3 genes to fat formation, regulation and metabolism [30], [36], [37], [38], and therefore indicates the association of above cluster genes to adipogenesis. Finally, again using web-based tools for text mining, for cluster 4 genes like *RB1*, *STAG1*, *DST*, *NPAT*, *CGGBP1*, *SMAD5*, *ARID4B*, *NCOA7* and *NR3C1*, we found high enrichment scores for biological annotations like cell cycle, transcription and chromosomal reorganization [27], [30], [39]. For instance, *STAG1* is a cell cycle regulator and its overexpression is reported for breast cancer and cellular proliferation [40], while the methylation of *RB1* by *SMYD2* enhances cell cycle progression [39]. The expression of cluster 4 genes was not assignable to a typical differentiation or dedifferentiation lineage. Expression values were downregulated during differentiation, upregulated to the undifferentiated expression level at day 7 of dedifferentiation and again changed at day 35 to a level of the differentiated cells. Therefore, the option arises that genes in cluster 4 are not regulated due to an adipogenic induction but according to an independent regulation mechanism. Genes like *RB1*, *STAG2*, *HAUS6*, *MSH2*, *TLK1*, *AEBP2* and *CAND1* may be involved in the reorganization and inter-conversion of the different states of cells. Text mining revealed a biological association of chromosomal reorganization with cluster 4 genes [27], [30], [37], and thus strengthen our speculative interpretation. Another possible explanation is that also these genes are important for adipogenesis but are downregulated to maintain the undifferentiated state of the reverse differentiated adipocytes. Alternatively, it also seems possible that some of them may reflect a state of replicative senescence, as *RB1*, *STAG2* and *CAND1* are well known cell cycle regulators [39], [40], [41].

Transcription factors are considered to be crucial for adipogenesis [42]. These factors control the flow of genetic information and regulate most cellular processes by binding to specific sequences of DNA [43], [44]. Thus applying different bioinformatic tools [27], [30], [45], we showed the expression of several prominent adipogenic transcription factors like *PPARG*, *PPARA*, *USF*, *E47*, *AP2REP*, *ARNT* and *COUP*. By analysis of their binding sites, we showed TFBS in clusters 1–3, and no sites in cluster 4 genes. Similarly, *HNF4* showed TFBS in clusters 1 and 2 while the TFBS of *SREBP1* were present in clusters 2 and 3 instead of cluster 1 genes. In addition, the transcription factors *AP1* and *C/EBPA* showed binding sites not only in clusters 1–3 but also in cluster 4, even though *C/EBPA* having affiliation with adipogenesis [46]. However, *C/EBPA* needs *PPARG* sites for its functional activation [46]. Due to the fact that we found no *PPARG* binding sites in cluster 4 genes, it further emphasizes that these genes have only a very minor or no role in adipogenesis. TFBS analysis provides a prompt overview about any cellular process [43], [44], [45], therefore based on it, clusters 1–3 include more genes involved in adipogenesis compared to cluster 4.

Normally, signaling pathways are considered to interplay a vital role during any cellular process via significant alteration in their gene expression [47]. For adipogenesis, signaling pathways facilitate a controlling and regulating mechanism to fine tune the overall process [11], [47]. We analyzed and interpreted our results using the online analytical tool of the KEGG database [29], [48]. The expressed transcripts showed a profound crosstalking among different signaling pathways and represented a relevance to adipogenesis. In this regard, the insulin signaling pathway is critical to regulate the carbohydrate metabolism in response to body's demand of energy. Furthermore, its ability for glucose uptake, consumption and distribution makes it one of the crucial signaling events for diabetics [10], [49] and adipogenesis [50]. The *PPARG* signaling pathway plays an essential and comparatively more influencing role than any other known signaling pathways in the context of adipogenesis [51]. It controls and operates the overall cellular process of fat formation and also plays a unique role in fine tuning the process of adipogenesis [11]. In addition to these pathways, we also found many genes involved in the fatty acid biosynthesis pathway, the adipocytokine signaling pathway, the fatty acid elongation pathway, the pathway for biosynthesis of unsaturated fatty acids and pathway for fatty acid metabolism. Most of the genes found in these signaling pathways were highly expressed during adipogenesis and decreased their expression to a level similar to undifferentiated MSC. In this way, we could broadly verify the differentiation of MSC towards the adipogenic lineage and their subsequent dedifferentiation.

To study a cellular process by a reverse approach is not new in the scientific community [52]. Using this reverse approach for adipogenesis for the first time we generated a more detailed image of adipogenic differentiation and found that the selection of adipogenic-specific genes only on the basis of significant expression during adipogenesis is not sufficient and could be misleading. Therefore, as a consequence of our approach that coupled the processes of adipogenesis and reverse adipogenesis, cluster 4 genes were excluded because of their minute or almost no association with adipogenesis. We identified only 782 genes out of total 991 significantly expressed genes, which reflect a real image of adipogenesis.

Our study supports most of the genes from previously published studies that describe significantly changed expressions during adipogenesis [12], [13], [47]. Nevertheless, the selection method for so far selected fat markers, which are just based on significant changes during gene expression, is not sufficient. On the basis of our approach, we selected 4 new possible fat marker genes (*APCDD1*, *CHI3L1*, *RARRES1* and *SEMA3G*) for the verification and description of adipogenesis that show high changes in gene expression but are so far known not yet to be involved in adipogenesis. Overexpression of *APCDD1* is reported in context of colorectal carcinogenesis [53], and also known for its inhibitory effect on the WNT signaling pathway [54]. This pathway takes part in the regulation, development and metabolism of adipose tissue [55]. In addition, WNT signaling is an essential requirement for the conversion of MSC into preadipocytes [56]. Thus, *APCDD1* is indirectly related with adipogenesis or is a negative regulator of adipogenic differentiation. *SEMA3G* is another potential marker for adipogenesis, has an inhibitory effect on tumor progression [57], and takes part in controlling the function of endothelial cells and smooth muscle cells [58]. *CHI3L1* encodes a glycoprotein that takes part in macrophage differentiation [59] and has an association with chondrocytes but no association with rheumatoid arthritis [60]. *RARRES1* is a retinoic acid receptor that acts as a vital tumor suppressor gene [61]. Its downregulation is reported for cancer by interacting with *ATP/GTP binding protein-like 2* (*AGBL2*) [62]. Apart from this, it also takes part in proliferation processes and in nasopharyngeal carcinoma [63]. Retinoic acid is known for suppressing adipogenesis and obesity by promoting energy consumption [64]. By using the current web-based tools for text mining [27], [30], [65], the 4 potential marker genes showed no direct connection to adipogenesis. Based on their expression pattern as well as on the coupling approach of adipogenesis and reverse adipogenesis, *APCDD1*, *CHI3L1*, *RARRES1* and *SEMA3G* are potential marker genes for the analysis of adipogenic processes.

Besides this, the reversion of adipogenesis, dedifferentiation, could be a promising approach for the treatment of obesity and their correlated problems. This reversing approach of adipogenesis also advocates soft tissue engineering with a new therapeutic angle, and will also open new doors for further studies in this direction.

## Conclusions

Adipogenic marker genes are generally selected on the basis of a significant change in their expression during adipogenic differentiation. Generally this selection is misleading, because the adipogenesis inducing cocktail not only induces the expression of adipogenic-specific genes but also the expression of genes for involved in other cellular processes. So, how to filter adipogenic-specific genes out of all differentially expressed genes needs an answer. To achieve this, we combined the process of adipogenesis with reverse adipogenesis. During adipogenesis, 991 genes were significantly expressed, and according to our hypothesis some of these genes not represent the process of adipogenesis. Therefore, to filter adipogenic-specific genes, we reversed the expression of adipogenic genes by reverse adipogenesis and in this way, we selected more relevant fat marker genes. On the basis of this approach, we filtered 782 genes out of total 991 significantly expressed genes. To validate the benefit of our approach, we analyzed all 991 genes for adipogenic-linked biological annotations, adipogenic transcription factors and adipogenic signaling pathway. Interestingly, genes from our filtered 782 fat markers, such as the most prominent adipogenic marker genes *PPARG*, *FABP4*, *LPL*, *LIPE*, *ADIPOQ*, *PLIN1*, *PLIN4*, *IRS2*, *C/EBPA*, *APOE* and *APOL2*, showed a much stronger affiliation to adipogenesis than the other 209 genes. Clearly, this shows the usefulness and importance of our approach. Furthermore, we identified *APCDD1*, *CHI3L1*, *RARRES1* and *SEMA3G* as potential adipogenic-specific marker genes by using the model of adipogenesis and reverse adipogenesis.

## Supporting Information

Figure S1 **Microarray gene expression profile of potential new fat marker genes during adipogenesis and reverse adipogenesis.** Microarray gene expression analysis was performed for potential new fat marker genes (n = 3 donors) during adipogenesis and reverse adipogenesis (dedifferentiation). Relative gene expression of new introductory fat marker genes of (**A**) *APCDD1*, (**B**) *SEMA3G*, (**C**) *CHI3L1* and (**D**) *RARRES1* is given for different donors (n = 3). Error bars, Means ± S.E.M (n = 3).
(TIF)

Figure S2 **Gene expression profile validation of new fat marker genes via qRT-PCR for different individual donors (n = 9).** Gene expression analysis of potential new fat marker genes was performed using qRT-PCR for individual donors (n = 9). Gene expression of new introductory fat marker genes of (**A**) *APCDD1*, (**B**) *SEMA3G*, (**C**) *CHI3L1* and (**D**) *RARRES1* is given for 9 different donors. The gene expression was normalized to % *GAPDH* expression.
(TIF)

Figure S3 **Figure Of Merit (FOM) analysis.** The 991 selected genes, which were significantly expressed during adipogenesis as compared to undifferentiated MSC, were divided into 4 clusters on the basis of FOM. FOM classification of genes confirmed that only 4 cluster are significant, because as shown, any increase in cluster number didn't result in any significant cluster.
(TIF)

Table S1 **The selected 991 genes, differentially expressed during adipogenesis.** 991 candidate genes were selected on the basis of differentially expression in adipogenesis. Their mean signal expression values are given for different time points, i.e. undifferentiated MSC (day 0), adipogenic differentiated cells (day 15), early time point of dedifferentiated cells (day 7) and late time point of dedifferentiated cells (day 35). 991 genes were grouped into 4 clusters on the basis of K means classification. The genes in each cluster were organized according to ascending alphabetical order on the basis of gene symbol. ± std: standard deviation, MFC: mean fold change, In cluster 1–3 the gene symbol with ***asterix*** (_*_) representing published fat markers, while other without asterix are unpublished fat marker genes.
(DOCX)

Table S2 **Transcription factor binding sites (TFBS) included in each cluster.** The numbers of transcription factor binding sites (TFBS) are given in this table, and are organized according to ascending alphabetical order. The number in brackets represents the number of transcription factor binding sites (TFBS), and these TFBS are specific to each cluster gene as given in the Suppl. Table S1. For more detail of gene titles and expression values of the respective cluster genes, see Suppl. Table S1.
(DOCX)
