# Supplementary figures and images for: Reverse Differentiation as a Gene Filtering Tool in Genome Expression Profiling of Adipogenesis for Fat Marker Gene Selection and Their Analysis
Source: PLoS One. 2013 Jul 26;8(7):e69754. doi: 10.1371/journal.pone.0069754 (PMC3724870; doi:10.1371/journal.pone.0069754)

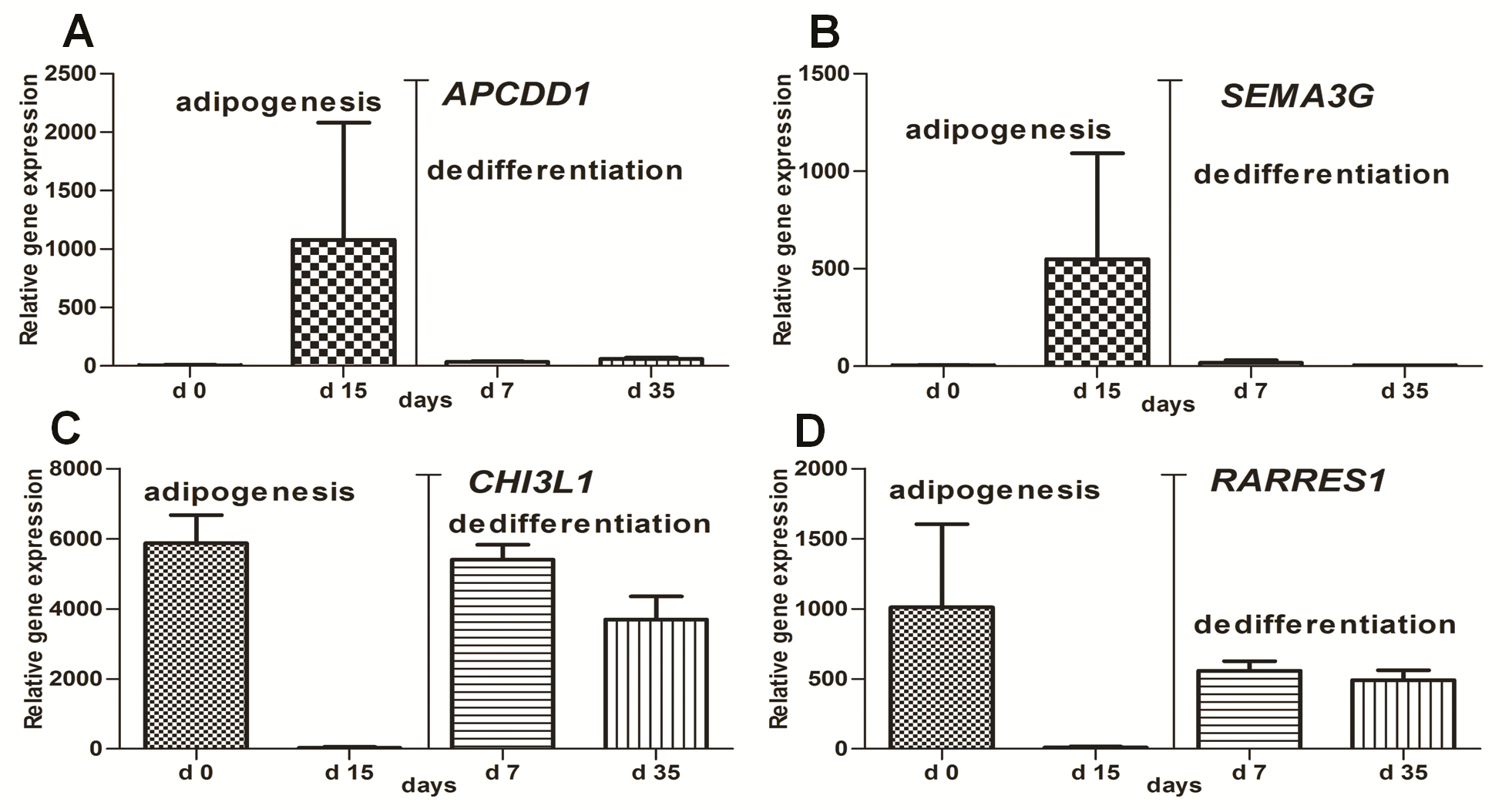

Supplement: Figure S1 — Microarray gene expression profile of potential new fat marker genes during adipogenesis and reverse adipogenesis. Microarray gene expression analysis was performed for potential new fat marker genes (n = 3 donors) during adipogenesis and reverse adipogenesis (dedifferentiation). Relative gene expression of new introductory fat marker genes of (A) APCDD1, (B) SEMA3G, (C) CHI3L1 and (D) RARRES1 is given for different donors (n = 3). Error bars, Means ± S.E.M (n = 3). (TIF) [file pone.0069754.s001.tif]

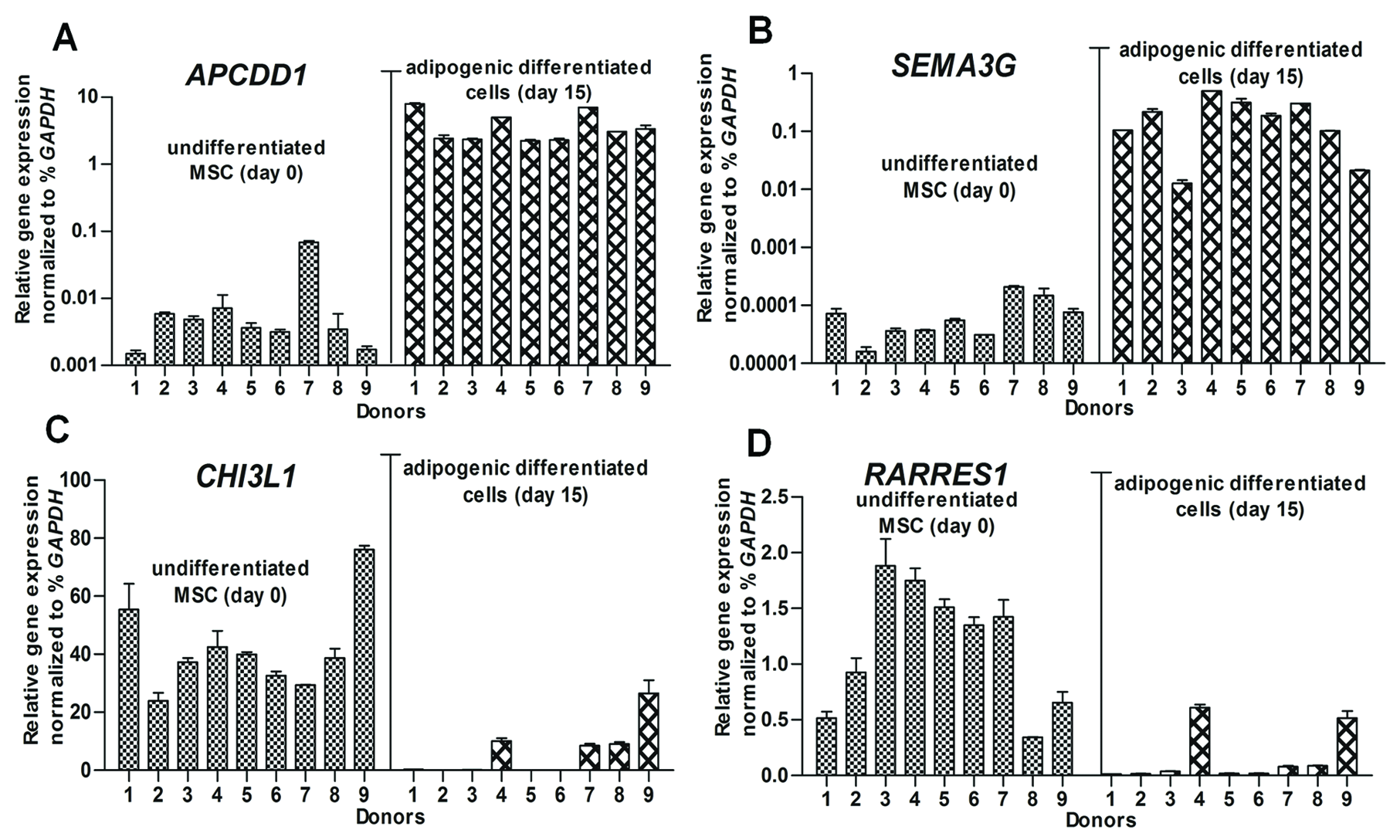

Supplement: Figure S2 — Gene expression profile validation of new fat marker genes via qRT-PCR for different individual donors (n = 9). Gene expression analysis of potential new fat marker genes was performed using qRT-PCR for individual donors (n = 9). Gene expression of new introductory fat marker genes of (A) APCDD1, (B) SEMA3G, (C) CHI3L1 and (D) RARRES1 is given for 9 different donors. The gene expression was normalized to % GAPDH expression. (TIF) [file pone.0069754.s002.tif]

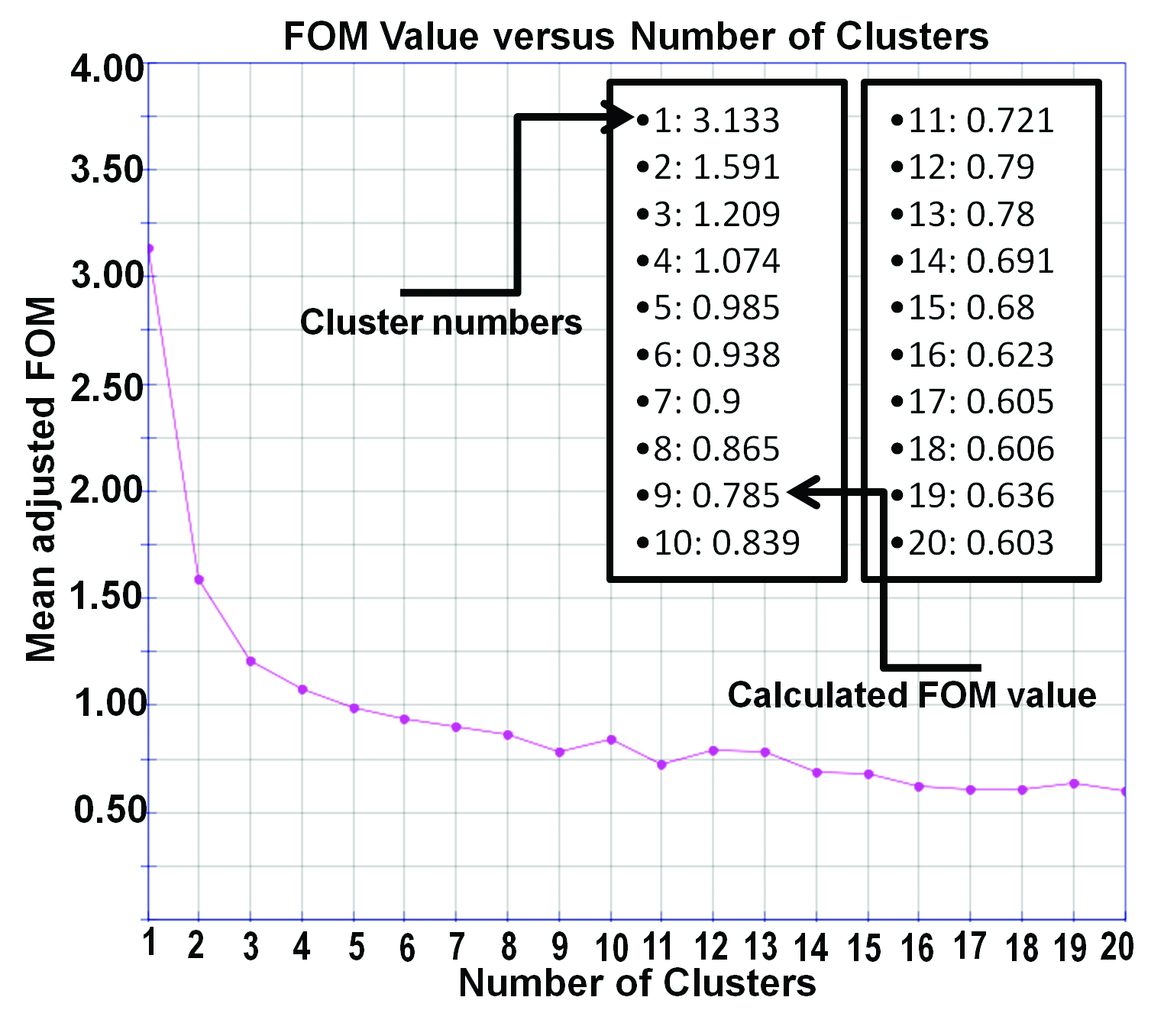

Supplement: Figure S3 — Figure Of Merit (FOM) analysis. The 991 selected genes, which were significantly expressed during adipogenesis as compared to undifferentiated MSC, were divided into 4 clusters on the basis of FOM. FOM classification of genes confirmed that only 4 cluster are significant, because as shown, any increase in cluster number didn't result in any significant cluster. (TIF) [file pone.0069754.s003.tif]
