# Supplementary material for: Reverse Differentiation as a Gene Filtering Tool in Genome Expression Profiling of Adipogenesis for Fat Marker Gene Selection and Their Analysis
Source: PLoS One. 2013 Jul 26;8(7):e69754. doi: 10.1371/journal.pone.0069754 (PMC3724870; doi:10.1371/journal.pone.0069754)
Supplement: Table S1 — The selected 991 genes, differentially expressed during adipogenesis. 991 candidate genes were selected on the basis of differentially expression in adipogenesis. Their mean signal expression values are given for different time points, i.e. undifferentiated MSC (day 0), adipogenic differentiated cells (day 15), early time point of dedifferentiated cells (day 7) and late time point of dedifferentiated cells (day 35). 991 genes were grouped into 4 clusters on the basis of K means classification. The genes in each cluster were organized according to ascending alphabetical order on the basis of gene symbol. ± std: standard deviation, MFC: mean fold change, In cluster 1–3 the gene symbol with asterix (*) representing published fat markers, while other without asterix are unpublished fat marker genes. (DOCX) [file pone.0069754.s004.docx]

# SUPPLEMENTARY TABLE

## Table S1. The selected 991 genes, differentially expressed during adipogenesis.

991 candidate genes were selected on the basis of differentially expression in adipogenesis. Their mean signal expression values are given for different time points, i.e. undifferentiated MSC (day 0), adipogenic differentiated cells (day 15), early time point of dedifferentiated cells (day 7) and late time point of dedifferentiated cells (day 35). 991 genes were grouped into 4 clusters on the basis of K means classification. The genes in each cluster were organized according to ascending alphabetical order on the basis of gene symbol. ± std: standard deviation, MFC: mean fold change, In cluster 1-3 the gene symbol with asterix (_*_) representing published fat markers, while other without asterix are unpublished fat marker genes.

| **Gene information** | | | | | | **Adipogenic differentiation** | | | **Dedifferentiation** | | |
| --- | --- | --- | --- | --- | --- | --- | --- | --- | --- | --- | --- |
| **Gene probe set ID** | **Gene title** | | **Gene symbol** | **MFC: d15 vs d0** | | **D0: signal value ± std** | | **D15: signal value ± std** | **D7: signal value ± std** | | **D35: signal value ± std** |
| **Cluster 1 genes** | | | | | | | | | | | |
| 203192_at | ATP-binding cassette, sub-family B (MDR/TAP), member 6 | | ABCB6 | | 2,96 | 123 ± 4,3 | | 426,9 ± 270,1 | 84,6 ± 11 | | 170,8 ± 19,7 |
| 226796_at | abhydrolase domain containing 15 | | ABHD15 | | 6,06 | 122,8 ± 9,4 | | 972,3 ± 700,5 | 104,7 ± 22,9 | | 211,2 ± 10,7 |
| 213017_at | abhydrolase domain containing 3 | | ABHD3 | | 2,37 | 89,3 ± 3,4 | | 251,4 ± 61,4 | 128,9 ± 30,6 | | 68,7 ± 3,5 |
| 202003_s_at | acetyl-Coenzyme A acyltransferase 2 | | ACAA2* | | 3,17 | 955,1 ± 48,9 | | 4438,6 ± 4471,5 | 1282,3 ± 111 | | 1082,3 ± 98,7 |
| 212186_at | acetyl-Coenzyme A carboxylase alpha | | ACACA* | | 2,79 | 339,1 ± 36,1 | | 809,6 ± 421,4 | 380,2 ± 12,6 | | 366 ± 32 |
| 43427_at | acetyl-Coenzyme A carboxylase beta | | ACACB* | | 148,17 | 29,2 ± 9,3 | | 4925,8 ± 3228,5 | 85,3 ± 7,9 | | 59,8 ± 4 |
| 202366_at | acyl-Coenzyme A dehydrogenase, C-2 to C-3 short chain | | ACADS* | | 5,08 | 69 ± 36,7 | | 458,2 ± 434 | 96,1 ± 16,4 | | 74,9 ± 26,6 |
| 207071_s_at | aconitase 1, soluble | | ACO1* | | 2,64 | 831,4 ± 71,9 | | 2478,7 ± 967,2 | 963,9 ± 42,8 | | 1098 ± 42,1 |
| 218844_at | acyl-CoA synthetase family member 2 | | ACSF2* | | 5,57 | 168,4 ± 15,5 | | 1598,3 ± 2078,5 | 309 ± 63,9 | | 192,2 ± 14,1 |
| 207275_s_at | acyl-CoA synthetase long-chain family member 1 | | ACSL1* | | 24,07 | 240,8 ± 59 | | 7316,2 ± 3383,8 | 536,4 ± 42,5 | | 207,8 ± 30,1 |
| 206833_s_at | acylphosphatase 2, muscle type | | ACYP2 | | 2,87 | 241,6 ± 48,4 | | 739,7 ± 440,9 | 269,7 ± 20,6 | | 278 ± 6,1 |
| 226814_at | ADAM metallopeptidase with thrombospondin type 1 motif, 9 | | ADAMTS9 | | 4,96 | 57,4 ± 12,8 | | 287,3 ± 112,1 | 77,9 ± 7,2 | | 32,7 ± 5,3 |
| 217761_at | acireductone dioxygenase 1 | | ADI1 | | 2,33 | 1052,9 ± 403,4 | | 2500,2 ± 319,2 | 1685,3 ± 19 | | 2312,3 ± 136,1 |
| 207175_at | adiponectin, C1Q and collagen domain containing | | ADIPOQ* | | 741,00 | 15,7 ± 5,8 | | 12202,2 ± 7193,9 | 1306,8 ± 128,2 | | 25,5 ± 5,4 |
| 201281_at | adhesion regulating molecule 1 | | ADRM1 | | 2,79 | 526,6 ± 86,1 | | 1705,9 ± 741,9 | 469,9 ± 18,8 | | 563 ± 28 |
| 226955_at | actin filament associated protein 1-like 1 | | AFAP1L1 | | 35,92 | 3,8 ± 3,2 | | 282,6 ± 203,2 | 29,5 ± 2,3 | | 3,7 ± 2,8 |
| 32837_at | 1-acylglycerol-3-phosphate O-acyltransferase 2 (lysophosphatidic acid acyltransferase, beta) | | AGPAT2* | | 7,13 | 210,4 ± 14 | | 2750,1 ± 3278,3 | 179,8 ± 12,8 | | 254,6 ± 9 |
| 224461_s_at | apoptosis-inducing factor, mitochondrion-associated, 2 | | AIFM2 | | 11,49 | 186,2 ± 10,6 | | 2995 ± 2073,5 | 192,9 ± 9,9 | | 202,7 ± 42,7 |
| 208967_s_at | adenylate kinase 2 | | AK2 | | 2,09 | 835,2 ± 74 | | 1817,5 ± 593,7 | 635,3 ± 81,2 | | 794,4 ± 89,7 |
| 217626_at | aldo-keto reductase family 1, member C1 | | AKR1C1* | | 3,85 | 125,9 ± 90,2 | | 383,8 ± 64,2 | 330,8 ± 15,5 | | 172,3 ± 21,3 |
| 205633_s_at | aminolevulinate, delta-, synthase 1 | | ALAS1 | | 3,82 | 556,9 ± 68,9 | | 2408,1 ± 1580,2 | 631,7 ± 35,3 | | 636,6 ± 42,8 |
| 205208_at | aldehyde dehydrogenase 1 family, member L1 | | ALDH1L1 | | 21,94 | 23,1 ± 9 | | 932,9 ± 558,7 | 51,3 ± 13,3 | | 20 ± 11,7 |
| 201425_at | aldehyde dehydrogenase 2 family (mitochondrial) | | ALDH2* | | 5,00 | 624,2 ± 134,4 | | 3147,6 ± 1591,8 | 578,8 ± 97,5 | | 637,3 ± 60,8 |
| 207064_s_at | amine oxidase, copper containing 2 (retina-specific) | | AOC2 | | 16,12 | 25,5 ± 7,1 | | 616,1 ± 545,1 | 51,7 ± 7,9 | | 38,7 ± 20,3 |
| 204894_s_at | amine oxidase, copper containing 3 (vascular adhesion protein 1) | | AOC3 | | 415,87 | 9,9 ± 3 | | 8737,4 ± 9338,4 | 219 ± 60,8 | | 22,9 ± 6,3 |
| 202399_s_at | adaptor-related protein complex 3, sigma 2 subunit | | AP3S2 | | 3,35 | 554,3 ± 42,4 | | 1820,8 ± 821,2 | 632,7 ± 26,1 | | 864,8 ± 23,5 |
| 230925_at | amyloid beta (A4) precursor protein-binding, family B, member 1 interacting protein | | APBB1IP | | 4,00 | 348,3 ± 134,2 | | 1581,6 ± 1254,9 | 1051,3 ± 82 | | 598,1 ± 38,5 |
| 225016_at | adenomatosis polyposis coli down-regulated 1 | | APCDD1 | | 153,99 | 4,7 ± 3,9 | | 1079,7 ± 1001 | 35,2 ± 6,4 | | 59,7 ± 11,1 |
| 204408_at | APEX nuclease 2 | | APEX2 | | 3,48 | 142 ± 34,2 | | 532,8 ± 384,4 | 106,8 ± 11,5 | | 130,4 ± 45,4 |
| 203382_s_at | apolipoprotein E | | APOE* | | 10,39 | 416,1 ± 332,1 | | 5053,2 ± 4565,8 | 2395,7 ± 143,4 | | 826,5 ± 40,5 |
| 221653_x_at | apolipoprotein L, 2 | | APOL2* | | 3,27 | 117 ± 34,3 | | 381,7 ± 185,1 | 178,7 ± 51,6 | | 143,4 ± 13,9 |
| 1555600_s_at | apolipoprotein L, 4 | | APOL4* | | 5,16 | 18,1 ± 9,9 | | 115,6 ± 51,3 | 73,3 ± 6,5 | | 34,2 ± 9,7 |
| 219716_at | apolipoprotein L, 6 | | APOL6* | | 3,27 | 105,6 ± 42,9 | | 380,6 ± 242,8 | 324 ± 7,7 | | 167,9 ± 12,3 |
| 229526_at | aquaporin 11 | | AQP11 | | 5,88 | 79,4 ± 32 | | 426,5 ± 109,3 | 105,4 ± 9,7 | | 83 ± 12,4 |
| 39248_at | aquaporin 3 (Gill blood group) | | AQP3 | | 10,24 | 46,2 ± 20,6 | | 825,5 ± 962,1 | 134,3 ± 12,9 | | 121,6 ± 25,6 |
| 205047_s_at | asparagine synthetase | | ASNS | | 5,12 | 305,6 ± 39,8 | | 2112,8 ± 2129,4 | 255,9 ± 16,3 | | 213,1 ± 27,7 |
| 207076_s_at | argininosuccinate synthetase 1 | | ASS1 | | 3,20 | 1548 ± 98,9 | | 5422,6 ± 2547,2 | 2212,5 ± 91,7 | | 1836,1 ± 37,8 |
| 203296_s_at | ATPase, Na+/K+ transporting, alpha 2 (+) polypeptide | | ATP1A2 | | 120,35 | 6,4 ± 6,1 | | 1498,6 ± 1494,2 | 39 ± 9,8 | | 16,7 ± 1,9 |
| 206992_s_at | ATP synthase, H+ transporting, mitochondrial F0 complex, subunit s | | ATP5S | | 3,01 | 75,7 ± 13,1 | | 310,9 ± 141,9 | 114,3 ± 17,9 | | 109,5 ± 29,6 |
| 209309_at | alpha-2-glycoprotein 1, zinc-binding | | AZGP1* | | 12,03 | 14,4 ± 1 | | 214,1 ± 131,1 | 20,2 ± 4 | | 16,5 ± 8,6 |
| 200837_at | B-cell receptor-associated protein 31 | | BCAP31 | | 2,46 | 1367,9 ± 283,8 | | 3432,7 ± 1648,9 | 1071,3 ± 53 | | 1668,3 ± 106,8 |
| 231810_at | BRI3 binding protein | | BRI3BP | | 3,82 | 66,2 ± 8,3 | | 291,2 ± 124,5 | 76,7 ± 13,9 | | 90,9 ± 9,1 |
| 218024_at | brain protein 44-like | | BRP44L | | 3,56 | 671,7 ± 59,8 | | 2672 ± 1297,4 | 773,4 ± 18,5 | | 842,9 ± 18,2 |
| 200920_s_at | B-cell translocation gene 1, anti-proliferative | | BTG1 | | 2,66 | 1519,2 ± 361,6 | | 4374,2 ± 1968,7 | 2338 ± 36,8 | | 2470,7 ± 8,6 |
| 201236_s_at | BTG family, member 2 | | BTG2 | | 6,60 | 129,4 ± 64,5 | | 694 ± 353,7 | 220,4 ± 22,3 | | 242,3 ± 43,3 |
| 224197_s_at | C1q and tumor necrosis factor related protein 1 | | C1QTNF1 | | 6,11 | 36,4 ± 19 | | 342,8 ± 131,6 | 66 ± 2 | | 126,5 ± 31,3 |
| 212875_s_at | C2 calcium-dependent domain containing 2 | | C2CD2 | | 2,68 | 415,8 ± 67 | | 1214,7 ± 656,7 | 586,4 ± 65,2 | | 451,3 ± 34 |
| 211922_s_at | catalase | | CAT* | | 3,70 | 391,8 ± 65,1 | | 1483,2 ± 716,5 | 591,1 ± 108,6 | | 554,2 ± 111,2 |
| 212816_s_at | cystathionine-beta-synthase | | CBS | | 4,06 | 51,4 ± 23,2 | | 200,8 ± 64,8 | 60,7 ± 13,4 | | 107 ± 25,8 |
| 1553102_a_at | coiled-coil domain containing 69 | | CCDC69* | | 11,58 | 59,6 ± 10,1 | | 1018,6 ± 907,8 | 159,7 ± 15 | | 140,6 ± 35,3 |
| 1554283_at | CCR4 carbon catabolite repression 4-like (S. cerevisiae) | | CCRN4L | | 2,70 | 29,7 ± 4,4 | | 118,2 ± 49,4 | 34,6 ± 6,2 | | 19,1 ± 6,5 |
| 214721_x_at | CDC42 effector protein (Rho GTPase binding) 4 | | CDC42EP4 | | 3,05 | 244,1 ± 61,5 | | 792,6 ± 587,6 | 218,5 ± 27,7 | | 346,5 ± 40,5 |
| 204039_at | CCAAT/enhancer binding protein (C/EBP), alpha | | CEBPA* | | 17,96 | 395,2 ± 459,7 | | 5089,6 ± 3317,1 | 431,9 ± 48,7 | | 236,8 ± 26,2 |
| 204203_at | CCAAT/enhancer binding protein (C/EBP), gamma | | CEBPG | | 3,68 | 203,4 ± 57,6 | | 945,3 ± 666,3 | 150,7 ± 11,3 | | 148 ± 23,5 |
| 209616_s_at | carboxylesterase 1 | | CES1* | | 7,41 | 116,6 ± 94,4 | | 739,2 ± 263 | 109,7 ± 15,5 | | 58 ± 16,3 |
| 205382_s_at | complement factor D (adipsin) | | CFD | | 14,81 | 455,3 ± 148,6 | | 6966,9 ± 2836,4 | 1443,6 ± 69,8 | | 840 ± 52,4 |
| 209939_x_at | CASP8 and FADD-like apoptosis regulator | | CFLAR | | 4,19 | 271,3 ± 47,9 | | 1581,2 ± 1059,6 | 285,4 ± 13,7 | | 358,3 ± 28,8 |
| 224932_at | coiled-coil-helix-coiled-coil-helix domain containing 10 | | CHCHD10 | | 10,39 | 204,2 ± 24,8 | | 2469,6 ± 2362,5 | 279,6 ± 54,7 | | 292,9 ± 62,8 |
| 222701_s_at | coiled-coil-helix-coiled-coil-helix domain containing 7 | | CHCHD7 | | 3,05 | 506,3 ± 67,8 | | 1510,7 ± 829,2 | 459,6 ± 32,1 | | 512,5 ± 69,6 |
| 213060_s_at | chitinase 3-like 2 | | CHI3L2 | | 24,07 | 179,1 ± 61,6 | | 5364,7 ± 3456,2 | 2524,7 ± 151,1 | | 338,4 ± 63,9 |
| 207993_s_at | calcium binding protein P22 | | CHP* | | 6,30 | 56,5 ± 26,9 | | 341 ± 187 | 65,4 ± 8,5 | | 102,1 ± 19,2 |
| 221675_s_at | choline phosphotransferase 1 | | CHPT1* | | 2,60 | 952,8 ± 56,9 | | 2686,2 ± 1092,4 | 1158,6 ± 25,2 | | 1201,5 ± 37,5 |
| 219398_at | cell death-inducing DFFA-like effector c | | CIDEC | | 22,45 | 75,8 ± 14,3 | | 6510 ± 6914,9 | 128,9 ± 14,3 | | 92,3 ± 26,5 |
| 200884_at | creatine kinase, brain | | CKB | | 4,29 | 146,8 ± 35 | | 494,3 ± 144,2 | 92,8 ± 25,5 | | 211,1 ± 39,8 |
| 221042_s_at | calmin (calponin-like, transmembrane) | | CLMN | | 33,51 | 16,6 ± 3,7 | | 1058,3 ± 1294,8 | 46,9 ± 3,3 | | 25,9 ± 8,4 |
| 205944_s_at | clathrin, heavy chain-like 1 | | CLTCL1 | | 2,76 | 49,2 ± 1,4 | | 125,3 ± 23,1 | 69,3 ± 13,1 | | 50,5 ± 6,3 |
| 238440_at | citrate lyase beta like | | CLYBL | | 5,53 | 31,6 ± 15,6 | | 198,3 ± 119,4 | 75,5 ± 3,3 | | 50,5 ± 11 |
| 211981_at | collagen, type IV, alpha 1 | | COL4A1 | | 4,46 | 313,4 ± 128,8 | | 1275 ± 340,7 | 323,1 ± 27,6 | | 157,8 ± 28 |
| 211025_x_at | cytochrome c oxidase subunit Vb | | COX5B | | 2,08 | 2369,1 ± 284,3 | | 5200,6 ± 2037,3 | 2352,2 ± 202,4 | | 2829 ± 147,6 |
| 206100_at | carboxypeptidase M | | CPM* | | 9,40 | 123,2 ± 56,4 | | 1193,2 ± 443,3 | 57,9 ± 2,5 | | 133,6 ± 9 |
| 209283_at | crystallin, alpha B | | CRYAB* | | 11,85 | 1070,2 ± 69,3 | | 11385 ± 2408,4 | 2532,2 ± 154,2 | | 1698,4 ± 279,5 |
| 208660_at | citrate synthase | | CS* | | 3,13 | 1212,5 ± 108,7 | | 4178 ± 3188 | 1255,5 ± 162,6 | | 1570,4 ± 295 |
| 39966_at | chondroitin sulfate proteoglycan 5 | | CSPG5 | | 3,43 | 22 ± 5,7 | | 102,4 ± 31,6 | 16,5 ± 1,6 | | 25,4 ± 2,6 |
| 204971_at | cystatin A (stefin A) | | CSTA | | 3,03 | 486,8 ± 231,2 | | 1489,1 ± 750,5 | 948,4 ± 33,7 | | 1473,7 ± 180,6 |
| 218970_s_at | cutC copper transporter homolog (E. coli) | | CUTC | | 2,21 | 232,3 ± 49,5 | | 495,1 ± 103,3 | 205 ± 19,4 | | 265,8 ± 5,2 |
| 212977_at | chemokine (C-X-C motif) receptor 7 | | CXCR7 | | 6,65 | 108,4 ± 23 | | 1006,2 ± 977,8 | 924,6 ± 47,9 | | 379 ± 54 |
| 215726_s_at | cytochrome b5 type A (microsomal) | | CYB5A | | 4,96 | 923,7 ± 172 | | 7377,6 ± 7799,1 | 1581 ± 28,5 | | 1385,3 ± 120 |
| 209389_x_at | diazepam binding inhibitor | | DBI* | | 2,76 | 2443,6 ± 313,2 | | 8461,9 ± 5749,4 | 1813,3 ± 92,9 | | 2264,1 ± 216 |
| 217973_at | dicarbonyl/L-xylulose reductase | | DCXR | | 9,77 | 294,7 ± 127,8 | | 4361,3 ± 3104,2 | 244,9 ± 39,3 | | 309,8 ± 77,8 |
| 202887_s_at | DNA-damage-inducible transcript 4 | | DDIT4 | | 8,12 | 537,1 ± 523,3 | | 4544,3 ± 3740,2 | 479,7 ± 43,5 | | 398,5 ± 33,8 |
| 202929_s_at | D-dopachrome tautomerase | | DDT | | 2,24 | 870,5 ± 15,5 | | 2099,3 ± 678,7 | 939,5 ± 82,7 | | 1086,7 ± 50,2 |
| 219402_s_at | Der1-like domain family, member 1 | | DERL1 | | 3,15 | 579,8 ± 65,3 | | 2439 ± 2574,6 | 421 ± 143,2 | | 682,9 ± 50,4 |
| 203669_s_at | diacylglycerol O-acyltransferase homolog 1 (mouse) | | DGAT1* | | 3,94 | 130,1 ± 20,2 | | 702,6 ± 710,5 | 120,7 ± 9,3 | | 166,1 ± 13,8 |
| 226064_s_at | diacylglycerol O-acyltransferase homolog 2 | | DGAT2* | | 13,72 | 117,4 ± 32,2 | | 2388,8 ± 2824,9 | 143,6 ± 12,8 | | 188,2 ± 22,8 |
| 200862_at | 24-dehydrocholesterol reductase | | DHCR24* | | 10,31 | 218 ± 24,4 | | 2355,4 ± 1228 | 163 ± 4 | | 137,9 ± 26,9 |
| 220690_s_at | dehydrogenase/reductase (SDR family) member 7B | | DHRS7B | | 2,58 | 201,4 ± 25,3 | | 575,5 ± 60,3 | 226,8 ± 29,4 | | 290,5 ± 5,9 |
| 225809_at | prostatic androgen-repressed message-1 | | DKFZP564O0823 | | 25,20 | 16,7 ± 10,7 | | 398,8 ± 237,6 | 47,3 ± 5,4 | | 46,1 ± 10,1 |
| 225195_at | DPH3, KTI11 homolog (S. cerevisiae) | | DPH3 | | 2,74 | 439,7 ± 49,3 | | 1308,4 ± 765,1 | 434,5 ± 8,4 | | 430,9 ± 49,3 |
| 219298_at | enoyl Coenzyme A hydratase domain containing 3 | | ECHDC3 | | 6,02 | 90,9 ± 47,1 | | 883,9 ± 810,8 | 152 ± 8,6 | | 89,6 ± 9,8 |
| 201135_at | enoyl Coenzyme A hydratase, short chain, 1, mitochondrial | | ECHS1* | | 2,13 | 1607,7 ± 204,4 | | 3581,8 ± 1393,1 | 1383,9 ± 48,9 | | 1707,3 ± 50 |
| 206701_x_at | endothelin receptor type B | | EDNRB* | | 12,70 | 24,4 ± 7,3 | | 745,3 ± 1040,9 | 36,9 ± 14,1 | | 13 ± 10,9 |
| 233375_at | EF-hand calcium binding domain 2 | | EFCAB2 | | 2,46 | 55,3 ± 27,6 | | 126,3 ± 32,2 | 45,2 ± 5,8 | | 63,6 ± 22,8 |
| 221539_at | eukaryotic translation initiation factor 4E binding protein 1 | | EIF4EBP1* | | 6,35 | 263,5 ± 58,3 | | 2304,7 ± 1549,8 | 248 ± 17,6 | | 227,4 ± 24,8 |
| 208770_s_at | eukaryotic translation initiation factor 4E binding protein 2 | | EIF4EBP2* | | 6,16 | 192,6 ± 10,5 | | 1477,7 ± 1211,6 | 229,8 ± 36 | | 261,7 ± 10,7 |
| 208788_at | ELOVL family member 5, elongation of long chain fatty acids (FEN1/Elo2, SUR4/Elo3-like, yeast) | | ELOVL5* | | 2,87 | 2163,1 ± 282,8 | | 6824,9 ± 3882 | 2558 ± 89,5 | | 1529,8 ± 88,8 |
| 202017_at | epoxide hydrolase 1, microsomal (xenobiotic) | | EPHX1 | | 4,67 | 269,3 ± 8,4 | | 1962,2 ± 1828,4 | 300,7 ± 31,6 | | 676,3 ± 306 |
| 203980_at | fatty acid binding protein 4, adipocyte | | FABP4* | | 452,64 | 36,5 ± 25,4 | | 17096,4 ± 6233,8 | 4804,9 ± 58,8 | | 22,3 ± 7,3 |
| 202345_s_at | fatty acid binding protein 5 (psoriasis-associated) | | FABP5* | | 18,81 | 695,4 ± 225,1 | | 13514,1 ± 5250,2 | 1672,4 ± 176,4 | | 550,5 ± 54,7 |
| 208962_s_at | fatty acid desaturase 1 | | FADS1* | | 9,26 | 358,8 ± 211,1 | | 3850,8 ± 3215,3 | 341,5 ± 11,8 | | 181,4 ± 13,4 |
| 216080_s_at | fatty acid desaturase 3 | | FADS3* | | 4,63 | 211,3 ± 66,3 | | 899,5 ± 393,3 | 255 ± 9,9 | | 197,4 ± 21,8 |
| 226448_at | family with sequence similarity 89, member A | | FAM89A | | 8,64 | 175,8 ± 35,6 | | 1809,7 ± 1011,2 | 600,6 ± 31,2 | | 220,6 ± 39,9 |
| 212218_s_at | fatty acid synthase | | FASN* | | 18,10 | 118,4 ± 20,1 | | 2553,3 ± 2775,4 | 92,8 ± 9,2 | | 112,8 ± 31 |
| 201539_s_at | four and a half LIM domains 1 | | FHL1 | | 7,24 | 199 ± 41,1 | | 2042,8 ± 1983,9 | 444,5 ± 35,7 | | 137,9 ± 19,6 |
| 204560_at | FK506 binding protein 5 | | FKBP5 | | 50,02 | 1 ± 0,2 | | 85,1 ± 41,9 | 1,4 ± 0,3 | | 1,4 ± 0,7 |
| 219316_s_at | feline leukemia virus subgroup C cellular receptor family, member 2 | | FLVCR2 | | 4,78 | 34,2 ± 17,2 | | 125,7 ± 58,9 | 29,6 ± 8,6 | | 49,5 ± 6,9 |
| 202724_s_at | forkhead box O1 | | FOXO1* | | 7,13 | 60,6 ± 9,9 | | 347,2 ± 209,9 | 122,8 ± 26,4 | | 63 ± 13,1 |
| 218665_at | frizzled homolog 4 (Drosophila) | | FZD4 | | 6,06 | 209,3 ± 61 | | 1251,6 ± 1112,8 | 231,3 ± 7,8 | | 235,5 ± 33,5 |
| 213524_s_at | G0/G1switch 2 | | G0S2 | | 48,13 | 281 ± 141,8 | | 13530,7 ± 4584,3 | 1319 ± 91,9 | | 548,8 ± 81,6 |
| 209304_x_at | growth arrest and DNA-damage-inducible, beta | | GADD45B | | 6,65 | 239,5 ± 69 | | 3028,5 ± 3941,6 | 176,1 ± 14,5 | | 214,8 ± 26,1 |
| 210005_at | phosphoribosylglycinamide formyltransferase | | GART | | 2,74 | 45,2 ± 10 | | 138,2 ± 44,7 | 56,5 ± 14,6 | | 49,5 ± 10,1 |
| 233528_s_at | GATS protein-like 3 /// TBC1 domain family, member 10A | | GATSL3 | | 3,05 | 77,1 ± 36,1 | | 253 ± 94 | 70,8 ± 9,5 | | 115,6 ± 6,4 |
| 203282_at | glucan (1,4-alpha-), branching enzyme 1 | | GBE1 | | 2,32 | 2944,3 ± 114,2 | | 7056,5 ± 2761,2 | 3123,5 ± 30,9 | | 3313,2 ± 52,1 |
| 203500_at | glutaryl-Coenzyme A dehydrogenase | | GCDH* | | 3,17 | 103,5 ± 18,1 | | 310,5 ± 88,8 | 94,1 ± 19,5 | | 125,8 ± 19,6 |
| 204224_s_at | GTP cyclohydrolase 1 | | GCH1* | | 2,94 | 40,6 ± 3,7 | | 130,3 ± 16,9 | 48,9 ± 8,9 | | 24,7 ± 1,6 |
| 204867_at | GTP cyclohydrolase I feedback regulator | | GCHFR | | 3,56 | 147,3 ± 40 | | 613,4 ± 176 | 162 ± 16,4 | | 248 ± 40,8 |
| 213133_s_at | glycine cleavage system protein H (aminomethyl carrier) | | GCSH | | 2,74 | 698,2 ± 100,2 | | 2003,4 ± 688,2 | 713,6 ± 16 | | 496,6 ± 33,1 |
| 205498_at | growth hormone receptor | | GHR* | | 4,63 | 377,7 ± 74 | | 2175 ± 1608 | 615,7 ± 62 | | 376,8 ± 22,6 |
| 225602_at | GLI pathogenesis-related 2 | | GLIPR2* | | 3,46 | 202,8 ± 42,4 | | 561,9 ± 118,1 | 228,7 ± 9,5 | | 197,2 ± 10,7 |
| 219933_at | glutaredoxin 2 | | GLRX2 | | 2,68 | 705,6 ± 166,1 | | 1973,4 ± 984 | 715,3 ± 53,4 | | 724,1 ± 47,1 |
| 200648_s_at | glutamate-ammonia ligase (glutamine synthetase) | | GLUL | | 15,51 | 135,6 ± 17,6 | | 3265 ± 2072,6 | 100,6 ± 10,1 | | 167,8 ± 20,1 |
| 210009_s_at | golgi SNAP receptor complex member 2 | | GOSR2 | | 3,48 | 110,2 ± 44,3 | | 375,1 ± 250,2 | 91,7 ± 11,8 | | 101,2 ± 6,9 |
| 202756_s_at | glypican 1 | | GPC1* | | 3,82 | 329,3 ± 41,4 | | 1206,3 ± 699,7 | 462,2 ± 85,5 | | 382,1 ± 19 |
| 204983_s_at | glypican 4 | | GPC4* | | 3,59 | 185,9 ± 108,7 | | 618,5 ± 183,2 | 368,3 ± 4,8 | | 154,1 ± 12,8 |
| 213706_at | glycerol-3-phosphate dehydrogenase 1 (soluble) | | GPD1* | | 181,02 | 11,3 ± 10,3 | | 3191,6 ± 4233,5 | 37,2 ± 1,9 | | 21,4 ± 6,1 |
| 224839_s_at | glutamic pyruvate transaminase (alanine aminotransferase) 2 | | GPT2 | | 9,55 | 53,7 ± 4,2 | | 545,3 ± 423,3 | 42,8 ± 6,4 | | 79,4 ± 12,1 |
| 214091_s_at | glutathione peroxidase 3 (plasma) | | GPX3 | | 17,28 | 134,3 ± 32,7 | | 3915,1 ± 4416,5 | 398,5 ± 36,3 | | 172,3 ± 62,6 |
| 212432_at | GrpE-like 1, mitochondrial (E. coli) | | GRPEL1 | | 2,48 | 294,3 ± 16,7 | | 847,5 ± 302 | 208,6 ± 22,4 | | 307,6 ± 55,6 |
| 214040_s_at | gelsolin (amyloidosis, Finnish type) | | GSN | | 2,74 | 225,9 ± 25,8 | | 627,6 ± 195 | 230,3 ± 13,8 | | 383,4 ± 52,9 |
| 225609_at | glutathione reductase | | GSR | | 2,39 | 317,5 ± 22,5 | | 972,1 ± 610,6 | 472,5 ± 16,6 | | 303,4 ± 28,1 |
| 201673_s_at | glycogen synthase 1 (muscle) | | GYS1* | | 3,54 | 272,4 ± 66 | | 1112,8 ± 774,9 | 130,5 ± 14,6 | | 372,1 ± 47,8 |
| 223211_at | 2-hydroxyacyl-CoA lyase 1 | | HACL1* | | 2,52 | 205 ± 33,9 | | 513,1 ± 160,5 | 144,7 ± 9,4 | | 216,6 ± 49,9 |
| 211569_s_at | hydroxyacyl-Coenzyme A dehydrogenase | | HADH* | | 10,48 | 344,9 ± 59,6 | | 5444,5 ± 6064,9 | 518,3 ± 46,2 | | 406,1 ± 29,2 |
| 208629_s_at | hydroxyacyl-Coenzyme A dehydrogenase | | HADHA* | | 2,26 | 867,5 ± 51,5 | | 1964,7 ± 554,3 | 1255,1 ± 102,8 | | 1415,6 ± 115,6 |
| 203430_at | heme binding protein 2 | | HEBP2 | | 2,19 | 2434,9 ± 150 | | 6390,7 ± 4330,8 | 1988,3 ± 21,9 | | 2474,8 ± 117,3 |
| 209398_at | histone cluster 1, H1c | | HIST1H1C | | 2,99 | 444,4 ± 169,7 | | 1307,1 ± 465 | 522,5 ± 26,7 | | 837,1 ± 71,2 |
| 206697_s_at | haptoglobin | | HP | | 145,91 | 18,2 ± 14,3 | | 5541,6 ± 4237,3 | 969,9 ± 35,3 | | 64,4 ± 21 |
| 208470_s_at | haptoglobin /// haptoglobin-related protein | | HP /// HPR | | 330,08 | 11,3 ± 8,3 | | 5003,2 ± 4040,5 | 948,5 ± 70,9 | | 36,8 ± 17,2 |
| 203914_x_at | hydroxyprostaglandin dehydrogenase 15-(NAD) | | HPGD* | | 10,48 | 7,5 ± 7,1 | | 57,4 ± 16,8 | 11,5 ± 4,8 | | 9,2 ± 8,2 |
| 219697_at | heparan sulfate (glucosamine) 3-O-sulfotransferase 2 | | HS3ST2 | | 10,31 | 26,7 ± 13,1 | | 348,4 ± 275,9 | 33,1 ± 10,7 | | 30,8 ± 5,2 |
| 205404_at | hydroxysteroid (11-beta) dehydrogenase 1 | | HSD11B1* | | 29,63 | 636,9 ± 633,8 | | 13757 ± 5352,3 | 1622,6 ± 142,3 | | 1798,4 ± 368,5 |
| 202282_at | hydroxysteroid (17-beta) dehydrogenase 10 | | HSD17B10* | | 2,48 | 722,8 ± 96 | | 1890,6 ± 744,6 | 565,8 ± 29 | | 769,7 ± 40,5 |
| 211538_s_at | heat shock 70kDa protein 2 | | HSPA2 | | 4,06 | 101,5 ± 16,5 | | 352,6 ± 142,3 | 139,3 ± 20,8 | | 153 ± 29,3 |
| 204949_at | intercellular adhesion molecule 3 | | ICAM3 | | 2,64 | 205,2 ± 14,7 | | 618 ± 236,4 | 179 ± 18,4 | | 372,9 ± 26,4 |
| 1555037_a_at | isocitrate dehydrogenase 1 (NADP+), soluble | | IDH1* | | 3,59 | 2091,8 ± 130,3 | | 7820,7 ± 3222,5 | 2415,1 ± 52,1 | | 2222,9 ± 170 |
| 210046_s_at | isocitrate dehydrogenase 2 (NADP+), mitochondrial | | IDH2* | | 3,73 | 410 ± 110 | | 1572,4 ± 285,9 | 371,9 ± 38,3 | | 213,2 ± 9,6 |
| 205258_at | inhibin, beta B | | INHBB | | 24,25 | 13,4 ± 6,6 | | 296,9 ± 184,9 | 38,5 ± 9,2 | | 29 ± 21,3 |
| 209184_s_at | insulin receptor substrate 2 | | IRS2* | | 6,40 | 289,9 ± 112 | | 2277,2 ± 2359 | 311,4 ± 21,5 | | 282,7 ± 33,4 |
| 218170_at | isochorismatase domain containing 1 | | ISOC1 | | 4,39 | 219,7 ± 16,6 | | 1208,4 ± 614,3 | 294,1 ± 22,5 | | 228 ± 24,6 |
| 216331_at | integrin, alpha 7 | | ITGA7 | | 7,82 | 125,8 ± 31,5 | | 2009,3 ± 1800,4 | 438,1 ± 46,1 | | 574,9 ± 37,9 |
| 210740_s_at | inositol 1,3,4-triphosphate 5/6 kinase | | ITPK1 | | 3,13 | 318,6 ± 63 | | 965,1 ± 319,2 | 383,8 ± 20 | | 451,3 ± 26,6 |
| 213005_s_at | KN motif and ankyrin repeat domains 1 | | KANK1 | | 2,44 | 670,3 ± 50,4 | | 1976,1 ± 1404,8 | 802 ± 38,3 | | 1310,4 ± 44,4 |
| 231015_at | Kruppel-like factor 15 | | KLF15 | | 15,63 | 132,7 ± 75,1 | | 2236,3 ± 2148 | 248,2 ± 15 | | 180,5 ± 29,7 |
| 235463_s_at | LAG1 homolog, ceramide synthase 6 | | LASS6* | | 6,16 | 61,2 ± 20,7 | | 569,8 ± 654,8 | 72,2 ± 1,7 | | 60,4 ± 4,8 |
| 231715_s_at | left-right determination factor 1 /// pyrroline-5-carboxylate reductase family, member 2 | | LEFTY1 /// PYCR2 | | 2,03 | 194,6 ± 65 | | 361,5 ± 8,7 | 192 ± 14,7 | | 224,6 ± 27,1 |
| 223828_s_at | lectin, galactoside-binding, soluble, 12 | | LGALS12 | | 28,29 | 38,8 ± 29 | | 2463,9 ± 3463 | 74 ± 13,6 | | 70,2 ± 17,6 |
| 206440_at | lin-7 homolog A (C. elegans) | | LIN7A | | 2,81 | 80,6 ± 10 | | 214,6 ± 145,7 | 96,3 ± 12,4 | | 60,6 ± 9,7 |
| 208186_s_at | lipase, hormone-sensitive | | LIPE* | | 17,28 | 29,5 ± 19,5 | | 637 ± 900,4 | 26,3 ± 26,9 | | 49,6 ± 13,2 |
| 218574_s_at | LIM and cysteine-rich domains 1 | | LMCD1 | | 4,67 | 113,2 ± 31,8 | | 520,9 ± 273,6 | 227,8 ± 35,5 | | 118,2 ± 5 |
| 209205_s_at | LIM domain only 4 | | LMO4 | | 2,70 | 789,1 ± 112,3 | | 2465,1 ± 1362,6 | 938,2 ± 74,7 | | 613,3 ± 65 |
| 236728_at | leucyl/cystinyl aminopeptidase | | LNPEP | | 8,06 | 42,2 ± 8,7 | | 303,9 ± 165,6 | 61,7 ± 2,5 | | 41,9 ± 11,3 |
| 242931_at | LON peptidase N-terminal domain and ring finger 3 | | LONRF3 | | 7,13 | 14,5 ± 1,7 | | 124 ± 93,5 | 41,2 ± 7,6 | | 21,8 ± 4,4 |
| 203549_s_at | lipoprotein lipase | | LPL* | | 212,80 | 16,2 ± 9,7 | | 5303,5 ± 5164,5 | 933,6 ± 37,1 | | 16,2 ± 14,2 |
| 204388_s_at | monoamine oxidase A | | MAOA | | 40,94 | 34 ± 19,1 | | 1563,4 ± 974,6 | 160,3 ± 5,3 | | 69,5 ± 16,9 |
| 204041_at | monoamine oxidase B | | MAOB | | 4,74 | 63,8 ± 23,9 | | 403,4 ± 265,2 | 85,4 ± 3,1 | | 188,6 ± 61,2 |
| 205698_s_at | mitogen-activated protein kinase kinase 6 | | MAP2K6 | | 3,62 | 69,5 ± 25 | | 226,8 ± 115,8 | 89,4 ± 13,8 | | 93,9 ± 6,2 |
| 217943_s_at | MAP7 domain containing 1 | | MAP7D1 | | 2,30 | 611,6 ± 32,4 | | 1404 ± 717,1 | 406 ± 8,4 | | 697,6 ± 76,3 |
| 201475_x_at | methionyl-tRNA synthetase | | MARS | | 3,27 | 416,7 ± 47,6 | | 1641,7 ± 1157,6 | 338,6 ± 21,7 | | 375,6 ± 49,8 |
| 210869_s_at | melanoma cell adhesion molecule | | MCAM | | 16,12 | 92,8 ± 34,4 | | 2231,1 ± 2182,7 | 160,6 ± 20,9 | | 28,2 ± 5,9 |
| 232092_at | mitochondrial carrier triple repeat 1 | | MCART1 | | 3,73 | 63,8 ± 12 | | 262,2 ± 115,2 | 52,2 ± 20,1 | | 63,3 ± 6,5 |
| 204059_s_at | malic enzyme 1, NADP(+)-dependent, cytosolic | | ME1* | | 5,04 | 1011,9 ± 112,2 | | 6135,6 ± 3789,9 | 965,6 ± 19,5 | | 1023,1 ± 32,3 |
| 224476_s_at | mesoderm posterior 1 homolog (mouse) | | MESP1 | | 10,97 | 46,4 ± 7,7 | | 703,5 ± 589,4 | 73,5 ± 12,3 | | 102 ± 6,3 |
| 210605_s_at | milk fat globule-EGF factor 8 protein | | MFGE8* | | 2,41 | 164,7 ± 35,9 | | 420,7 ± 36,4 | 205,2 ± 35,4 | | 211,8 ± 25,4 |
| 1565162_s_at | microsomal glutathione S-transferase 1 | | MGST1* | | 2,37 | 4221,6 ± 875,9 | | 10431 ± 4827,7 | 5153,6 ± 244,8 | | 3903,9 ± 135,7 |
| 223199_at | MAP kinase interacting serine/threonine kinase 2 | | MKNK2 | | 9,93 | 105,7 ± 26,9 | | 1590,3 ± 1736,9 | 89,3 ± 27 | | 161,7 ± 23,5 |
| 244523_at | monocyte to macrophage differentiation-associated | | MMD | | 4,09 | 56,9 ± 28,6 | | 317,1 ± 108,6 | 129,5 ± 26,9 | | 55,7 ± 4,9 |
| 218865_at | MOCO sulphurase C-terminal domain containing 1 | | MOSC1 | | 54,03 | 28,6 ± 18,4 | | 1417,4 ± 694 | 71,9 ± 13,9 | | 10,9 ± 1,3 |
| 219527_at | MOCO sulphurase C-terminal domain containing 2 | | MOSC2 | | 3,65 | 38 ± 1,3 | | 181,9 ± 105,9 | 74,3 ± 13,3 | | 58,8 ± 13 |
| 210087_s_at | myelin protein zero-like 1 | | MPZL1 | | 3,56 | 187,5 ± 54,1 | | 708,3 ± 517,8 | 137,6 ± 16,3 | | 190,2 ± 6,9 |
| 1555740_a_at | melanocortin 2 receptor accessory protein | | MRAP | | 65,50 | 22,7 ± 21,3 | | 2838,7 ± 3468,1 | 143,4 ± 26,6 | | 43,7 ± 14,8 |
| 218890_x_at | mitochondrial ribosomal protein L35 | | MRPL35 | | 2,37 | 333,9 ± 52 | | 861,4 ± 275,4 | 335,4 ± 17,8 | | 352 ± 57,6 |
| 203800_s_at | mitochondrial ribosomal protein S14 | | MRPS14 | | 2,13 | 716,4 ± 66,4 | | 1671,9 ± 516,8 | 659,5 ± 109,6 | | 746,1 ± 96,4 |
| 217932_at | mitochondrial ribosomal protein S7 | | MRPS7 | | 2,16 | 498,9 ± 79,8 | | 1058,7 ± 318,6 | 379,7 ± 37,1 | | 445,4 ± 19,3 |
| 208918_s_at | NAD kinase | | NADK | | 2,37 | 331,5 ± 46,8 | | 853,6 ± 433,6 | 261,1 ± 37,3 | | 436,3 ± 45,1 |
| 228523_at | nanos homolog 1 (Drosophila) | | NANOS1 | | 11,23 | 64,6 ± 17,6 | | 935,2 ± 934,9 | 71,1 ± 16,4 | | 75,6 ± 11 |
| 226616_s_at | NADH dehydrogenase (ubiquinone) flavoprotein 3, 10kDa | | NDUFV3 | | 3,01 | 858,4 ± 127,3 | | 3247,5 ± 2231,8 | 835,8 ± 12,1 | | 1087,2 ± 106,5 |
| 203574_at | nuclear factor, interleukin 3 regulated | | NFIL3 | | 2,83 | 703,1 ± 104 | | 2335,5 ± 1665 | 1367,3 ± 79 | | 560 ± 23,3 |
| 201502_s_at | nuclear factor of kappa light polypeptide gene enhancer in B-cells inhibitor, alpha | | NFKBIA* | | 3,79 | 262,5 ± 42,6 | | 1119,5 ± 501 | 367,7 ± 135,3 | | 518,7 ± 86,2 |
| 205204_at | neuromedin B | | NMB | | 3,65 | 762,5 ± 92,5 | | 3338,2 ± 2164,4 | 2697,7 ± 128,2 | | 952 ± 48,4 |
| 213040_s_at | neuronal pentraxin receptor | | NPTXR | | 5,83 | 45 ± 16,2 | | 215,7 ± 80,5 | 88,3 ± 9,3 | | 127,9 ± 11,7 |
| 203814_s_at | NAD(P)H dehydrogenase, quinone 2 | | NQO2 | | 4,32 | 697,5 ± 263,2 | | 3256,5 ± 1742,6 | 750,9 ± 23,4 | | 1131,3 ± 92 |
| 203920_at | nuclear receptor subfamily 1, group H, member 3 | | NR1H3* | | 16,63 | 130,6 ± 30,9 | | 3181,5 ± 3922,7 | 243,7 ± 18,1 | | 211,8 ± 9,2 |
| 204105_s_at | neuronal cell adhesion molecule | | NRCAM | | 10,56 | 19 ± 4,8 | | 380,4 ± 391,3 | 36,8 ± 12,4 | | 17,1 ± 6,9 |
| 209279_s_at | NAD(P) dependent steroid dehydrogenase-like | | NSDHL* | | 4,42 | 155 ± 10,5 | | 923,5 ± 748,6 | 133,2 ± 14,6 | | 170,4 ± 17,4 |
| 217499_x_at | olfactory receptor, family 7, subfamily E, member 37 pseudogene | | OR7E37P | | 2,21 | 297,1 ± 90 | | 689,6 ± 209,2 | 275,7 ± 19,1 | | 240,5 ± 3 |
| 222454_s_at | parvin, alpha | | PARVA | | 2,32 | 352,1 ± 70,7 | | 790 ± 423,7 | 345,2 ± 2,5 | | 362,7 ± 26,4 |
| 208383_s_at | phosphoenolpyruvate carboxykinase 1 (soluble) | | PCK1* | | 445,72 | 3 ± 1,8 | | 2147 ± 2206,2 | 19 ± 2,3 | | 2,1 ± 1,3 |
| 205559_s_at | proprotein convertase subtilisin/kexin type 5 | | PCSK5 | | 2,66 | 183,7 ± 55,3 | | 482,7 ± 167,5 | 236,7 ± 28,2 | | 173 ± 10,7 |
| 209577_at | phosphate cytidylyltransferase 2, ethanolamine | | PCYT2* | | 4,74 | 124,4 ± 19 | | 622,9 ± 568,5 | 124,1 ± 16,7 | | 119,4 ± 14,2 |
| 222317_at | phosphodiesterase 3B, cGMP-inhibited | | PDE3B* | | 17,28 | 13,5 ± 8,5 | | 349,6 ± 429,3 | 16,6 ± 4 | | 10,7 ± 4,1 |
| 213228_at | phosphodiesterase 8B | | PDE8B | | 15,76 | 14,1 ± 9,3 | | 235,1 ± 237,9 | 19,6 ± 1,8 | | 18,4 ± 4,9 |
| 1555864_s_at | pyruvate dehydrogenase (lipoamide) alpha 1 | | PDHA1 | | 2,42 | 754 ± 94,4 | | 2043,8 ± 1035,4 | 779,4 ± 77,5 | | 709,4 ± 41,5 |
| 208690_s_at | PDZ and LIM domain 1 | | PDLIM1 | | 4,78 | 816,2 ± 293,8 | | 3917,8 ± 2714,2 | 2746 ± 71,7 | | 1188,1 ± 104,8 |
| 232861_at | pyruvate dehyrogenase phosphatase catalytic subunit 2 | | PDP2 | | 3,17 | 48,6 ± 21,5 | | 130,5 ± 66 | 66,8 ± 16,7 | | 89,4 ± 18 |
| 221142_s_at | peroxisomal trans-2-enoyl-CoA reductase | | PECR* | | 9,93 | 78,1 ± 7,2 | | 695,9 ± 244,5 | 118,2 ± 13,2 | | 79,6 ± 14,7 |
| 207621_s_at | phosphatidylethanolamine N-methyltransferase | | PEMT* | | 2,48 | 227 ± 19,9 | | 658 ± 464,9 | 197,8 ± 22,7 | | 248,9 ± 20,6 |
| 201706_s_at | peroxisomal biogenesis factor 19 | | PEX19* | | 3,79 | 204,9 ± 15,2 | | 741,9 ± 577 | 268,2 ± 8,2 | | 311,2 ± 50,4 |
| 222622_at | phosphoglycolate phosphatase | | PGP | | 3,17 | 148,4 ± 35,7 | | 497,9 ± 223,2 | 113,8 ± 10,6 | | 157,6 ± 11,9 |
| 220041_at | phosphatidylinositol glycan anchor biosynthesis, class Z | | PIGZ* | | 3,13 | 97,3 ± 25,9 | | 296,6 ± 134,1 | 170,5 ± 12,9 | | 161,8 ± 3,7 |
| 207469_s_at | pirin (iron-binding nuclear protein) | | PIR | | 3,03 | 263,3 ± 47,5 | | 836,7 ± 301,7 | 256,8 ± 12,4 | | 184,1 ± 39,4 |
| 209581_at | phospholipase A2, group XVI | | PLA2G16* | | 6,60 | 446,3 ± 119,9 | | 3748,8 ± 3218,7 | 823,1 ± 58,5 | | 473,4 ± 44 |
| 225726_s_at | pleckstrin homology domain containing, family H member 1 | | PLEKHH1 | | 4,96 | 27,9 ± 2,6 | | 166,7 ± 81,7 | 43,1 ± 10,9 | | 36,9 ± 3,3 |
| 205913_at | perilipin 1 | | PLIN1* | | 729,68 | 4,8 ± 2,7 | | 5584,4 ± 5352,9 | 525,2 ± 79,9 | | 13,7 ± 8,9 |
| 228409_at | perilipin 4 | | PLIN4 | | 320,07 | 4,3 ± 3,7 | | 2190,7 ± 2804,5 | 69,8 ± 17,5 | | 10,4 ± 3,1 |
| 203622_s_at | partner of NOB1 homolog (S. cerevisiae) | | PNO1 | | 2,35 | 410,8 ± 29,3 | | 927 ± 404,3 | 299,7 ± 27,2 | | 351,1 ± 28,4 |
| 204839_at | processing of precursor 5, ribonuclease P/MRP subunit | | POP5 | | 2,37 | 931,9 ± 91,4 | | 2237,1 ± 332 | 987,7 ± 55 | | 1136,2 ± 153,1 |
| 209147_s_at | phosphatidic acid phosphatase type 2A | | PPAP2A* | | 2,72 | 1013,6 ± 254,5 | | 2705 ± 1022,1 | 3059,5 ± 133,7 | | 938,7 ± 122,7 |
| 208510_s_at | peroxisome proliferator-activated receptor gamma | | PPARG* | | 9,62 | 221,9 ± 47,2 | | 2143,9 ± 1010,3 | 501,4 ± 15,5 | | 292,5 ± 44,6 |
| 218010_x_at | pancreatic progenitor cell differentiation and proliferation factor homolog (zebrafish) | | PPDPF | | 2,72 | 105 ± 22,4 | | 256 ± 67,2 | 136,7 ± 32,4 | | 99,9 ± 64,9 |
| 201489_at | peptidylprolyl isomerase F | | PPIF | | 3,88 | 614,5 ± 87,7 | | 2685,4 ± 1487,9 | 817,5 ± 61,7 | | 618,7 ± 149,4 |
| 205478_at | protein phosphatase 1, regulatory (inhibitor) subunit 1A | | PPP1R1A | | 68,07 | 6,5 ± 4,7 | | 1151,2 ± 1478,3 | 48,8 ± 15,5 | | 5,3 ± 4,6 |
| 200844_s_at | peroxiredoxin 6 | | PRDX6* | | 2,68 | 2920,6 ± 129,7 | | 8849 ± 3946,5 | 2588,3 ± 82,6 | | 3036 ± 195 |
| 203680_at | protein kinase, cAMP-dependent, regulatory, type II, beta | | PRKAR2B* | | 13,20 | 217,6 ± 133,1 | | 3563,4 ± 3302,9 | 533,7 ± 33,9 | | 208,8 ± 18,3 |
| 209385_s_at | proline synthetase co-transcribed homolog (bacterial) | | PROSC | | 2,37 | 324,3 ± 41,9 | | 782,6 ± 88,6 | 376,9 ± 34,6 | | 420,6 ± 30 |
| 223062_s_at | phosphoserine aminotransferase 1 | | PSAT1 | | 13,20 | 245,9 ± 116,4 | | 3977,1 ± 3762,9 | 153,4 ± 13 | | 129,9 ± 26,9 |
| 208805_at | proteasome (prosome, macropain) subunit, alpha type, 6 | | PSMA6 | | 2,32 | 3894,7 ± 451,3 | | 9186,4 ± 3342,5 | 3483,8 ± 67,6 | | 3674,8 ± 597,5 |
| 209853_s_at | proteasome (prosome, macropain) activator subunit 3 (PA28 gamma; Ki) | | PSME3 | | 3,17 | 220,7 ± 43,1 | | 643,2 ± 300,1 | 191,1 ± 36,6 | | 233,5 ± 12,9 |
| 211600_at | protein tyrosine phosphatase, receptor type, O | | PTPRO | | 2,46 | 1003,4 ± 176,7 | | 2629,1 ± 1417,7 | 1260 ± 100,2 | | 2574,3 ± 479,7 |
| 226436_at | Ras association (RalGDS/AF-6) domain family member 4 | | RASSF4 | | 7,76 | 74,4 ± 24,1 | | 861,3 ± 680 | 217,3 ± 29,7 | | 146,3 ± 16,4 |
| 219140_s_at | retinol binding protein 4, plasma | | RBP4* | | 25,01 | 349,9 ± 223,5 | | 8520,7 ± 6617,9 | 1815,9 ± 24,5 | | 227 ± 14,9 |
| 208872_s_at | receptor accessory protein 5 | | REEP5 | | 2,14 | 1717,8 ± 295,9 | | 3810,3 ± 291,6 | 1661,9 ± 64,9 | | 2110,6 ± 157,1 |
| 1566472_s_at | retinol saturase (all-trans-retinol 13,14-reductase) | | RETSAT | | 11,23 | 137,1 ± 27,1 | | 1841,6 ± 1619,9 | 137 ± 13,7 | | 205,7 ± 36,5 |
| 202388_at | regulator of G-protein signaling 2, 24kDa | | RGS2* | | 5,57 | 231,5 ± 71,1 | | 2410,7 ± 3196,1 | 197,6 ± 29,8 | | 313,8 ± 20,1 |
| 212099_at | ras homolog gene family, member B | | RHOB* | | 7,70 | 228,9 ± 124,2 | | 1818,1 ± 686,9 | 252,8 ± 18,7 | | 248,8 ± 108,9 |
| 223168_at | ras homolog gene family, member U | | RHOU* | | 2,89 | 66,2 ± 19 | | 171,8 ± 49,4 | 72,9 ± 5,3 | | 45,9 ± 9,9 |
| 227657_at | ring finger protein 150 | | RNF150 | | 7,41 | 51,2 ± 22 | | 439,4 ± 313,8 | 128,7 ± 16,7 | | 59,5 ± 8,2 |
| 224564_s_at | reticulon 3 | | RTN3 | | 2,87 | 965,7 ± 96 | | 2855,5 ± 1525,2 | 867,1 ± 71,1 | | 1437,7 ± 194 |
| 228176_at | sphingosine-1-phosphate receptor 3 | | S1PR3* | | 5,20 | 258,7 ± 61,3 | | 1407,3 ± 768,4 | 315,1 ± 2,4 | | 222,3 ± 16 |
| 201543_s_at | SAR1 homolog A (S. cerevisiae) | | SAR1A | | 3,20 | 649,5 ± 37,2 | | 2144,4 ± 976,4 | 654,6 ± 62,7 | | 606,8 ± 29,5 |
| 213988_s_at | spermidine/spermine N1-acetyltransferase 1 | | SAT1 | | 3,97 | 881,1 ± 173,9 | | 3525,3 ± 1061,3 | 1025,8 ± 46,3 | | 942,8 ± 105,8 |
| 218143_s_at | secretory carrier membrane protein 2 | | SCAMP2 | | 2,48 | 323 ± 3,8 | | 773,2 ± 136,4 | 302,9 ± 23,1 | | 464,9 ± 65,2 |
| 211708_s_at | stearoyl-CoA desaturase (delta-9-desaturase) | | SCD* | | 327,55 | 14 ± 8,8 | | 7022,4 ± 5187,5 | 53,1 ± 9,5 | | 7,1 ± 4 |
| 212158_at | syndecan 2 | | SDC2 | | 5,28 | 584,8 ± 300 | | 3260,5 ± 524,5 | 631 ± 40,9 | | 320 ± 27,7 |
| 219689_at | sema domain, immunoglobulin domain (Ig), short basic domain, secreted, (semaphorin) 3G | | SEMA3G | | 100,04 | 3,8 ± 2,7 | | 548,4 ± 543,7 | 18,2 ± 13,4 | | 4 ± 0,9 |
| 202283_at | serpin peptidase inhibitor, clade F (alpha-2 antiplasmin, pigment epithelium derived factor), member 1 | | SERPINF1 | | 2,60 | 4541,6 ± 1613,5 | | 12679 ± 5146 | 5529,4 ± 46,9 | | 8225,5 ± 95,7 |
| 235683_at | sestrin 3 | | SESN3 | | 4,03 | 58,1 ± 28,6 | | 279,8 ± 171 | 97,7 ± 25,3 | | 65,2 ± 20 |
| 225143_at | sideroflexin 4 | | SFXN4 | | 2,96 | 199,8 ± 24,3 | | 606,4 ± 329,3 | 199,2 ± 17,1 | | 243,6 ± 3,9 |
| 207302_at | sarcoglycan, gamma (35kDa dystrophin-associated glycoprotein) | | SGCG | | 25,01 | 1,9 ± 0,8 | | 79,6 ± 39,8 | 4,4 ± 3,9 | | 2,7 ± 0,9 |
| 210101_x_at | SH3-domain GRB2-like endophilin B1 | | SH3GLB1* | | 2,02 | 1337,8 ± 107,2 | | 2657,8 ± 676,3 | 1490,9 ± 27,4 | | 1261 ± 54,4 |
| 223430_at | salt-inducible kinase 2 | | SIK2* | | 4,92 | 180,2 ± 18,1 | | 962 ± 462,1 | 331 ± 26,9 | | 151,3 ± 5,7 |
| 209681_at | solute carrier family 19 (thiamine transporter), member 2 | | SLC19A2* | | 5,40 | 168,7 ± 26,9 | | 1409 ± 1452,6 | 242,6 ± 38,7 | | 145,8 ± 19,9 |
| 239345_at | solute carrier family 19, member 3 | | SLC19A3 | | 18,38 | 17,2 ± 7,6 | | 300,5 ± 212,2 | 150 ± 8,4 | | 27,5 ± 6,4 |
| 212810_s_at | solute carrier family 1 (glutamate/neutral amino acid transporter), member 4 | | SLC1A4 | | 4,63 | 70,7 ± 15,9 | | 331,4 ± 220,5 | 64 ± 9,9 | | 52,4 ± 9,3 |
| 210010_s_at | solute carrier family 25 (mitochondrial carrier; citrate transporter), member 1 | | SLC25A1 | | 5,44 | 301,6 ± 50,8 | | 2210,5 ± 2229,6 | 283,7 ± 13,4 | | 384,7 ± 72 |
| 202825_at | solute carrier family 25 (mitochondrial carrier; adenine nucleotide translocator), member 4 | | SLC25A4 | | 2,37 | 356,8 ± 48,1 | | 859,9 ± 165,9 | 361,1 ± 30,6 | | 633,5 ± 105,6 |
| 221572_s_at | solute carrier family 26, member 6 | | SLC26A6 | | 2,58 | 46,5 ± 9,2 | | 119,7 ± 10,5 | 49,1 ± 4,2 | | 58,5 ± 26 |
| 220091_at | solute carrier family 2 (facilitated glucose transporter), member 6 | | SLC2A6 | | 2,79 | 103 ± 22,7 | | 418 ± 341,9 | 64,7 ± 10,2 | | 122,1 ± 18,5 |
| 203971_at | solute carrier family 31 (copper transporters), member 1 | | SLC31A1 | | 3,22 | 301 ± 98 | | 1124,9 ± 861,8 | 265,8 ± 31,9 | | 429,9 ± 11 |
| 213119_at | solute carrier family 36 (proton/amino acid symporter), member 1 | | SLC36A1 | | 2,16 | 117,9 ± 11,3 | | 231,6 ± 34,3 | 121,2 ± 30,2 | | 129,4 ± 5,6 |
| 228486_at | solute carrier family 44, member 1 | | SLC44A1 | | 6,65 | 78,3 ± 18,5 | | 832,5 ± 1112,1 | 41,8 ± 13,3 | | 231,2 ± 22,8 |
| 224609_at | solute carrier family 44, member 2 | | SLC44A2 | | 3,82 | 124,9 ± 30,4 | | 564,6 ± 366 | 158,5 ± 11,1 | | 247,1 ± 4,4 |
| 222513_s_at | sorbin and SH3 domain containing 1 | | SORBS1* | | 29,18 | 8,9 ± 5,7 | | 337,1 ± 219,2 | 47,3 ± 4,8 | | 7,7 ± 2,9 |
| 212807_s_at | sortilin 1 | | SORT1* | | 12,03 | 113,6 ± 29,3 | | 1601 ± 885,1 | 308,5 ± 39,6 | | 121 ± 9 |
| 213993_at | spondin 1, extracellular matrix protein | | SPON1 | | 13,00 | 12,6 ± 11,6 | | 120,1 ± 49 | 8,1 ± 12 | | 19,5 ± 8,6 |
| 203458_at | sepiapterin reductase (7,8-dihydrobiopterin:NADP+ oxidoreductase) | | SPR | | 2,54 | 251,7 ± 25,7 | | 655,3 ± 203,8 | 313,2 ± 25,9 | | 362,8 ± 32,8 |
| 222750_s_at | steroid 5 alpha-reductase 3 | | SRD5A3* | | 3,82 | 400,3 ± 45,4 | | 1508,3 ± 532,5 | 406,9 ± 22 | | 552,8 ± 7,7 |
| 219205_at | serine racemase | | SRR* | | 2,08 | 174,7 ± 28,3 | | 348,4 ± 78 | 267,2 ± 12 | | 199,6 ± 35,5 |
| 222411_s_at | signal sequence receptor, gamma (translocon-associated protein gamma) | | SSR3 | | 2,23 | 2148,7 ± 704,9 | | 4429,1 ± 1052,5 | 1687,2 ± 54,7 | | 1962,3 ± 53,7 |
| 225033_at | ST3 beta-galactoside alpha-2,3-sialyltransferase 1 | | ST3GAL1 | | 4,78 | 88,7 ± 8,9 | | 560,7 ± 542,2 | 94,5 ± 10,2 | | 108,4 ± 14,5 |
| 200028_s_at | StAR-related lipid transfer (START) domain containing 7 | | STARD7 | | 2,03 | 1407,8 ± 62 | | 3079,6 ± 1159,6 | 1399,7 ± 94,5 | | 2005,5 ± 41,8 |
| 203010_at | signal transducer and activator of transcription 5A | | STAT5A* | | 4,32 | 138,5 ± 49,2 | | 594 ± 285,1 | 210,8 ± 26,7 | | 201,5 ± 26,3 |
| 203986_at | starch binding domain 1 | | STBD1 | | 10,16 | 36,1 ± 21,7 | | 319,4 ± 118,6 | 66,6 ± 8,5 | | 57,2 ± 5,3 |
| 201060_x_at | stomatin | | STOM* | | 7,70 | 756,3 ± 308,5 | | 5992,5 ± 2026,6 | 2207,4 ± 165,9 | | 858,4 ± 30,5 |
| 204295_at | surfeit 1 /// vacuolar protein sorting 37 homolog A (S. cerevisiae) | | SURF1 /// VPS37A | | 3,68 | 600,2 ± 221,2 | | 2315,4 ± 1028,3 | 982,3 ± 179 | | 1173,5 ± 17,7 |
| 210247_at | synapsin II | | SYN2 | | 14,25 | 32,8 ± 13,8 | | 594,3 ± 609 | 49 ± 5,3 | | 38,5 ± 2,7 |
| 201463_s_at | transaldolase 1 | | TALDO1 | | 2,70 | 3152,7 ± 630,4 | | 8917,2 ± 3813,9 | 2284,6 ± 163 | | 2994,4 ± 273,6 |
| 230438_at | T-box 15 | | TBX15 | | 3,03 | 117,4 ± 15,4 | | 489 ± 468,2 | 113,3 ± 9,2 | | 96,2 ± 15,6 |
| 208336_s_at | trans-2,3-enoyl-CoA reductase | | TECR* | | 3,65 | 613,2 ± 93,9 | | 2464,4 ± 1784,9 | 480,5 ± 19,4 | | 768,1 ± 97 |
| 203400_s_at | transferrin | | TF | | 174,18 | 13,6 ± 13,6 | | 3107 ± 4089,6 | 588 ± 12,7 | | 77 ± 45,2 |
| 228716_at | thyroid hormone receptor, beta (erythroblastic leukemia viral (v-erb-a) oncogene homolog 2, avian) | | THRB* | | 3,85 | 35,3 ± 15,7 | | 113,9 ± 15,2 | 76,1 ± 9,8 | | 66,1 ± 6,3 |
| 229477_at | thyroid hormone responsive (SPOT14 homolog, rat) | | THRSP | | 158,80 | 17,9 ± 8 | | 7009,5 ± 9143,2 | 44,2 ± 1,6 | | 18 ± 9,2 |
| 206243_at | TIMP metallopeptidase inhibitor 4 | | TIMP4* | | 90,51 | 100,6 ± 16,4 | | 11703,1 ± 8618,3 | 525,5 ± 53,9 | | 158,9 ± 45,3 |
| 200803_s_at | transmembrane BAX inhibitor motif containing 6 | | TMBIM6 | | 2,28 | 3005,2 ± 489,7 | | 7520,9 ± 3808,3 | 2799,5 ± 131 | | 3230,2 ± 223,4 |
| 213349_at | transmembrane and coiled-coil domain family 1 | | TMCC1 | | 2,18 | 154,7 ± 22,7 | | 277,7 ± 63,1 | 213,6 ± 49 | | 155,8 ± 12 |
| 203437_at | transmembrane protein 11 | | TMEM11 | | 2,26 | 261,8 ± 25,8 | | 541 ± 159,6 | 196,6 ± 5,8 | | 285,2 ± 59,9 |
| 213851_at | transmembrane protein 110 | | TMEM110 | | 3,15 | 98,7 ± 38,1 | | 490,6 ± 230,3 | 131,1 ± 14,7 | | 142,6 ± 1,2 |
| 223186_at | transmembrane protein 189 /// ubiquitin-conjugating enzyme E2 variant 1 | | TMEM189 /// UBE2V1 | | 3,30 | 339,3 ± 36,8 | | 924 ± 334,2 | 301,9 ± 3,4 | | 259,6 ± 11,9 |
| 219569_s_at | transmembrane protein 22 | | TMEM22 | | 3,08 | 303 ± 32,8 | | 849,6 ± 294,8 | 237,6 ± 17,9 | | 331,3 ± 36,4 |
| 212282_at | transmembrane protein 97 | | TMEM97 | | 3,51 | 199,7 ± 16,5 | | 741,8 ± 333,1 | 237,3 ± 22,9 | | 83,1 ± 16,6 |
| 201175_at | thioredoxin-related transmembrane protein 2 | | TMX2 | | 2,48 | 1058,1 ± 214,7 | | 2472,3 ± 615,2 | 770,2 ± 83,8 | | 1296,7 ± 156,6 |
| 218856_at | tumor necrosis factor receptor superfamily, member 21 | | TNFRSF21 | | 2,92 | 352,3 ± 72,7 | | 1307,9 ± 1221,2 | 285,1 ± 32 | | 375,1 ± 51,9 |
| 204079_at | tyrosylprotein sulfotransferase 2 | | TPST2 | | 2,32 | 414,7 ± 20,4 | | 894,2 ± 330,4 | 463,8 ± 48,5 | | 514,4 ± 25,8 |
| 208763_s_at | TSC22 domain family, member 3 | | TSC22D3 | | 17,15 | 379,6 ± 115,6 | | 8640,7 ± 7404,8 | 432,7 ± 28,6 | | 739,4 ± 31,1 |
| 218245_at | tsukushin | | TSKU | | 4,67 | 214,2 ± 16,2 | | 1213,5 ± 791,3 | 159,5 ± 18,1 | | 252,4 ± 23,1 |
| 217979_at | tetraspanin 13 | | TSPAN13 | | 3,05 | 200,4 ± 120 | | 1038,9 ± 547,2 | 302,1 ± 30,1 | | 727,6 ± 116,5 |
| 221002_s_at | tetraspanin 14 | | TSPAN14 | | 2,89 | 276,2 ± 41,3 | | 932 ± 465,4 | 301,1 ± 2,2 | | 445,2 ± 26,4 |
| 200973_s_at | tetraspanin 3 | | TSPAN3 | | 2,33 | 1258,6 ± 180,6 | | 2938 ± 555,6 | 1400 ± 27,8 | | 1814,5 ± 175,2 |
| 209605_at | thiosulfate sulfurtransferase (rhodanese) | | TST | | 5,92 | 378,8 ± 38,4 | | 3074,1 ± 2160 | 375,1 ± 28,6 | | 452,2 ± 42,2 |
| 230747_s_at | tetratricopeptide repeat domain 39C | | TTC39C | | 2,70 | 170,1 ± 71,6 | | 441 ± 78,9 | 159,4 ± 7 | | 149,6 ± 7,1 |
| 218837_s_at | ubiquitin-conjugating enzyme E2D 4 (putative) | | UBE2D4 | | 2,18 | 227,1 ± 16,2 | | 461,1 ± 87,5 | 227,3 ± 24,7 | | 302,4 ± 23,7 |
| 226135_at | UHRF1 binding protein 1 | | UHRF1BP1 | | 2,92 | 137,2 ± 19,7 | | 326,7 ± 91,4 | 172,6 ± 39,6 | | 102,8 ± 10,6 |
| 224881_at | vitamin K epoxide reductase complex, subunit 1-like 1 | | VKORC1L1 | | 4,39 | 298,7 ± 48,1 | | 1432,7 ± 820 | 357,7 ± 7,6 | | 332,8 ± 30 |
| 201420_s_at | WD repeat domain 77 | | WDR77 | | 2,52 | 240,1 ± 76,1 | | 624,4 ± 233,1 | 205 ± 13,5 | | 197,2 ± 13,7 |
| 1555270_a_at | Wolfram syndrome 1 (wolframin) | | WFS1* | | 3,30 | 98,7 ± 9,6 | | 299 ± 131,9 | 136,6 ± 36,6 | | 143,4 ± 37,4 |
| 217785_s_at | YKT6 v-SNARE homolog (S. cerevisiae) | | YKT6* | | 2,99 | 119,2 ± 54 | | 407,1 ± 162,5 | 88,2 ± 18,1 | | 117,6 ± 8 |
| 222985_at | tyrosine 3-monooxygenase | | YWHAG | | 2,09 | 3774,8 ± 379,8 | | 8579,4 ± 2464,7 | 3161,6 ± 102,7 | | 3838 ± 256 |
| 205883_at | zinc finger and BTB domain containing 16 | | ZBTB16 | | 27,01 | 17,7 ± 3,7 | | 852,2 ± 668 | 35,1 ± 12,7 | | 22,3 ± 1 |
| 222451_s_at | zinc finger, DHHC-type containing 9 | | ZDHHC9 | | 2,79 | 412,6 ± 77,3 | | 1186,5 ± 404,6 | 510,7 ± 50,8 | | 928,3 ± 169,8 |
| **Cluster 2 genes** | | | | | | | | | | | |
| 63825_at | | abhydrolase domain containing 2 | ABHD2 | -3,51 | | 458,1 ± 48,6 | 217,6 ± 123,8 | | 346,2 ± 12,2 | 969,4 ± 156,8 | |
| 239909_at | | ADAMTS-like 1 | ADAMTSL1 | -24,63 | | 180,5 ± 33,4 | 15,8 ± 22,3 | | 125 ± 9,4 | 344,3 ± 20,7 | |
| 203741_s_at | | adenylate cyclase 7 | ADCY7 | -9,33 | | 370,8 ± 148,9 | 53,9 ± 42,7 | | 127,2 ± 5,5 | 346,3 ± 33,2 | |
| 205481_at | | adenosine A1 receptor | ADORA1* | -7,24 | | 257,7 ± 181,5 | 31,3 ± 20,7 | | 148,3 ± 5,8 | 799,5 ± 138,9 | |
| 221761_at | | adenylosuccinate synthase | ADSS | -2,18 | | 653,3 ± 106,7 | 302,2 ± 90,1 | | 467,4 ± 67,2 | 609,8 ± 31,2 | |
| 212992_at | | AHNAK nucleoprotein 2 | AHNAK2 | -4,42 | | 387,4 ± 29,9 | 89,2 ± 57,5 | | 200,5 ± 8,6 | 495,7 ± 8 | |
| 229354_at | | aryl-hydrocarbon receptor repressor | AHRR | -4,42 | | 1119,1 ± 691,9 | 217,9 ± 72,6 | | 346,5 ± 18,4 | 907,3 ± 127,4 | |
| 206561_s_at | | aldo-keto reductase family 1, member B10 (aldose reductase) | AKR1B10* | -3,59 | | 379,1 ± 83,2 | 117,7 ± 37 | | 181 ± 13,7 | 666,3 ± 132,1 | |
| 228255_at | | amyotrophic lateral sclerosis 2 (juvenile) chromosome region, candidate 4 | ALS2CR4 | -10,72 | | 134,6 ± 43,5 | 42,9 ± 49,3 | | 112,7 ± 14,7 | 169,2 ± 9,1 | |
| 220076_at | | ankylosis, progressive homolog (mouse) | ANKH | -31,75 | | 298,1 ± 148,1 | 21,7 ± 31,5 | | 57,7 ± 5,3 | 533,3 ± 33,2 | |
| 238332_at | | ankyrin repeat domain 29 | ANKRD29 | -10,00 | | 867,4 ± 208,9 | 142,4 ± 102,9 | | 333,7 ± 9,9 | 847,7 ± 31,6 | |
| 202888_s_at | | alanyl (membrane) aminopeptidase | ANPEP | -3,22 | | 1593,4 ± 509,4 | 628,7 ± 411,4 | | 1011,1 ± 7,8 | 3019,6 ± 376,8 | |
| 210143_at | | annexin A10 | ANXA10* | -9,19 | | 280 ± 77,7 | 25,5 ± 17,1 | | 160,5 ± 7,5 | 368,5 ± 19,9 | |
| 235548_at | | adenomatosis polyposis coli down-regulated 1-like | APCDD1L | -4,59 | | 916,9 ± 272,8 | 167,6 ± 152,4 | | 343 ± 36,4 | 1163,6 ± 22,9 | |
| 218501_at | | Rho guanine nucleotide exchange factor (GEF) 3 | ARHGEF3 | -9,26 | | 1643 ± 362 | 247,2 ± 171,1 | | 1161,9 ± 24,9 | 3004,7 ± 65,5 | |
| 203404_at | | armadillo repeat containing, X-linked 2 | ARMCX2 | -3,03 | | 3168,9 ± 511,9 | 1217 ± 768,1 | | 2731 ± 144,3 | 3802,8 ± 154 | |
| 230275_at | | arylsulfatase family, member I | ARSI | -12,90 | | 103 ± 17,6 | 12 ± 13,2 | | 35 ± 16,7 | 76,9 ± 18,1 | |
| 202686_s_at | | AXL receptor tyrosine kinase | AXL | -5,53 | | 2069,6 ± 394,6 | 475,4 ± 339,8 | | 1449,4 ± 73,3 | 2231,9 ± 147,5 | |
| 211812_s_at | | beta-1,3-N-acetylgalactosaminyltransferase 1 (globoside blood group) | B3GALNT1 | -14,59 | | 121,3 ± 35,5 | 18,7 ± 28,8 | | 62,9 ± 11,1 | 90,2 ± 13,3 | |
| 204908_s_at | | B-cell CLL/lymphoma 3 | BCL3 | -2,16 | | 216,5 ± 46,8 | 110,8 ± 37 | | 179 ± 23 | 382,3 ± 70,9 | |
| 207510_at | | bradykinin receptor B1 | BDKRB1* | -16,37 | | 389,1 ± 142,9 | 42 ± 49,6 | | 70,6 ± 1,4 | 346,7 ± 16,3 | |
| 205870_at | | bradykinin receptor B2 | BDKRB2* | -12,80 | | 527,2 ± 146,3 | 98,5 ± 117,1 | | 176,6 ± 45,2 | 528,5 ± 109 | |
| 221530_s_at | | basic helix-loop-helix family, member e41 | BHLHE41 | -5,00 | | 949,6 ± 433,1 | 196,8 ± 89,7 | | 612,5 ± 13,9 | 1706,6 ± 45,5 | |
| 201641_at | | bone marrow stromal cell antigen 2 | BST2* | -5,36 | | 157,3 ± 35,6 | 33,9 ± 15,5 | | 132,2 ± 20 | 279,5 ± 6 | |
| 204820_s_at | | butyrophilin, subfamily 3, member A2 /// butyrophilin, subfamily 3, member A3 | BTN3A2 /// BTN3A3 | -2,13 | | 1556,3 ± 267,6 | 758,9 ± 142,3 | | 1721,4 ± 120 | 3169,5 ± 27,9 | |
| 204821_at | | butyrophilin, subfamily 3, member A3 | BTN3A3 | -2,52 | | 367 ± 59,6 | 157,6 ± 25,2 | | 469,6 ± 24 | 927,1 ± 45 | |
| 224910_at | | calcium regulated heat stable protein 1, 24kDa | CARHSP1 | -2,62 | | 355,2 ± 46,6 | 159,3 ± 55,8 | | 297,6 ± 15,5 | 438,7 ± 24 | |
| 216598_s_at | | chemokine (C-C motif) ligand 2 | CCL2* | -43,88 | | 2268,3 ± 986,3 | 159,3 ± 173,8 | | 1509 ± 28,5 | 6413,4 ± 568,8 | |
| 228748_at | | CD59 molecule, complement regulatory protein | CD59* | -6,16 | | 217 ± 43 | 48,9 ± 38,4 | | 129,1 ± 9,9 | 310,9 ± 12,3 | |
| 209619_at | | CD74 molecule, major histocompatibility complex, class II invariant chain | CD74* | -7,02 | | 1172,4 ± 369,1 | 195,1 ± 139,7 | | 1556,6 ± 137 | 4298,6 ± 1664,7 | |
| 218451_at | | CUB domain containing protein 1 | CDCP1 | -20,95 | | 335,5 ± 204,1 | 13,1 ± 9,9 | | 50,4 ± 3 | 282,4 ± 13,1 | |
| 208807_s_at | | chromodomain helicase DNA binding protein 3 | CHD3 | -2,56 | | 275,1 ± 49,7 | 135,8 ± 36,7 | | 218,4 ± 17,4 | 361 ± 18,6 | |
| 203044_at | | chondroitin sulfate synthase 1 | CHSY1 | -2,09 | | 1162,9 ± 143,3 | 582,3 ± 194,7 | | 806,6 ± 15,5 | 892,9 ± 49,3 | |
| 226824_at | | carboxypeptidase X (M14 family), member 2 | CPXM2 | -2,72 | | 214,2 ± 69,3 | 84,2 ± 21 | | 179,6 ± 4,9 | 323 ± 35,5 | |
| 219049_at | | chondroitin sulfate N-acetylgalactosaminyltransferase 1 | CSGALNACT1 | -2,81 | | 215,7 ± 61,5 | 52,3 ± 8 | | 87,6 ± 14,4 | 298,7 ± 60,4 | |
| 204470_at | | chemokine (C-X-C motif) ligand 1 (melanoma growth stimulating activity, alpha) | CXCL1 | -3,05 | | 174,2 ± 63 | 65,6 ± 64,1 | | 55,5 ± 11,6 | 99,6 ± 26,6 | |
| 206336_at | | chemokine (C-X-C motif) ligand 6 (granulocyte chemotactic protein 2) | CXCL6 | -14,93 | | 1129,9 ± 774,4 | 52,1 ± 28,5 | | 85,6 ± 11,8 | 680,5 ± 176,6 | |
| 1568868_at | | cytochrome P450, family 27, subfamily C, polypeptide 1 | CYP27C1 | -3,82 | | 65,6 ± 28 | 14,6 ± 8,9 | | 27,9 ± 7,4 | 85,6 ± 19,4 | |
| 224911_s_at | | discoidin, CUB and LCCL domain containing 2 | DCBLD2 | -10,00 | | 3564,7 ± 1621,2 | 434,6 ± 426,1 | | 1474,5 ± 71,7 | 2198,3 ± 124,7 | |
| 203409_at | | damage-specific DNA binding protein 2, 48kDa | DDB2 | -2,24 | | 596,2 ± 119,5 | 288,2 ± 122,6 | | 657,4 ± 87,4 | 986,3 ± 38,2 | |
| 213279_at | | dehydrogenase/reductase (SDR family) member 1 | DHRS1 | -2,94 | | 408,3 ± 99 | 204,7 ± 125,7 | | 453,4 ± 25,7 | 876,6 ± 71,3 | |
| 214247_s_at | | dickkopf homolog 3 (Xenopus laevis) | DKK3 | -7,02 | | 4361,1 ± 1061,5 | 871,5 ± 933 | | 734,4 ± 43,9 | 5429,1 ± 170,5 | |
| 227081_at | | dynein, axonemal, light intermediate chain 1 | DNALI1 | -19,10 | | 124,7 ± 14,2 | 17,1 ± 25,6 | | 114,3 ± 9,7 | 262,7 ± 71,3 | |
| 230263_s_at | | Dedicator of cytokinesis 5 | DOCK5 | -5,79 | | 894,6 ± 86,8 | 222 ± 226,3 | | 389,3 ± 46,4 | 980,6 ± 161,6 | |
| 201431_s_at | | dihydropyrimidinase-like 3 | DPYSL3 | -7,24 | | 903,7 ± 195,5 | 141,9 ± 125 | | 590,1 ± 11,7 | 1130,7 ± 40,5 | |
| 201983_s_at | | epidermal growth factor receptor (erythroblastic leukemia viral (v-erb-b) oncogene homolog, avian) | EGFR | -3,46 | | 906,5 ± 285,4 | 259,4 ± 142,9 | | 473 ± 12,4 | 859,6 ± 82,4 | |
| 205249_at | | early growth response 2 | EGR2* | -12,41 | | 86,3 ± 28,8 | 7,6 ± 2,7 | | 16,6 ± 12,7 | 127,4 ± 23,6 | |
| 221755_at | | EH domain binding protein 1-like 1 | EHBP1L1 | -6,96 | | 128,9 ± 25,6 | 19,5 ± 22,3 | | 50,4 ± 13,6 | 179,3 ± 21,7 | |
| 201808_s_at | | endoglin | ENG | -7,70 | | 197,8 ± 24,3 | 36,1 ± 37,3 | | 84,6 ± 18 | 320,7 ± 19,4 | |
| 222603_at | | endoplasmic reticulum metallopeptidase 1 | ERMP1 | -2,19 | | 206,9 ± 32,6 | 106,5 ± 13 | | 184,3 ± 16,4 | 299,3 ± 9,7 | |
| 208394_x_at | | endothelial cell-specific molecule 1 | ESM1* | -12,31 | | 262,7 ± 159,2 | 44 ± 40,5 | | 75,1 ± 15,4 | 178,6 ± 15,7 | |
| 221911_at | | ets variant 1 | ETV1 | -25,59 | | 1624 ± 286 | 85,8 ± 70 | | 318,1 ± 31,7 | 2112,7 ± 164,1 | |
| 213506_at | | coagulation factor II (thrombin) receptor-like 1 | F2RL1 | -30,55 | | 333 ± 101,4 | 19,1 ± 18,9 | | 72,8 ± 7,7 | 248,6 ± 20,7 | |
| 223019_at | | family with sequence similarity 129, member B | FAM129B | -2,44 | | 1643,9 ± 478,3 | 526,5 ± 190,1 | | 868,5 ± 51,6 | 2499,8 ± 829,2 | |
| 230869_at | | family with sequence similarity 155, member A | FAM155A | -5,97 | | 180,2 ± 73,3 | 34,4 ± 9,3 | | 148,8 ± 40,3 | 406,3 ± 27,5 | |
| 227828_s_at | | family with sequence similarity 176, member A | FAM176A | -9,48 | | 658,6 ± 171,8 | 122,8 ± 147,7 | | 322,9 ± 14 | 530,8 ± 50,9 | |
| 226722_at | | family with sequence similarity 20, member C | FAM20C | -6,25 | | 3327,6 ± 992,8 | 515,4 ± 287,5 | | 1968 ± 199,3 | 4138,7 ± 105,3 | |
| 1559141_s_at | | family with sequence similarity 87, member A | FAM87A | -18,81 | | 311,4 ± 134,7 | 14,3 ± 9,7 | | 132,5 ± 19,3 | 422 ± 77,6 | |
| 212169_at | | FK506 binding protein 9, 63 kDa | FKBP9 | -2,11 | | 2496,2 ± 416,5 | 1204 ± 405,5 | | 2056,1 ± 149,3 | 3236,3 ± 193,9 | |
| 208614_s_at | | filamin B, beta | FLNB | -2,62 | | 406,8 ± 63,9 | 167,7 ± 56 | | 223,4 ± 14,8 | 467,6 ± 47,1 | |
| 226930_at | | fibronectin type III domain containing 1 | FNDC1 | -15,51 | | 450,8 ± 337,8 | 62,7 ± 48,7 | | 128,6 ± 21,7 | 285,1 ± 39,9 | |
| 202838_at | | fucosidase, alpha-L- 1, tissue | FUCA1 | -3,15 | | 713,8 ± 133,3 | 262,2 ± 146,5 | | 809,7 ± 34,8 | 1357,6 ± 116,7 | |
| 218204_s_at | | FYVE and coiled-coil domain containing 1 | FYCO1 | -2,83 | | 1057 ± 350,7 | 397 ± 108,5 | | 939,5 ± 109 | 2470,7 ± 89,6 | |
| 228303_at | | UDP-N-acetyl-alpha-D-galactosamine:polypeptide N-acetylgalactosaminyltransferase 6 (GalNAc-T6) | GALNT6 | -4,09 | | 74,1 ± 19,6 | 21,5 ± 18 | | 28,4 ± 4,7 | 74,2 ± 21,5 | |
| 204471_at | | growth associated protein 43 | GAP43* | -45,96 | | 794,1 ± 239,6 | 35,6 ± 47,1 | | 152,5 ± 17,2 | 1475,6 ± 108,7 | |
| 227376_at | | GLI family zinc finger 3 | GLI3 | -3,05 | | 421,8 ± 58,1 | 185,1 ± 38,1 | | 299,1 ± 20,2 | 423,3 ± 72 | |
| 221447_s_at | | glycosyltransferase 8 domain containing 2 | GLT8D2 | -8,77 | | 1661,4 ± 254,1 | 311,9 ± 333,8 | | 1101,7 ± 38,6 | 1726,3 ± 268,6 | |
| 204115_at | | guanine nucleotide binding protein (G protein), gamma 11 | GNG11* | -2,94 | | 2625,8 ± 1253,9 | 790 ± 76,1 | | 953,7 ± 40,1 | 3471,1 ± 93,8 | |
| 204854_at | | G protein-coupled receptor 162 /// leprecan-like 2 | GPR162 /// LEPREL2 | -3,35 | | 338 ± 26,8 | 76,9 ± 24,5 | | 184,5 ± 26,8 | 440,6 ± 30 | |
| 240509_s_at | | gremlin 2, cysteine knot superfamily, homolog (Xenopus laevis) | GREM2 | -30,55 | | 125,9 ± 45,2 | 19,5 ± 31,9 | | 25,5 ± 4,8 | 77,7 ± 9,4 | |
| 212206_s_at | | H2A histone family, member V | H2AFV | -2,83 | | 386,9 ± 103,1 | 127,7 ± 90,5 | | 235,6 ± 3,5 | 328,9 ± 31 | |
| 213537_at | | major histocompatibility complex, class II, DP alpha 1 | HLA-DPA1* | -5,44 | | 424,7 ± 60 | 80,3 ± 8,5 | | 343,9 ± 14,1 | 920,7 ± 94,6 | |
| 213831_at | | major histocompatibility complex, class II, DQ alpha 1 | HLA-DQA1* | -22,80 | | 115,6 ± 20,8 | 6,3 ± 6,5 | | 96,9 ± 16,3 | 479,5 ± 77,5 | |
| 209480_at | | major histocompatibility complex, class II, DQ beta 1 | HLA-DQB1* | -47,03 | | 270,9 ± 82,9 | 4,6 ± 6,3 | | 156 ± 7,6 | 1126,3 ± 172,7 | |
| 215193_x_at | | major histocompatibility complex, class II, DR beta 1 /// major histocompatibility complex, class II, DR beta 3 /// major histocompatibility complex, class II, DR beta 4 | HLA-DRB1 /// HLA-DRB3 /// HLA-DRB4* | -10,89 | | 4124,7 ± 992,2 | 336,4 ± 76,9 | | 3416,9 ± 167 | 8106,5 ± 564,6 | |
| 206074_s_at | | high mobility group AT-hook 1 | HMGA1 | -4,29 | | 1580,5 ± 619,9 | 528,2 ± 311,7 | | 887,6 ± 70,7 | 2931,4 ± 746,3 | |
| 235521_at | | homeobox A3 | HOXA3 | -4,42 | | 470,4 ± 69,9 | 128,2 ± 90,3 | | 273,5 ± 10,4 | 597,1 ± 19,7 | |
| 228904_at | | homeobox B3 | HOXB3 | -2,94 | | 1011,2 ± 100,2 | 408,4 ± 149,2 | | 720,8 ± 8,5 | 1124,8 ± 76,9 | |
| 205579_at | | histamine receptor H1 | HRH1* | -10,97 | | 184,2 ± 7 | 51,8 ± 43,7 | | 92,6 ± 10,7 | 208,8 ± 54,5 | |
| 212221_x_at | | iduronate 2-sulfatase | IDS | -4,35 | | 839,6 ± 132,2 | 253,4 ± 177,3 | | 601,3 ± 35,2 | 1170,1 ± 22,1 | |
| 203819_s_at | | insulin-like growth factor 2 mRNA binding protein 3 | IGF2BP3 | -22,11 | | 527,6 ± 424 | 49,4 ± 72,1 | | 102,6 ± 11,8 | 287,4 ± 58,2 | |
| 212143_s_at | | insulin-like growth factor binding protein 3 | IGFBP3* | -13,10 | | 7063,5 ± 405,7 | 640,4 ± 450,5 | | 3085,8 ± 209,6 | 9303,7 ± 420,1 | |
| 203233_at | | interleukin 4 receptor | IL4R | -2,94 | | 265,3 ± 23,7 | 104,6 ± 62,6 | | 95,2 ± 18,4 | 313,4 ± 22,3 | |
| 205207_at | | interleukin 6 (interferon, beta 2) | IL6* | -14,15 | | 666,6 ± 326,3 | 38 ± 25,9 | | 245,6 ± 12,3 | 513,5 ± 32,2 | |
| 207191_s_at | | immunoglobulin superfamily containing leucine-rich repeat | ISLR | -5,83 | | 1250,6 ± 467,6 | 236,2 ± 161 | | 634,3 ± 18,7 | 1567,5 ± 485,7 | |
| 227314_at | | integrin, alpha 2 (CD49B, alpha 2 subunit of VLA-2 receptor) | ITGA2 | -8,38 | | 353,1 ± 170,3 | 39,8 ± 15,4 | | 66,3 ± 10,7 | 222,9 ± 57,2 | |
| 221584_s_at | | potassium large conductance calcium-activated channel, subfamily M, alpha member 1 | KCNMA1* | -2,56 | | 1574,6 ± 371,6 | 685,3 ± 70,1 | | 921,6 ± 41,3 | 2094,6 ± 349,6 | |
| 204401_at | | potassium intermediate/small conductance calcium-activated channel, subfamily N, member 4 | KCNN4 | -7,07 | | 200,2 ± 62,8 | 28,9 ± 25,2 | | 71,1 ± 15 | 396,1 ± 15,7 | |
| 244623_at | | potassium voltage-gated channel, KQT-like subfamily, member 5 | KCNQ5 | -30,32 | | 125,6 ± 61,8 | 7,9 ± 10,8 | | 22,8 ± 11,1 | 27,9 ± 19,2 | |
| 38892_at | | KIAA0240 | KIAA0240 | -2,30 | | 190,7 ± 49,1 | 87,5 ± 25,3 | | 191,5 ± 18,6 | 289,2 ± 26,8 | |
| 231807_at | | KIAA1217 | KIAA1217 | -5,83 | | 375,4 ± 68,7 | 98,5 ± 109,7 | | 332,2 ± 48,1 | 549,3 ± 93,8 | |
| 52837_at | | KIAA1644 | KIAA1644 | -3,01 | | 866,1 ± 46,7 | 395,7 ± 250,5 | | 137,5 ± 6 | 1085,2 ± 34,5 | |
| 229891_x_at | | KIAA1704 | KIAA1704 | -2,19 | | 324,9 ± 38,3 | 146,5 ± 58,7 | | 303,1 ± 4,9 | 399,5 ± 24,2 | |
| 207723_s_at | | killer cell lectin-like receptor subfamily C, member 3 | KLRC3 | -9,40 | | 103,5 ± 16,5 | 12,3 ± 11,6 | | 15,4 ± 2,2 | 303,7 ± 16,6 | |
| 205821_at | | killer cell lectin-like receptor subfamily K, member 1 | KLRK1 | -13,20 | | 115,3 ± 15,6 | 8,7 ± 9,7 | | 31,2 ± 3,6 | 513,1 ± 85,4 | |
| 201650_at | | keratin 19 | KRT19* | -55,29 | | 336,5 ± 214,2 | 7,2 ± 10,5 | | 69,1 ± 9,4 | 136 ± 9,6 | |
| 210663_s_at | | kynureninase (L-kynurenine hydrolase) | KYNU | -15,28 | | 497,8 ± 274,3 | 31,3 ± 27,6 | | 209,5 ± 10,6 | 823,6 ± 148,4 | |
| 203726_s_at | | laminin, alpha 3 | LAMA3 | -20,79 | | 832,1 ± 231,3 | 71,5 ± 59,4 | | 384,7 ± 24,4 | 1601,3 ± 189,8 | |
| 1554679_a_at | | lysosomal protein transmembrane 4 beta | LAPTM4B | -2,81 | | 430,2 ± 62,4 | 161,1 ± 40,9 | | 225,7 ± 26,4 | 264,1 ± 87,8 | |
| 204249_s_at | | LIM domain only 2 (rhombotin-like 1) | LMO2 | -4,09 | | 708 ± 511,5 | 148,8 ± 34,6 | | 398,5 ± 56,4 | 1370,3 ± 47,6 | |
| 227889_at | | lysophosphatidylcholine acyltransferase 2 | LPCAT2* | -6,86 | | 248,1 ± 140,2 | 33,9 ± 26,9 | | 83,9 ± 30,8 | 103 ± 15 | |
| 206953_s_at | | latrophilin 2 | LPHN2 | -2,54 | | 1063,3 ± 275,7 | 460,9 ± 170,1 | | 794,2 ± 70,7 | 1112,4 ± 171,9 | |
| 216250_s_at | | leupaxin | LPXN | -2,64 | | 345,2 ± 120,8 | 142,9 ± 56,6 | | 270,3 ± 13,4 | 531,7 ± 10,5 | |
| 1556427_s_at | | LRRN4 C-terminal like | LRRN4CL | -4,59 | | 700,8 ± 591,1 | 101,8 ± 30,6 | | 873 ± 96,3 | 3390,3 ± 122,4 | |
| 202729_s_at | | latent transforming growth factor beta binding protein 1 | LTBP1 | -6,02 | | 3639 ± 415,3 | 720,3 ± 497,3 | | 1780,1 ± 34,7 | 5634,2 ± 256,3 | |
| 223690_at | | latent transforming growth factor beta binding protein 2 | LTBP2 | -6,30 | | 2480 ± 747,4 | 596,7 ± 447,8 | | 1515,9 ± 15,2 | 3529,1 ± 176,9 | |
| 219922_s_at | | latent transforming growth factor beta binding protein 3 | LTBP3 | -3,56 | | 536 ± 139,8 | 197,3 ± 99,1 | | 373,2 ± 17,9 | 748,7 ± 89 | |
| 209373_at | | mal, T-cell differentiation protein-like | MALL* | -6,02 | | 419,7 ± 201,4 | 96,1 ± 56,6 | | 96,8 ± 9,8 | 602,6 ± 50,1 | |
| 229021_at | | multiple C2 domains, transmembrane 2 | MCTP2* | -5,20 | | 106,5 ± 14,2 | 20 ± 21,5 | | 23,5 ± 8,5 | 43,3 ± 13,7 | |
| 217553_at | | STEAP family protein MGC87042 | MGC87042 | -2,42 | | 332,9 ± 144,3 | 138 ± 48,5 | | 203,6 ± 14,1 | 256,2 ± 11,5 | |
| 37408_at | | mannose receptor, C type 2 | MRC2 | -4,32 | | 3477,4 ± 621,3 | 915,1 ± 218,6 | | 2175,6 ± 36,8 | 4546,3 ± 135,2 | |
| 205076_s_at | | myotubularin related protein 11 | MTMR11 | -4,81 | | 257,8 ± 7,6 | 65,8 ± 44,4 | | 252 ± 22,1 | 531,6 ± 65,7 | |
| 202180_s_at | | major vault protein | MVP | -2,32 | | 844,2 ± 236,5 | 350,8 ± 72,3 | | 623,1 ± 63,6 | 1125,5 ± 38,2 | |
| 227584_at | | neuron navigator 1 | NAV1 | -3,20 | | 566,2 ± 37,5 | 282,9 ± 193,3 | | 258,1 ± 17,7 | 748,3 ± 45,7 | |
| 206022_at | | Norrie disease (pseudoglioma) | NDP | -32,25 | | 408,5 ± 316,2 | 9,8 ± 5 | | 117,9 ± 18,1 | 1297,5 ± 61,9 | |
| 224368_s_at | | NDRG family member 3 | NDRG3 | -2,87 | | 230 ± 1,5 | 99,6 ± 54 | | 191,4 ± 5,8 | 314,4 ± 5 | |
| 201829_at | | neuroepithelial cell transforming 1 | NET1 | -4,16 | | 1547,9 ± 482,6 | 515,8 ± 393,1 | | 1039,4 ± 47 | 1503,1 ± 271 | |
| 218888_s_at | | neuropilin (NRP) and tolloid (TLL)-like 2 | NETO2 | -9,26 | | 2373 ± 808,2 | 359,6 ± 299,1 | | 472,7 ± 22,8 | 821,7 ± 11,9 | |
| 236270_at | | nuclear factor of activated T-cells, cytoplasmic, calcineurin-dependent 4 | NFATC4 | -4,13 | | 265,4 ± 96,2 | 63,5 ± 4,3 | | 182,4 ± 17,8 | 362,1 ± 6 | |
| 210276_s_at | | nucleolar protein 12 /// TRIO and F-actin binding protein | NOL12 /// TRIOBP | -3,91 | | 1436,3 ± 586,7 | 374,9 ± 129,2 | | 898,9 ± 11,8 | 1956,1 ± 78,9 | |
| 203939_at | | 5'-nucleotidase, ecto (CD73) | NT5E* | -17,28 | | 5126,4 ± 1127,2 | 379,4 ± 366,4 | | 2278,5 ± 168,6 | 5374,9 ± 260,6 | |
| 227566_at | | neurotrimin | NTM* | -40,94 | | 2630,3 ± 633,8 | 88,8 ± 88,1 | | 475,6 ± 76,3 | 3318,5 ± 235,5 | |
| 223315_at | | netrin 4 | NTN4 | -4,52 | | 922,4 ± 378,8 | 323,9 ± 229,9 | | 315 ± 18 | 1351 ± 51,5 | |
| 231406_at | | ORAI calcium release-activated calcium modulator 2 | ORAI2 | -2,68 | | 356,9 ± 51,4 | 157,9 ± 38,5 | | 311,1 ± 3 | 476,4 ± 28,2 | |
| 205729_at | | oncostatin M receptor | OSMR | -3,76 | | 367 ± 63,8 | 120 ± 41,4 | | 331,9 ± 52,2 | 532,6 ± 59,7 | |
| 214620_x_at | | peptidylglycine alpha-amidating monooxygenase | PAM | -3,59 | | 2166 ± 667,7 | 434,4 ± 335,2 | | 2337,3 ± 359,6 | 5002,4 ± 235,3 | |
| 202239_at | | poly (ADP-ribose) polymerase family, member 4 | PARP4 | -2,60 | | 791,1 ± 140,3 | 411,4 ± 185,4 | | 666,7 ± 25 | 1184,3 ± 126 | |
| 228635_at | | protocadherin 10 | PCDH10 | -5,28 | | 159,4 ± 7,8 | 43,8 ± 36,9 | | 59,7 ± 12,3 | 165,8 ± 17,6 | |
| 230109_at | | phosphodiesterase 7B | PDE7B | -8,91 | | 1309,2 ± 93 | 218,2 ± 163 | | 623,8 ± 8,1 | 927,5 ± 36,7 | |
| 222719_s_at | | platelet derived growth factor C | PDGFC | -5,12 | | 438,8 ± 25,3 | 100,8 ± 41,4 | | 154,8 ± 16,9 | 304,9 ± 14,4 | |
| 213791_at | | proenkephalin | PENK | -7,94 | | 10389,3 ± 2898,1 | 1321,3 ± 943 | | 1329,1 ± 162,9 | 9316,7 ± 1370,4 | |
| 209652_s_at | | placental growth factor | PGF | -3,30 | | 888,7 ± 222,8 | 310 ± 159,2 | | 305,4 ± 18,5 | 542,4 ± 38,5 | |
| 227211_at | | PHD finger protein 19 | PHF19 | -8,71 | | 175,6 ± 53,8 | 49,2 ± 46,7 | | 107,8 ± 10 | 230,8 ± 34,1 | |
| 225842_at | | pleckstrin homology-like domain, family A, member 1 | PHLDA1 | -25,40 | | 3715,1 ± 1570,6 | 148,2 ± 67,7 | | 1492,5 ± 90,7 | 3816,1 ± 92 | |
| 226864_at | | Protein kinase (cAMP-dependent, catalytic) inhibitor alpha | PKIA | -2,42 | | 75,1 ± 9,7 | 32,3 ± 11,2 | | 51,1 ± 17,5 | 62,2 ± 6,4 | |
| 235746_s_at | | phospholipase A2 receptor 1, 180kDa | PLA2R1 | -6,25 | | 138 ± 31,8 | 29,2 ± 20,1 | | 82,1 ± 12,1 | 190,4 ± 30,9 | |
| 214866_at | | plasminogen activator, urokinase receptor | PLAUR* | -5,74 | | 494,9 ± 198,2 | 103,8 ± 57,6 | | 286,5 ± 13,3 | 660,2 ± 56,1 | |
| 225136_at | | pleckstrin homology domain containing, family A (phosphoinositide binding specific) member 2 | PLEKHA2* | -2,83 | | 1313,6 ± 188,1 | 502,4 ± 351,7 | | 1021,9 ± 96 | 2164,5 ± 100,8 | |
| 227276_at | | plexin domain containing 2 | PLXDC2 | -3,85 | | 177,7 ± 105,5 | 55 ± 28,3 | | 72,7 ± 8,2 | 126,4 ± 15 | |
| 204285_s_at | | phorbol-12-myristate-13-acetate-induced protein 1 | PMAIP1 | -10,00 | | 226,9 ± 48,6 | 52 ± 64,5 | | 100,4 ± 4,5 | 129,9 ± 19,7 | |
| 203407_at | | periplakin | PPL | -6,11 | | 759,6 ± 382 | 144,8 ± 72,8 | | 446,2 ± 22,4 | 2736 ± 56,7 | |
| 206007_at | | proteoglycan 4 | PRG4 | -7,07 | | 77,8 ± 2,6 | 11,2 ± 5,2 | | 17,8 ± 2,4 | 284,6 ± 27,4 | |
| 226069_at | | prickle homolog 1 (Drosophila) | PRICKLE1* | -4,42 | | 193 ± 65 | 49,8 ± 15,3 | | 162,6 ± 24 | 397,5 ± 11,3 | |
| 213093_at | | protein kinase C, alpha | PRKCA* | -2,11 | | 1000,5 ± 81,6 | 492,3 ± 150,3 | | 598,2 ± 37,6 | 1546,3 ± 77,8 | |
| 207388_s_at | | prostaglandin E synthase | PTGES* | -6,06 | | 367,9 ± 35,3 | 97,7 ± 66,9 | | 112 ± 5,6 | 268,5 ± 22,4 | |
| 208617_s_at | | protein tyrosine phosphatase type IVA, member 2 | PTP4A2* | -2,18 | | 4153,7 ± 601,7 | 1953,2 ± 974,5 | | 2849,2 ± 149,3 | 3735,2 ± 86,5 | |
| 225796_at | | PX domain containing serine/threonine kinase | PXK* | -2,44 | | 668,9 ± 235,4 | 258,8 ± 35,8 | | 448 ± 46,4 | 592,6 ± 88,3 | |
| 211974_x_at | | recombination signal binding protein for immunoglobulin kappa J region | RBPJ | -4,92 | | 3669,3 ± 894,2 | 951,9 ± 804 | | 2428,3 ± 37,7 | 3749,4 ± 281 | |
| 209684_at | | Ras and Rab interactor 2 | RIN2 | -3,46 | | 1192,7 ± 218,9 | 299,8 ± 180,2 | | 905,5 ± 83,1 | 1608,3 ± 162,9 | |
| 219457_s_at | | Ras and Rab interactor 3 | RIN3 | -8,91 | | 65,7 ± 14,4 | 5,7 ± 1,9 | | 37,7 ± 7,9 | 87,2 ± 12,5 | |
| 223843_at | | scavenger receptor class A, member 3 | SCARA3 | -8,84 | | 659,1 ± 162,5 | 67,6 ± 15,8 | | 278,9 ± 15,7 | 1265,2 ± 21,9 | |
| 202656_s_at | | SERTA domain containing 2 | SERTAD2 | -2,32 | | 1168,6 ± 91,4 | 582,8 ± 182,1 | | 879,3 ± 71,2 | 1888,9 ± 121,6 | |
| 230660_at | | SERTA domain containing 4 | SERTAD4 | -19,40 | | 217,2 ± 41,4 | 16,3 ± 14,3 | | 47,3 ± 2,5 | 325,7 ± 26,1 | |
| 220974_x_at | | sideroflexin 3 | SFXN3 | -2,39 | | 421 ± 44,3 | 204,9 ± 106,9 | | 325,5 ± 19,7 | 613,6 ± 81,2 | |
| 203320_at | | SH2B adaptor protein 3 | SH2B3* | -2,23 | | 794,7 ± 282,7 | 307,7 ± 60,9 | | 250,8 ± 6,7 | 786,2 ± 135,7 | |
| 215856_at | | sialic acid binding Ig-like lectin 15 | SIGLEC15 | -19,55 | | 184,6 ± 62,9 | 16,9 ± 23 | | 90,5 ± 47,5 | 319,4 ± 26,6 | |
| 202254_at | | Signal-induced proliferation-associated 1 like 1 | SIPA1L1 | -8,57 | | 252,3 ± 28,4 | 77,2 ± 86,6 | | 153,7 ± 15,9 | 243,5 ± 34,6 | |
| 205856_at | | solute carrier family 14 (urea transporter), member 1 (Kidd blood group) | SLC14A1 | -32,75 | | 3015 ± 794,9 | 206,1 ± 339 | | 526,1 ± 36,6 | 5695,5 ± 1220,4 | |
| 205234_at | | solute carrier family 16, member 4 (monocarboxylic acid transporter 5) | SLC16A4 | -5,70 | | 1706 ± 636,7 | 297,8 ± 237,4 | | 1228,4 ± 395,4 | 2838,1 ± 257,1 | |
| 222088_s_at | | solute carrier family 2 (facilitated glucose transporter), member 14 /// solute carrier family 2 (facilitated glucose transporter), member 3 | SLC2A14 /// SLC2A3 | -3,56 | | 248,7 ± 58,8 | 91,1 ± 59,2 | | 216,3 ± 28,1 | 301,3 ± 12 | |
| 228754_at | | solute carrier family 6 (neurotransmitter transporter, taurine), member 6 | SLC6A6 | -3,85 | | 1385,4 ± 69,3 | 435,2 ± 317,9 | | 957,8 ± 82,1 | 1462,9 ± 98,7 | |
| 209679_s_at | | small trans-membrane and glycosylated protein | SMAGP | -3,30 | | 646,1 ± 181,2 | 191,2 ± 59,1 | | 319,6 ± 16,3 | 627,2 ± 47,9 | |
| 202043_s_at | | spermine synthase | SMS | -3,10 | | 1494,2 ± 388,1 | 563 ± 222,8 | | 891 ± 124,4 | 1059,7 ± 56,3 | |
| 213493_at | | sushi, nidogen and EGF-like domains 1 | SNED1 | -9,93 | | 583,5 ± 347,4 | 59,3 ± 32,4 | | 479,1 ± 75,4 | 1753,1 ± 253,2 | |
| 209875_s_at | | secreted phosphoprotein 1 | SPP1* | -5,97 | | 118,2 ± 42 | 30,3 ± 26,7 | | 124,3 ± 24,7 | 308 ± 40,2 | |
| 212458_at | | sprouty-related, EVH1 domain containing 2 | SPRED2 | -2,08 | | 850,4 ± 58,9 | 403 ± 162,7 | | 693,3 ± 33,4 | 1293,3 ± 93,8 | |
| 225095_at | | Serine palmitoyltransferase, long chain base subunit 2 | SPTLC2* | -4,42 | | 232,5 ± 17,7 | 83,3 ± 52,5 | | 126,9 ± 4,6 | 191,3 ± 23,9 | |
| 210073_at | | ST8 alpha-N-acetyl-neuraminide alpha-2,8-sialyltransferase 1 | ST8SIA1* | -7,13 | | 212,4 ± 44,7 | 27,9 ± 6,7 | | 62,9 ± 6,7 | 448 ± 41,8 | |
| 218424_s_at | | STEAP family member 3 | STEAP3 | -2,60 | | 823,5 ± 215,1 | 275,7 ± 75,5 | | 304 ± 10 | 1090,5 ± 121,7 | |
| 202566_s_at | | supervillin | SVIL | -4,19 | | 835,9 ± 82,5 | 237,5 ± 57,2 | | 486,1 ± 27,2 | 1038,3 ± 96 | |
| 209197_at | | synaptotagmin XI | SYT11* | -3,91 | | 467,1 ± 91,9 | 136,7 ± 76,9 | | 365,6 ± 10,9 | 841 ± 110,5 | |
| 209153_s_at | | transcription factor 3 (E2A immunoglobulin enhancer binding factors E12/E47) | TCF3 | -2,62 | | 263,4 ± 38,8 | 128,7 ± 17,5 | | 224 ± 5,6 | 351 ± 31,1 | |
| 209277_at | | tissue factor pathway inhibitor 2 | TFPI2 | -17,15 | | 1961,2 ± 1835,6 | 99,4 ± 91,5 | | 91,2 ± 6,7 | 2911,7 ± 220,6 | |
| 208944_at | | transforming growth factor, beta receptor II (70/80kDa) | TGFBR2* | -2,92 | | 4881 ± 503,1 | 1947,5 ± 1130,6 | | 3143,1 ± 85,8 | 7857 ± 452 | |
| 209561_at | | thrombospondin 3 | THBS3 | -2,60 | | 405,2 ± 54,4 | 161,2 ± 55,8 | | 378,4 ± 19,1 | 554,8 ± 20 | |
| 213135_at | | T-cell lymphoma invasion and metastasis 1 | TIAM1* | -6,16 | | 294,6 ± 115,3 | 44,2 ± 17,5 | | 252,5 ± 5,2 | 512,2 ± 18,5 | |
| 201147_s_at | | TIMP metallopeptidase inhibitor 3 | TIMP3* | -5,04 | | 3130,6 ± 584 | 841,5 ± 709,8 | | 1854,8 ± 245,3 | 10856,5 ± 193,4 | |
| 209386_at | | transmembrane 4 L six family member 1 | TM4SF1 | -5,66 | | 8714,2 ± 917,3 | 2605,8 ± 2117,4 | | 7355,5 ± 188,8 | 14012,8 ± 934 | |
| 226050_at | | transmembrane and coiled-coil domains 3 | TMCO3 | -2,26 | | 2989 ± 153,8 | 1339,7 ± 667,1 | | 2315,1 ± 62,7 | 3498,5 ± 430 | |
| 212507_at | | transmembrane protein 131 | TMEM131 | -2,66 | | 826,6 ± 142,7 | 337 ± 132,2 | | 595,5 ± 124,2 | 719,4 ± 119,6 | |
| 213338_at | | transmembrane protein 158 | TMEM158 | -6,35 | | 1852,4 ± 546,1 | 313,3 ± 189,6 | | 595,8 ± 17,6 | 3303,2 ± 173,1 | |
| 228054_at | | transmembrane protein 44 | TMEM44 | -15,28 | | 107,5 ± 34,3 | 7,4 ± 9,3 | | 66,5 ± 6,9 | 143,8 ± 19,2 | |
| 210643_at | | tumor necrosis factor (ligand) superfamily, member 11 | TNFSF11 | -8,44 | | 181,2 ± 132,2 | 16,2 ± 6,7 | | 25,8 ± 4,6 | 169,9 ± 48,5 | |
| 209863_s_at | | tumor protein p63 | TP63 | -8,51 | | 79,1 ± 41,3 | 11,1 ± 11,8 | | 31,9 ± 9,9 | 185,4 ± 13,9 | |
| 202478_at | | tribbles homolog 2 (Drosophila) | TRIB2 | -5,00 | | 863,1 ± 163,2 | 187,3 ± 120,3 | | 421,5 ± 26,3 | 1163,5 ± 101,8 | |
| 202795_x_at | | TRIO and F-actin binding protein | TRIOBP | -3,20 | | 1597,9 ± 401,4 | 581,8 ± 91,7 | | 1065,8 ± 85,3 | 2433 ± 13,9 | |
| 225485_at | | testis specific, 14 | TSGA14 | -2,99 | | 126,6 ± 19,4 | 61,4 ± 22,2 | | 114,5 ± 19,4 | 147,8 ± 10,6 | |
| 223393_s_at | | teashirt zinc finger homeobox 3 | TSHZ3 | -2,33 | | 276,3 ± 65,9 | 138,4 ± 62,5 | | 179,1 ± 26,4 | 346,5 ± 15,6 | |
| 209264_s_at | | tetraspanin 4 | TSPAN4 | -3,03 | | 1111,1 ± 84,5 | 437,9 ± 244,2 | | 699,6 ± 44,2 | 1367,9 ± 37,2 | |
| 213058_at | | tetratricopeptide repeat domain 28 | TTC28 | -2,54 | | 603 ± 145,6 | 211,7 ± 112,6 | | 509,8 ± 62,4 | 698,9 ± 81,9 | |
| 218449_at | | UFM1-specific peptidase 2 | UFSP2 | -2,92 | | 609,3 ± 60,5 | 286,9 ± 197,6 | | 466,2 ± 17,5 | 651,4 ± 47,7 | |
| 225610_at | | ubiquitin-like with PHD and ring finger domains 2 | UHRF2 | -2,60 | | 809,1 ± 180,3 | 302,9 ± 78,9 | | 619,5 ± 33 | 1103,6 ± 9,9 | |
| 235751_s_at | | vitelline membrane outer layer 1 homolog (chicken) | VMO1 | -6,65 | | 875,3 ± 134,1 | 194,6 ± 222,6 | | 67,7 ± 12 | 508,4 ± 101,5 | |
| 225898_at | | WD repeat domain 54 | WDR54 | -2,66 | | 623,5 ± 83 | 222,7 ± 108,4 | | 418,6 ± 3,5 | 901,6 ± 36,6 | |
| 202664_at | | WAS/WASL interacting protein family, member 1 | WIPF1 | -4,78 | | 728,5 ± 89,2 | 173,8 ± 106,5 | | 413,2 ± 17,9 | 990,4 ± 13,3 | |
| 213425_at | | wingless-type MMTV integration site family, member 5A | WNT5A* | -13,20 | | 2807,9 ± 880,2 | 287,5 ± 339 | | 357 ± 34,2 | 1862,2 ± 196,4 | |
| 202133_at | | WW domain containing transcription regulator 1 | WWTR1 | -3,40 | | 4547,6 ± 834,2 | 1558,4 ± 893 | | 2737,5 ± 281,1 | 4229,6 ± 325,7 | |
| 228046_at | | Zinc finger protein 827 | ZNF827 | -3,13 | | 539,5 ± 59,2 | 204,9 ± 86,6 | | 462,6 ± 54,3 | 630,7 ± 41,1 | |
| **Cluster 3 genes** | | | | | | | | | | | |
| 217504_at | | ATP-binding cassette, sub-family A (ABC1), member 6 | ABCA6 | -2,62 | | 158,2 ± 40,7 | 53 ± 30,6 | | 172,9 ± 26,1 | 153,9 ± 4,8 | |
| 225098_at | | abl-interactor 2 | ABI2 | -3,38 | | 435,9 ± 84,2 | 143,8 ± 80,1 | | 340,1 ± 24,5 | 436,3 ± 31,2 | |
| 226426_at | | activity-dependent neuroprotector homeobox | ADNP* | -2,68 | | 338 ± 78,7 | 123,3 ± 79,8 | | 289,3 ± 45,9 | 308,6 ± 5,9 | |
| 1558142_at | | adenylosuccinate lyase /// trinucleotide repeat containing 6B | ADSL /// TNRC6B | -3,35 | | 326,7 ± 15,1 | 101,9 ± 37,1 | | 354,3 ± 23 | 275,2 ± 58,4 | |
| 202820_at | | aryl hydrocarbon receptor | AHR | -4,35 | | 1199,2 ± 237 | 364,8 ± 331 | | 966,8 ± 95,2 | 1009 ± 65,7 | |
| 222862_s_at | | adenylate kinase 5 | AK5 | -15,51 | | 324,1 ± 168,5 | 34,3 ± 46,4 | | 134,6 ± 13,1 | 113 ± 25,9 | |
| 213035_at | | ankyrin repeat domain 28 | ANKRD28 | -3,91 | | 410,6 ± 85,7 | 121,4 ± 63,3 | | 494,1 ± 45,1 | 286,9 ± 29,6 | |
| 224694_at | | anthrax toxin receptor 1 | ANTXR1 | -2,21 | | 2284,5 ± 559,8 | 1072,4 ± 535,2 | | 2959,7 ± 165 | 2109,7 ± 225,6 | |
| 228573_at | | anthrax toxin receptor 2 | ANTXR2 | -3,13 | | 651,2 ± 81,6 | 222,9 ± 93,5 | | 526,8 ± 13 | 701,3 ± 45,8 | |
| 226358_at | | anterior pharynx defective 1 homolog B (C. elegans) | APH1B | -2,44 | | 200,3 ± 23,8 | 86,5 ± 9 | | 235,7 ± 19,8 | 351,7 ± 16,1 | |
| 219637_at | | armadillo repeat containing 9 | ARMC9 | -5,83 | | 369,1 ± 67,1 | 51,6 ± 39,3 | | 457,8 ± 37,2 | 309,8 ± 31,4 | |
| 227444_at | | Armadillo repeat containing, X-linked 4 | ARMCX4 | -6,65 | | 163,6 ± 37,4 | 43,9 ± 48,8 | | 126,4 ± 12,6 | 161,3 ± 17,4 | |
| 206414_s_at | | ArfGAP with SH3 domain, ankyrin repeat and PH domain 2 | ASAP2 | -2,42 | | 479 ± 77 | 195,3 ± 44,5 | | 535,7 ± 6,7 | 535,1 ± 48 | |
| 226513_at | | ankyrin repeat and SOCS box-containing 7 | ASB7 | -2,23 | | 188,2 ± 8,1 | 82,6 ± 12 | | 189,4 ± 31,4 | 189,4 ± 22,3 | |
| 226251_at | | additional sex combs like 2 (Drosophila) | ASXL2 | -2,37 | | 179,8 ± 27 | 83,4 ± 24,8 | | 215,5 ± 48,4 | 162,7 ± 18,8 | |
| 226302_at | | ATPase, class I, type 8B, member 1 | ATP8B1* | -8,84 | | 524,2 ± 27,3 | 115 ± 150,5 | | 517,2 ± 16,9 | 360,6 ± 40,4 | |
| 203232_s_at | | ataxin 1 | ATXN1 | -3,08 | | 315,2 ± 105,6 | 99,5 ± 59,7 | | 249,4 ± 62,8 | 257,8 ± 29,3 | |
| 227083_at | | beta 1,3-galactosyltransferase-like | B3GALTL | -3,43 | | 141,9 ± 18,3 | 30,3 ± 7,9 | | 128,7 ± 9,1 | 86,2 ± 4,4 | |
| 218899_s_at | | brain and acute leukemia, cytoplasmic | BAALC* | -6,16 | | 233,9 ± 68,3 | 66,3 ± 77,5 | | 289,3 ± 12,4 | 182,1 ± 52 | |
| 217867_x_at | | beta-site APP-cleaving enzyme 2 | BACE2 | -2,02 | | 364,2 ± 86,1 | 148,8 ± 14,7 | | 575,2 ± 30,3 | 502,9 ± 39,2 | |
| 202391_at | | brain abundant, membrane attached signal protein 1 | BASP1* | -3,40 | | 4210,2 ± 291,6 | 1302,4 ± 660,2 | | 4363,3 ± 116,1 | 3714 ± 225,6 | |
| 208445_s_at | | bromodomain adjacent to zinc finger domain, 1B | BAZ1B | -2,30 | | 468 ± 13,1 | 218,4 ± 76 | | 493 ± 38,2 | 442,1 ± 29,6 | |
| 37549_g_at | | Bardet-Biedl syndrome 9 | BBS9* | -2,83 | | 224,7 ± 41,8 | 86,9 ± 72,1 | | 243,3 ± 33,2 | 235,7 ± 15,7 | |
| 218285_s_at | | 3-hydroxybutyrate dehydrogenase, type 2 | BDH2* | -2,21 | | 1326,1 ± 105,7 | 693,8 ± 340,3 | | 1596,4 ± 71,2 | 1784,3 ± 168,7 | |
| 231175_at | | BEN domain containing 6 | BEND6 | -4,81 | | 301,5 ± 22,7 | 61,3 ± 39,5 | | 349,5 ± 34,4 | 176,9 ± 31,4 | |
| 201261_x_at | | biglycan | BGN | -2,60 | | 533,1 ± 175,1 | 221,4 ± 79 | | 544,9 ± 40 | 535,6 ± 24,1 | |
| 222761_at | | basic, immunoglobulin-like variable motif containing | BIVM | -3,46 | | 513,9 ± 35 | 173,8 ± 104,2 | | 598,7 ± 48,9 | 434,1 ± 43,4 | |
| 229942_at | | basonuclin 2 | BNC2 | -6,81 | | 227,6 ± 8,2 | 38,2 ± 20,9 | | 212 ± 12,8 | 115,6 ± 23,4 | |
| 212613_at | | butyrophilin, subfamily 3, member A2 | BTN3A2 | -2,44 | | 278,9 ± 39,1 | 108,8 ± 23,6 | | 335,8 ± 8 | 490 ± 25 | |
| 202357_s_at | | complement component 2 /// complement factor B | C2 /// CFB | -10,72 | | 299,1 ± 117,4 | 35,5 ± 23,5 | | 395,1 ± 24,8 | 555,9 ± 18,9 | |
| 204508_s_at | | carbonic anhydrase XII | CA12 | -5,16 | | 1627,6 ± 634 | 314,5 ± 197,4 | | 1700,1 ± 53,2 | 2852,3 ± 45,5 | |
| 221565_s_at | | calcium homeostasis modulator 2 | CALHM2 | -5,44 | | 433,1 ± 104,2 | 75 ± 57,4 | | 529 ± 37,7 | 837,6 ± 99,3 | |
| 231793_s_at | | calcium/calmodulin-dependent protein kinase II delta | CAMK2D | -2,56 | | 528,1 ± 126 | 202,9 ± 94,2 | | 713,8 ± 53,7 | 588,8 ± 40,8 | |
| 1552701_a_at | | caspase recruitment domain family, member 16 | CARD16 | -6,02 | | 150,2 ± 18,3 | 31,6 ± 28,9 | | 277,9 ± 17,2 | 102,1 ± 8,9 | |
| 224414_s_at | | caspase recruitment domain family, member 6 | CARD6 | -5,24 | | 703,3 ± 218 | 167,8 ± 125,6 | | 663,3 ± 12,9 | 1102,5 ± 15,2 | |
| 209310_s_at | | caspase 4, apoptosis-related cysteine peptidase | CASP4 | -2,60 | | 435,6 ± 54,3 | 180,6 ± 17,5 | | 468,7 ± 18,1 | 442,3 ± 4,9 | |
| 212586_at | | calpastatin | CAST | -5,12 | | 2724,9 ± 249 | 946,2 ± 956,3 | | 2309 ± 48,8 | 2188,5 ± 55,1 | |
| 218802_at | | coiled-coil domain containing 109B | CCDC109B | -3,56 | | 753,3 ± 76,5 | 263,6 ± 139,3 | | 606 ± 48,1 | 622,2 ± 43,9 | |
| 226287_at | | coiled-coil domain containing 34 | CCDC34 | -3,97 | | 371,2 ± 126,6 | 100,5 ± 33,6 | | 278,7 ± 20,5 | 261,3 ± 12 | |
| 227966_s_at | | coiled-coil domain containing 74A /// coiled-coil domain containing 74B | CCDC74A /// CCDC74B | -3,79 | | 208,4 ± 20,4 | 58,6 ± 51,7 | | 229,3 ± 14 | 245,6 ± 31,2 | |
| 226545_at | | CD109 molecule | CD109* | -14,04 | | 2014,6 ± 206,8 | 258,1 ± 295,8 | | 1176,5 ± 68,7 | 822,4 ± 95,8 | |
| 226016_at | | CD47 molecule | CD47 | -6,81 | | 1028,2 ± 63,2 | 306,8 ± 280,5 | | 927,1 ± 76,2 | 1008,9 ± 37,2 | |
| 201028_s_at | | CD99 molecule | CD99 | -2,33 | | 3949,6 ± 116,8 | 1722,6 ± 497,1 | | 4585,3 ± 218,6 | 4717,9 ± 820,6 | |
| 203854_at | | complement factor I | CFI | -28,95 | | 1602,5 ± 552 | 66,2 ± 54,7 | | 1611,3 ± 31,8 | 1214,4 ± 73,7 | |
| 225077_at | | chromodomain helicase DNA binding protein 2 | CHD2 | -2,37 | | 477,1 ± 62 | 197,7 ± 105,5 | | 563,3 ± 30,6 | 379,1 ± 13,7 | |
| 209396_s_at | | chitinase 3-like 1 (cartilage glycoprotein-39) | CHI3L1 | -268,11 | | 5868,1 ± 798,6 | 32,8 ± 21,4 | | 5402,2 ± 421,9 | 3693,4 ± 656,2 | |
| 228335_at | | claudin 11 | CLDN11 | -5,61 | | 7565,5 ± 1660,2 | 1139,5 ± 703,6 | | 5965,8 ± 85,5 | 10715 ± 505,3 | |
| 223047_at | | CKLF-like MARVEL transmembrane domain containing 6 | CMTM6 | -3,03 | | 1515,7 ± 63 | 565,4 ± 450,4 | | 1681,4 ± 95,3 | 1083,2 ± 109,1 | |
| 219400_at | | contactin associated protein 1 | CNTNAP1 | -6,20 | | 166,6 ± 11,8 | 29 ± 22,1 | | 191,2 ± 5 | 322,9 ± 30,1 | |
| 212865_s_at | | collagen, type XIV, alpha 1 | COL14A1 | -20,00 | | 2438,8 ± 1294,3 | 225 ± 303,5 | | 1812,3 ± 111,2 | 3506,2 ± 137,6 | |
| 221019_s_at | | collectin sub-family member 12 | COLEC12 | -14,36 | | 1992,1 ± 1070,3 | 124,5 ± 66,8 | | 2545 ± 87,7 | 2014,7 ± 68,2 | |
| 201943_s_at | | carboxypeptidase D | CPD* | -2,85 | | 281,7 ± 48,1 | 115,5 ± 53,5 | | 227,4 ± 19,3 | 183,8 ± 18,5 | |
| 201990_s_at | | cAMP responsive element binding protein-like 2 | CREBL2 | -2,83 | | 534,5 ± 89,7 | 211,4 ± 119,8 | | 591,5 ± 23,4 | 500,9 ± 38,3 | |
| 223475_at | | cysteine-rich secretory protein LCCL domain containing 1 | CRISPLD1 | -5,08 | | 363,6 ± 97,2 | 78,7 ± 20,7 | | 580,6 ± 43,9 | 287,2 ± 10,4 | |
| 202646_s_at | | cold shock domain containing E1, RNA-binding | CSDE1 | -2,50 | | 2345,5 ± 113,2 | 1186,9 ± 803,5 | | 2660,3 ± 173,8 | 1863,3 ± 87,9 | |
| 202450_s_at | | cathepsin K | CTSK | -5,32 | | 3535,1 ± 3222 | 564,9 ± 332,3 | | 5974,1 ± 187,8 | 7440,5 ± 658 | |
| 201059_at | | cortactin | CTTN | -3,82 | | 2803,7 ± 253 | 810,6 ± 715,4 | | 2712,7 ± 73,6 | 2757,8 ± 209,1 | |
| 201278_at | | disabled homolog 2, mitogen-responsive phosphoprotein (Drosophila) | DAB2 | -7,13 | | 2161,4 ± 277,2 | 471,6 ± 505,7 | | 3161,1 ± 79,2 | 2687,9 ± 28,5 | |
| 228293_at | | DEP domain containing 7 | DEPDC7 | -4,25 | | 231,6 ± 46,5 | 72,1 ± 50 | | 210,2 ± 16 | 148,8 ± 20,8 | |
| 218277_s_at | | DEAH (Asp-Glu-Ala-His) box polypeptide 40 | DHX40 | -2,37 | | 796,3 ± 47,1 | 360,1 ± 130,9 | | 790,7 ± 51,7 | 610,1 ± 62,5 | |
| 202416_at | | DnaJ (Hsp40) homolog, subfamily C, member 7 | DNAJC7 | -2,39 | | 765,7 ± 34,1 | 300 ± 151 | | 649,7 ± 24,1 | 639,3 ± 27,3 | |
| 205003_at | | dedicator of cytokinesis 4 | DOCK4 | -5,66 | | 320,1 ± 51 | 65,1 ± 48,2 | | 242,4 ± 48,7 | 258,5 ± 29,1 | |
| 203716_s_at | | dipeptidyl-peptidase 4 | DPP4 | -26,81 | | 116 ± 49,9 | 3 ± 0,7 | | 265,1 ± 16,5 | 121,1 ± 24,9 | |
| 204646_at | | dihydropyrimidine dehydrogenase | DPYD | -4,96 | | 1226,3 ± 190,6 | 383,5 ± 341,6 | | 1185,6 ± 102,8 | 1340,8 ± 80,5 | |
| 218627_at | | DNA-damage regulated autophagy modulator 1 | DRAM1 | -5,20 | | 1022,9 ± 113,4 | 244,7 ± 133,8 | | 1190 ± 18,9 | 856,9 ± 44,7 | |
| 229017_s_at | | dual serine/threonine and tyrosine protein kinase | DSTYK | -2,19 | | 274,7 ± 30,4 | 158,4 ± 77,9 | | 260,1 ± 13,2 | 286,2 ± 12,5 | |
| 227084_at | | dystrobrevin, alpha | DTNA | -5,49 | | 163,9 ± 26,3 | 49,6 ± 50,1 | | 332,2 ± 34,4 | 393,5 ± 18,3 | |
| 225415_at | | deltex 3-like (Drosophila) | DTX3L | -2,28 | | 541,8 ± 119,9 | 236,5 ± 44 | | 681,9 ± 39,1 | 696,6 ± 69,8 | |
| 204557_s_at | | DAZ interacting protein 1 | DZIP1 | -2,70 | | 373,9 ± 12,3 | 127,6 ± 49,2 | | 394,7 ± 38,9 | 366,1 ± 39,9 | |
| 227243_s_at | | early B-cell factor 3 | EBF3 | -4,49 | | 694,2 ± 175,3 | 157,3 ± 84,3 | | 462 ± 26,5 | 583 ± 12,3 | |
| 225656_at | | EF-hand domain (C-terminal) containing 1 | EFHC1 | -4,35 | | 197,8 ± 31 | 51,4 ± 27,5 | | 226,6 ± 26,3 | 188,5 ± 21,6 | |
| 220591_s_at | | EF-hand domain (C-terminal) containing 2 | EFHC2 | -4,39 | | 156,6 ± 24,6 | 45,3 ± 31,4 | | 153,6 ± 18,2 | 170,7 ± 16,7 | |
| 227955_s_at | | ephrin-A5 | EFNA5* | -4,09 | | 528,5 ± 225,7 | 136,4 ± 116,6 | | 389 ± 36,7 | 477,4 ± 31,9 | |
| 221773_at | | ELK3, ETS-domain protein (SRF accessory protein 2) | ELK3 | -5,57 | | 2141 ± 136,3 | 411,2 ± 253,7 | | 1347,8 ± 190,3 | 1439,1 ± 61,4 | |
| 219134_at | | EGF, latrophilin and seven transmembrane domain containing 1 | ELTD1 | -10,00 | | 504 ± 154,3 | 59,5 ± 26,4 | | 300,3 ± 37,4 | 378,4 ± 31,9 | |
| 201719_s_at | | erythrocyte membrane protein band 4.1-like 2 | EPB41L2 | -3,88 | | 566,9 ± 140,7 | 173,7 ± 95,7 | | 571,5 ± 23,2 | 506 ± 48,1 | |
| 225838_at | | enhancer of polycomb homolog 2 (Drosophila) | EPC2 | -2,79 | | 293,4 ± 19,6 | 116,8 ± 52,4 | | 289,6 ± 16,1 | 218,5 ± 30,9 | |
| 227609_at | | epithelial stromal interaction 1 (breast) | EPSTI1 | -10,48 | | 511,3 ± 88,4 | 77,5 ± 75,6 | | 426,1 ± 53 | 584,6 ± 88,1 | |
| 219759_at | | endoplasmic reticulum aminopeptidase 2 | ERAP2 | -4,52 | | 260 ± 45 | 71,6 ± 58,4 | | 208,3 ± 9,8 | 166,5 ± 12,6 | |
| 231911_at | | ermin, ERM-like protein | ERMN | -11,49 | | 123,7 ± 13,5 | 15,5 ± 20,3 | | 87,2 ± 6,8 | 47,4 ± 13,4 | |
| 220134_x_at | | family with sequence similarity 176, member B | FAM176B | -2,32 | | 415,8 ± 73,8 | 205 ± 92,2 | | 459 ± 39,9 | 499,8 ± 25 | |
| 241981_at | | family with sequence similarity 20, member A | FAM20A | -28,73 | | 530,4 ± 420,4 | 37,6 ± 41,4 | | 551,3 ± 38,9 | 516,9 ± 25,9 | |
| 243606_at | | family with sequence similarity 55, member C | FAM55C | -3,56 | | 138,8 ± 32,5 | 69,5 ± 54,6 | | 201,2 ± 37,2 | 205 ± 18,3 | |
| 235318_at | | fibrillin 1 | FBN1 | -4,49 | | 566,5 ± 105,2 | 171 ± 173,3 | | 444,6 ± 22,2 | 391,2 ± 37,4 | |
| 202949_s_at | | four and a half LIM domains 2 | FHL2 | -5,32 | | 8439,7 ± 428,6 | 2385 ± 2560,8 | | 7056,3 ± 159,4 | 9485,9 ± 615,3 | |
| 1554424_at | | FIP1 like 1 (S. cerevisiae) | FIP1L1 | -5,12 | | 120,9 ± 18 | 28,5 ± 21,2 | | 71,3 ± 2,2 | 74,8 ± 24 | |
| 226184_at | | formin-like 2 | FMNL2 | -2,79 | | 1016,8 ± 269,5 | 376,6 ± 51,3 | | 775,9 ± 26,7 | 721,6 ± 64,1 | |
| 224838_at | | forkhead box P1 | FOXP1 | -5,49 | | 2156 ± 409,8 | 380 ± 186,5 | | 2344,2 ± 27,1 | 1793,3 ± 96,6 | |
| 203988_s_at | | fucosyltransferase 8 (alpha (1,6) fucosyltransferase) | FUT8 | -2,68 | | 698,2 ± 138,7 | 249,9 ± 46,2 | | 489,6 ± 18 | 507,5 ± 50,2 | |
| 224252_s_at | | FXYD domain containing ion transport regulator 5 | FXYD5 | -2,79 | | 536,2 ± 109,3 | 182,5 ± 25,6 | | 494,4 ± 25,7 | 611 ± 169,8 | |
| 210220_at | | frizzled homolog 2 (Drosophila) | FZD2 | -3,54 | | 459,8 ± 47,6 | 145,4 ± 69,1 | | 618,3 ± 41,7 | 671,6 ± 28,4 | |
| 206614_at | | growth differentiation factor 5 | GDF5 | -12,90 | | 417,1 ± 88,8 | 57,3 ± 57,8 | | 988,3 ± 66,6 | 1375,3 ± 85,4 | |
| 201667_at | | gap junction protein, alpha 1, 43kDa | GJA1 | -4,85 | | 11410,2 ± 1703 | 3413,3 ± 2913,7 | | 10445 ± 620,4 | 9867,2 ± 174,7 | |
| 212414_s_at | | glyoxylate reductase 1 homolog (Arabidopsis) /// septin 6 | GLYR1 /// SEPT6* | -4,96 | | 309,8 ± 63,3 | 75,1 ± 72 | | 268,9 ± 21,7 | 367,1 ± 19,8 | |
| 204875_s_at | | GDP-mannose 4,6-dehydratase | GMDS | -2,48 | | 313 ± 173,3 | 117,2 ± 18,1 | | 376,5 ± 16,9 | 456,8 ± 44,8 | |
| 217771_at | | golgi membrane protein 1 | GOLM1 | -7,07 | | 1290,2 ± 191,9 | 246,2 ± 188,2 | | 1482,6 ± 86,4 | 960,2 ± 171,6 | |
| 1554018_at | | glycoprotein (transmembrane) nmb | GPNMB | -7,52 | | 285,2 ± 121,7 | 48,2 ± 48,3 | | 541,4 ± 92,4 | 191,3 ± 24,8 | |
| 230369_at | | G protein-coupled receptor 161 | GPR161 | -3,94 | | 198,1 ± 28,9 | 50,3 ± 31,6 | | 163,4 ± 14,1 | 205,2 ± 6,7 | |
| 228027_at | | G protein-coupled receptor associated sorting protein 2 | GPRASP2 | -2,08 | | 532,9 ± 96,5 | 260,3 ± 52,9 | | 680,9 ± 28,6 | 838,1 ± 29,9 | |
| 218537_at | | host cell factor C1 regulator 1 (XPO1 dependent) | HCFC1R1 | -4,13 | | 456,6 ± 110 | 115,9 ± 83 | | 510 ± 22,7 | 708,4 ± 112 | |
| 225012_at | | high density lipoprotein binding protein | HDLBP* | -3,13 | | 529,1 ± 31,6 | 207,6 ± 65,2 | | 468,3 ± 25,9 | 541,6 ± 38,8 | |
| 218603_at | | headcase homolog (Drosophila) | HECA | -3,48 | | 643,6 ± 246,9 | 204,2 ± 127,4 | | 743,6 ± 51,4 | 538,5 ± 30,8 | |
| 232080_at | | HECT, C2 and WW domain containing E3 ubiquitin protein ligase 2 | HECW2 | -6,02 | | 340,4 ± 31,2 | 71,2 ± 91,4 | | 476,7 ± 51,7 | 717,5 ± 31,9 | |
| 203903_s_at | | hephaestin | HEPH | -3,79 | | 1993,9 ± 645,1 | 509 ± 73,7 | | 2742,6 ± 61,9 | 3407,5 ± 121,9 | |
| 209960_at | | hepatocyte growth factor (hepapoietin A; scatter factor) | HGF | -12,31 | | 761,9 ± 128,7 | 156,1 ± 180,7 | | 462,6 ± 87,3 | 370 ± 76,4 | |
| 208729_x_at | | major histocompatibility complex, class I, B | HLA-B* | -2,68 | | 3414,7 ± 1127,5 | 1092,4 ± 284,1 | | 4301,5 ± 184,8 | 4813 ± 217 | |
| 226878_at | | major histocompatibility complex, class II, DO alpha | HLA-DOA* | -14,04 | | 402,1 ± 161,9 | 25,9 ± 14,4 | | 495,5 ± 12,7 | 1269,6 ± 71,8 | |
| 210982_s_at | | major histocompatibility complex, class II, DR alpha | HLA-DRA* | -15,63 | | 3622,8 ± 1541,6 | 232,3 ± 40,7 | | 4042,8 ± 248,8 | 6476,3 ± 190,3 | |
| 209312_x_at | | major histocompatibility complex, class II, DR beta 1 /// major histocompatibility complex, class II, DR beta 4 /// major histocompatibility complex, class II, DR beta 5 | HLA-DRB1 /// HLA-DRB4 /// HLA-DRB5* | -10,89 | | 5715,9 ± 1674,8 | 497,6 ± 156,7 | | 5018,6 ± 107,8 | 11302,2 ± 119,1 | |
| 204806_x_at | | major histocompatibility complex, class I, F | HLA-F* | -2,35 | | 1224,6 ± 131,1 | 437,5 ± 68,7 | | 1296,6 ± 50,7 | 1386 ± 186,3 | |
| 208025_s_at | | high mobility group AT-hook 2 | HMGA2 | -16,63 | | 905,3 ± 195,4 | 67,8 ± 59,7 | | 471,7 ± 77,4 | 729,5 ± 23,9 | |
| 227139_s_at | | Hermansky-Pudlak syndrome 3 | HPS3 | -3,62 | | 842,4 ± 332,8 | 213 ± 60,3 | | 1209,8 ± 46,9 | 801,4 ± 43,2 | |
| 200941_at | | heat shock factor binding protein 1 | HSBP1 | -2,62 | | 1384,2 ± 121,9 | 597,5 ± 363,4 | | 1134,9 ± 63,6 | 1319 ± 171,7 | |
| 217989_at | | hydroxysteroid (17-beta) dehydrogenase 11 | HSD17B11* | -3,76 | | 865,8 ± 51,2 | 216,3 ± 116,8 | | 944,3 ± 57,1 | 796,5 ± 106 | |
| 226552_at | | immediate early response 5-like | IER5L | -8,25 | | 393,3 ± 181,9 | 79,8 ± 84,5 | | 480,9 ± 12,5 | 519,1 ± 26,2 | |
| 209721_s_at | | intermediate filament family orphan 1 | IFFO1 | -3,05 | | 500,6 ± 92,3 | 146,2 ± 20,9 | | 508,3 ± 34,5 | 680 ± 67,7 | |
| 208966_x_at | | interferon, gamma-inducible protein 16 | IFI16 | -9,19 | | 3408,1 ± 1030 | 434,5 ± 364,8 | | 4931,3 ± 233,4 | 1911,5 ± 80,1 | |
| 201422_at | | interferon, gamma-inducible protein 30 | IFI30 | -4,92 | | 1307,2 ± 221,8 | 227,8 ± 32,4 | | 1259,1 ± 108 | 871,7 ± 71,6 | |
| 214453_s_at | | interferon-induced protein 44 | IFI44 | -5,97 | | 598,8 ± 110,2 | 107,1 ± 52,5 | | 539,2 ± 46,3 | 341,1 ± 41,1 | |
| 204439_at | | interferon-induced protein 44-like | IFI44L | -28,07 | | 724,5 ± 222,7 | 38,4 ± 42,9 | | 680,5 ± 24,6 | 346,8 ± 38,6 | |
| 219209_at | | interferon induced with helicase C domain 1 | IFIH1* | -11,67 | | 71,9 ± 13 | 10,4 ± 7,6 | | 132,8 ± 18,4 | 92 ± 21,6 | |
| 203153_at | | interferon-induced protein with tetratricopeptide repeats 1 | IFIT1 | -7,94 | | 394,7 ± 161,4 | 97,5 ± 97,6 | | 697,3 ± 13,5 | 485,5 ± 10,9 | |
| 229450_at | | interferon-induced protein with tetratricopeptide repeats 3 | IFIT3 | -4,03 | | 627,8 ± 198,8 | 162,9 ± 106,9 | | 1162,1 ± 56,7 | 726 ± 43,5 | |
| 222519_s_at | | intraflagellar transport 57 homolog (Chlamydomonas) | IFT57 | -2,50 | | 294,5 ± 66,5 | 106,7 ± 43,9 | | 195,4 ± 32 | 198,8 ± 3,8 | |
| 203851_at | | insulin-like growth factor binding protein 6 | IGFBP6* | -8,71 | | 5621,8 ± 468,7 | 559,2 ± 316,8 | | 4767,6 ± 153,6 | 8381,3 ± 132,6 | |
| 231929_at | | IKAROS family zinc finger 2 (Helios) | IKZF2 | -8,57 | | 53,1 ± 12,5 | 5,5 ± 0,5 | | 56,8 ± 13,5 | 21,5 ± 7,5 | |
| 201887_at | | interleukin 13 receptor, alpha 1 | IL13RA1 | -3,94 | | 1474,5 ± 375,7 | 413,9 ± 223,3 | | 1319,1 ± 39,8 | 1296,2 ± 123,8 | |
| 206172_at | | interleukin 13 receptor, alpha 2 | IL13RA2 | -20,16 | | 228 ± 77,4 | 9,9 ± 12,6 | | 249,4 ± 46,7 | 690,8 ± 39,2 | |
| 227997_at | | interleukin 17 receptor D | IL17RD | -8,57 | | 426,1 ± 170,6 | 43,8 ± 12,8 | | 393,5 ± 33,3 | 476 ± 46,6 | |
| 203882_at | | interferon regulatory factor 9 | IRF9 | -3,27 | | 512,1 ± 18,7 | 172,6 ± 34 | | 711,8 ± 10,6 | 712 ± 58 | |
| 235182_at | | isthmin 1 homolog (zebrafish) | ISM1 | -5,92 | | 1123,1 ± 555,7 | 169 ± 76,5 | | 993,1 ± 24,3 | 1311,1 ± 78,5 | |
| 205885_s_at | | integrin, alpha 4 (antigen CD49D, alpha 4 subunit of VLA-4 receptor) | ITGA4 | -33,00 | | 53,5 ± 12,2 | 1,9 ± 1,3 | | 48,2 ± 12,8 | 16,4 ± 2,3 | |
| 1553678_a_at | | integrin, beta 1 (fibronectin receptor, beta polypeptide, antigen CD29 includes MDF2, MSK12) | ITGB1 | -3,35 | | 6581,3 ± 2076 | 2349,9 ± 2058 | | 4579,2 ± 281,9 | 3993 ± 154,7 | |
| 225806_at | | jub, ajuba homolog (Xenopus laevis) | JUB | -2,32 | | 453 ± 132,3 | 217,8 ± 116,2 | | 669,1 ± 16,7 | 528 ± 11,3 | |
| 218418_s_at | | KN motif and ankyrin repeat domains 2 | KANK2 | -2,37 | | 2492,9 ± 715,1 | 1024 ± 473,3 | | 2786,6 ± 188,1 | 4024,5 ± 269,6 | |
| 203845_at | | K(lysine) acetyltransferase 2B | KAT2B* | -2,37 | | 337,2 ± 56,5 | 172,1 ± 94,9 | | 335,2 ± 30,4 | 359,5 ± 75,7 | |
| 207103_at | | potassium voltage-gated channel, Shal-related subfamily, member 2 | KCND2 | -19,40 | | 139,6 ± 69,7 | 15 ± 20,8 | | 113 ± 12,3 | 132,2 ± 27,5 | |
| 206765_at | | potassium inwardly-rectifying channel, subfamily J, member 2 | KCNJ2 | -14,15 | | 648,4 ± 185,5 | 54,2 ± 17,1 | | 589,4 ± 19,9 | 532,6 ± 52,1 | |
| 217894_at | | potassium channel tetramerisation domain containing 3 | KCTD3 | -3,76 | | 530,6 ± 38,9 | 173,2 ± 109 | | 422 ± 32,4 | 341,9 ± 20,2 | |
| 225128_at | | KDEL (Lys-Asp-Glu-Leu) containing 2 | KDELC2 | -3,22 | | 512,3 ± 26 | 248,6 ± 165,2 | | 460,9 ± 38,3 | 380,1 ± 26,8 | |
| 229850_at | | 3-ketodihydrosphingosine reductase | KDSR | -3,25 | | 470,9 ± 120,4 | 159 ± 28,9 | | 620,7 ± 75,7 | 388,2 ± 26 | |
| 202503_s_at | | KIAA0101 | KIAA0101 | -22,28 | | 2859,5 ± 1063,5 | 304,6 ± 347,2 | | 1715,7 ± 96,7 | 1279,4 ± 68,4 | |
| 212314_at | | KIAA0746 protein | KIAA0746 | -5,49 | | 929,4 ± 185,8 | 208,4 ± 102,9 | | 1526,4 ± 50,8 | 1437,1 ± 195,4 | |
| 232155_at | | KIAA1618 | KIAA1618 | -9,93 | | 98,1 ± 24,4 | 10,4 ± 10,7 | | 60,9 ± 17,9 | 71,1 ± 15,1 | |
| 202202_s_at | | laminin, alpha 4 | LAMA4* | -3,03 | | 4151,3 ± 389,9 | 1661 ± 1142,6 | | 4262,6 ± 180,8 | 4510,9 ± 171 | |
| 211651_s_at | | laminin, beta 1 | LAMB1 | -2,44 | | 3074,3 ± 437,2 | 1420,6 ± 722,6 | | 2917,8 ± 107,5 | 3586,3 ± 149 | |
| 206481_s_at | | LIM domain binding 2 | LDB2 | -24,82 | | 1112,7 ± 402,7 | 136 ± 154,5 | | 779 ± 26,8 | 860,3 ± 19,3 | |
| 218717_s_at | | leprecan-like 1 | LEPREL1 | -3,35 | | 1215,2 ± 273,3 | 444,6 ± 142,4 | | 1143,1 ± 20,5 | 1210,3 ± 94,1 | |
| 218326_s_at | | leucine-rich repeat-containing G protein-coupled receptor 4 | LGR4 | -6,20 | | 667,1 ± 320,1 | 92,5 ± 34,9 | | 376,6 ± 32,6 | 335,5 ± 17 | |
| 224036_s_at | | limb region 1 homolog (mouse) | LMBR1 | -3,17 | | 175 ± 20,1 | 58,5 ± 28,4 | | 139,5 ± 23,2 | 162,4 ± 19,6 | |
| 204036_at | | lysophosphatidic acid receptor 1 | LPAR1* | -2,26 | | 2552,1 ± 81,7 | 1329,4 ± 813,2 | | 2052,7 ± 62,2 | 2285 ± 70,4 | |
| 214109_at | | LPS-responsive vesicle trafficking, beach and anchor containing | LRBA | -3,25 | | 243,3 ± 44,3 | 82,4 ± 45,8 | | 179,5 ± 17,2 | 222 ± 45,6 | |
| 226795_at | | leucine-rich repeats and calponin homology (CH) domain containing 1 | LRCH1 | -3,01 | | 161,5 ± 12,9 | 75,9 ± 48,8 | | 153,5 ± 18,8 | 157 ± 41,1 | |
| 203835_at | | leucine rich repeat containing 32 | LRRC32 | -4,39 | | 541,3 ± 200,3 | 158,1 ± 101,9 | | 941 ± 58,1 | 1075,7 ± 68,6 | |
| 218729_at | | latexin | LXN | -36,76 | | 5308 ± 1460,1 | 252,4 ± 310,8 | | 3961,9 ± 220,5 | 4178,2 ± 270,9 | |
| 205668_at | | lymphocyte antigen 75 | LY75 | -6,65 | | 181,5 ± 94,3 | 31,6 ± 19,6 | | 249,3 ± 11,2 | 164,5 ± 12,3 | |
| 206584_at | | lymphocyte antigen 96 | LY96* | -3,73 | | 1210,9 ± 93,2 | 334,7 ± 171,1 | | 1956,6 ± 74,9 | 1439,7 ± 116,7 | |
| 218181_s_at | | mitogen-activated protein kinase kinase kinase kinase 4 | MAP4K4 | -7,64 | | 2593,1 ± 257,4 | 418,4 ± 309,6 | | 2595,8 ± 125,2 | 2508,8 ± 190,3 | |
| 226726_at | | membrane bound O-acyltransferase domain containing 2 | MBOAT2* | -3,40 | | 540,4 ± 156,4 | 227,2 ± 100,7 | | 380,7 ± 9,6 | 434,1 ± 56,5 | |
| 226225_at | | mutated in colorectal cancers | MCC | -2,87 | | 455,9 ± 82,4 | 176,6 ± 43,9 | | 544 ± 49 | 464,5 ± 32,1 | |
| 203510_at | | met proto-oncogene (hepatocyte growth factor receptor) | MET | -4,74 | | 2003 ± 696,8 | 373,7 ± 248,5 | | 1085,3 ± 174,7 | 1008,5 ± 82,2 | |
| 204838_s_at | | mutL homolog 3 (E. coli) | MLH3 | -2,66 | | 175,1 ± 79,2 | 80,4 ± 2,9 | | 218,6 ± 27,4 | 169,5 ± 26 | |
| 226981_at | | Myeloid/lymphoid or mixed-lineage leukemia (trithorax homolog, Drosophila) | MLL | -2,46 | | 217,1 ± 17,9 | 109,3 ± 19,9 | | 259,1 ± 21,5 | 255,4 ± 47,2 | |
| 226100_at | | myeloid/lymphoid or mixed-lineage leukemia 5 (trithorax homolog, Drosophila) | MLL5 | -3,32 | | 253,4 ± 46,6 | 82,1 ± 32,9 | | 248,2 ± 50,2 | 176,2 ± 19,6 | |
| 218211_s_at | | melanophilin | MLPH | -3,43 | | 775,5 ± 145 | 281,9 ± 109,4 | | 830,4 ± 31,4 | 633 ± 14,1 | |
| 202911_at | | mutS homolog 6 (E. coli) | MSH6 | -2,09 | | 715,5 ± 94,6 | 376,7 ± 175,5 | | 723 ± 41,8 | 624,9 ± 21,3 | |
| 225237_s_at | | musashi homolog 2 (Drosophila) | MSI2 | -3,73 | | 135,5 ± 32,8 | 43,9 ± 28,2 | | 132,4 ± 6,8 | 119,4 ± 19,7 | |
| 203037_s_at | | metastasis suppressor 1 | MTSS1 | -4,96 | | 970,7 ± 870,8 | 178,7 ± 82,8 | | 2241,5 ± 102,7 | 1530,9 ± 140,2 | |
| 226547_at | | MYST histone acetyltransferase (monocytic leukemia) 3 | MYST3 | -2,18 | | 391,7 ± 15,2 | 177,9 ± 36,2 | | 427,5 ± 15,9 | 306,1 ± 22,5 | |
| 212462_at | | MYST histone acetyltransferase (monocytic leukemia) 4 | MYST4 | -3,25 | | 482,5 ± 58 | 182,3 ± 117,3 | | 415,4 ± 7,7 | 309 ± 43,3 | |
| 214693_x_at | | neuroblastoma breakpoint family, member 10 | NBPF10 | -2,58 | | 318,8 ± 107,2 | 130,2 ± 31,6 | | 511,5 ± 23,2 | 510,7 ± 87,5 | |
| 229461_x_at | | neuronal growth regulator 1 | NEGR1* | -10,72 | | 1319,5 ± 348,8 | 248,1 ± 348,1 | | 540,2 ± 75,5 | 542,4 ± 36,3 | |
| 204114_at | | nidogen 2 (osteonidogen) | NID2 | -4,03 | | 2609,4 ± 370,2 | 697,3 ± 338,9 | | 4413,5 ± 137,7 | 3693,9 ± 100,2 | |
| 207703_at | | neuroligin 4, Y-linked | NLGN4Y* | -10,72 | | 252,9 ± 24,6 | 24,4 ± 18,1 | | 122,7 ± 27,2 | 129,9 ± 7,9 | |
| 214722_at | | Notch homolog 2 (Drosophila) N-terminal like | NOTCH2NL | -3,76 | | 1247 ± 228,6 | 247,4 ± 111,4 | | 1686,7 ± 190,3 | 948,4 ± 169,8 | |
| 212298_at | | neuropilin 1 | NRP1 | -5,88 | | 2293,8 ± 203,4 | 674,6 ± 549,2 | | 1908,5 ± 76,2 | 2126,1 ± 68,1 | |
| 213125_at | | olfactomedin-like 2B | OLFML2B | -6,02 | | 621,9 ± 336,5 | 131,6 ± 102,3 | | 996,3 ± 25,8 | 485,7 ± 26,4 | |
| 203228_at | | platelet-activating factor acetylhydrolase, isoform Ib, subunit 3 (29kDa) | PAFAH1B3* | -13,51 | | 274,4 ± 47,4 | 30,2 ± 17 | | 209,8 ± 4,3 | 237,7 ± 27,9 | |
| 213661_at | | peptidase domain containing associated with muscle regeneration 1 | PAMR1 | -31,51 | | 1962,2 ± 142,8 | 79,3 ± 41,5 | | 1624,4 ± 9 | 3352,8 ± 69,1 | |
| 222273_at | | poly(A) polymerase gamma | PAPOLG | -3,85 | | 93,7 ± 31,7 | 26,1 ± 20,7 | | 94,4 ± 12,2 | 66,7 ± 27,1 | |
| 224941_at | | pregnancy-associated plasma protein A, pappalysin 1 | PAPPA | -2,74 | | 2067,6 ± 713,3 | 793 ± 323,9 | | 1734,6 ± 145,6 | 1388,1 ± 170,9 | |
| 227626_at | | progestin and adipoQ receptor family member VIII | PAQR8* | -2,74 | | 138,8 ± 27,8 | 66,6 ± 22,1 | | 161,3 ± 34,8 | 197,9 ± 30,7 | |
| 224701_at | | poly (ADP-ribose) polymerase family, member 14 | PARP14 | -4,70 | | 115,8 ± 17,9 | 37 ± 31,2 | | 190,1 ± 12,9 | 103,9 ± 21,7 | |
| 223220_s_at | | poly (ADP-ribose) polymerase family, member 9 | PARP9 | -2,70 | | 285,3 ± 11,3 | 130,1 ± 63,4 | | 380,7 ± 16,2 | 353,1 ± 35,8 | |
| 212406_s_at | | protein-L-isoaspartate (D-aspartate) O-methyltransferase domain containing 2 | PCMTD2* | -3,01 | | 481,2 ± 170,2 | 162,9 ± 82 | | 596,2 ± 10,7 | 359,6 ± 36,6 | |
| 203131_at | | platelet-derived growth factor receptor, alpha polypeptide | PDGFRA* | -8,84 | | 9660 ± 607,4 | 2376,8 ± 2544,1 | | 7120,3 ± 415,3 | 8916,9 ± 155,2 | |
| 228618_at | | platelet endothelial aggregation receptor 1 | PEAR1 | -7,88 | | 351,5 ± 94,3 | 64 ± 44,3 | | 305,5 ± 26,6 | 316,6 ± 30,2 | |
| 209422_at | | PHD finger protein 20 | PHF20 | -2,03 | | 809,7 ± 148,1 | 360,1 ± 132,3 | | 841 ± 11,7 | 734,2 ± 34,9 | |
| 202732_at | | protein kinase (cAMP-dependent, catalytic) inhibitor gamma | PKIG | -2,09 | | 1885,2 ± 428,2 | 932,2 ± 32,6 | | 2229,5 ± 128,2 | 2756,9 ± 129,6 | |
| 222662_at | | protein phosphatase 1, regulatory (inhibitor) subunit 3B | PPP1R3B | -3,62 | | 322,2 ± 25,6 | 107,4 ± 41,8 | | 462,4 ± 11,7 | 571 ± 35,1 | |
| 202429_s_at | | protein phosphatase 3 (formerly 2B), catalytic subunit, alpha isoform | PPP3CA | -2,58 | | 1761,9 ± 252,7 | 725,8 ± 409,2 | | 1744,8 ± 96,8 | 1330,8 ± 78,1 | |
| 203650_at | | protein C receptor, endothelial (EPCR) | PROCR | -4,32 | | 789,8 ± 119,8 | 234,5 ± 182,7 | | 715 ± 45,4 | 1056,9 ± 141,9 | |
| 226695_at | | paired related homeobox 1 | PRRX1 | -10,97 | | 4382,3 ± 359,2 | 1144,6 ± 1656,6 | | 2818 ± 129,2 | 2788,5 ± 57,3 | |
| 222803_at | | phosphoribosyl transferase domain containing 1 | PRTFDC1 | -4,29 | | 266 ± 146,6 | 87,3 ± 64,4 | | 350,2 ± 22,8 | 391,8 ± 24,1 | |
| 218613_at | | pleckstrin and Sec7 domain containing 3 | PSD3 | -6,86 | | 1379,1 ± 158,5 | 255 ± 162,2 | | 903,5 ± 66,5 | 921 ± 190,6 | |
| 207177_at | | prostaglandin F receptor (FP) | PTGFR | -9,55 | | 1220,7 ± 728,1 | 73,9 ± 26,8 | | 1630,7 ± 73,5 | 490,9 ± 29,3 | |
| 203038_at | | protein tyrosine phosphatase, receptor type, K | PTPRK | -4,39 | | 1636,8 ± 316,4 | 477,5 ± 409,4 | | 1292,4 ± 56,6 | 1037,6 ± 145,7 | |
| 221666_s_at | | PYD and CARD domain containing | PYCARD | -4,85 | | 601,6 ± 159,2 | 131,3 ± 28,6 | | 518,5 ± 65 | 819,7 ± 63,3 | |
| 228708_at | | RAB27B, member RAS oncogene family | RAB27B* | -39,40 | | 4122,8 ± 376,9 | 180 ± 224,4 | | 2848,2 ± 221,6 | 1700,7 ± 54,4 | |
| 218700_s_at | | RAB7, member RAS oncogene family-like 1 | RAB7L1* | -2,44 | | 286,5 ± 80,9 | 104,1 ± 21,6 | | 381,9 ± 25,6 | 414,5 ± 33,6 | |
| 213603_s_at | | ras-related C3 botulinum toxin substrate 2 (rho family, small GTP binding protein Rac2) | RAC2* | -5,83 | | 710 ± 124,3 | 133,6 ± 38,7 | | 1099,1 ± 57,8 | 1251,1 ± 108,9 | |
| 205130_at | | renal tumor antigen | RAGE | -2,08 | | 252 ± 43,4 | 129,3 ± 3,6 | | 221,2 ± 15,7 | 234,6 ± 15,7 | |
| 206392_s_at | | retinoic acid receptor responder (tazarotene induced) 1 | RARRES1 | -93,34 | | 1010,2 ± 592,5 | 10 ± 7,6 | | 556,8 ± 68,1 | 489,7 ± 71,6 | |
| 204070_at | | retinoic acid receptor responder (tazarotene induced) 3 | RARRES3 | -4,00 | | 554,7 ± 227,3 | 145,9 ± 67,3 | | 1296,8 ± 135,5 | 1099,3 ± 99,3 | |
| 228304_at | | RNA binding motif protein 43 | RBM43 | -3,43 | | 363,8 ± 61,1 | 119,6 ± 71,7 | | 511,4 ± 30 | 306 ± 27,4 | |
| 216215_s_at | | RNA binding motif protein 9 | RBM9 | -2,52 | | 1067 ± 81,8 | 408,6 ± 137,1 | | 1014,8 ± 110,7 | 1216,3 ± 209,4 | |
| 218352_at | | regulator of chromosome condensation (RCC1) and BTB (POZ) domain containing protein 1 | RCBTB1 | -2,09 | | 353,1 ± 53,3 | 184,8 ± 32,5 | | 520,1 ± 76,3 | 364,6 ± 12,6 | |
| 204319_s_at | | regulator of G-protein signaling 10 | RGS10* | -2,33 | | 608,7 ± 107,9 | 278,7 ± 126,7 | | 749,1 ± 10,2 | 462 ± 52,5 | |
| 219056_at | | ribonuclease H2, subunit B | RNASEH2B | -2,60 | | 128,3 ± 20 | 50,8 ± 18,1 | | 128,4 ± 1 | 114,3 ± 14,9 | |
| 225929_s_at | | ring finger protein 213 | RNF213 | -2,03 | | 186,8 ± 29,5 | 83,2 ± 6,5 | | 199,8 ± 51,1 | 202,2 ± 24,4 | |
| 213194_at | | roundabout, axon guidance receptor, homolog 1 (Drosophila) | ROBO1 | -2,58 | | 795,2 ± 206,2 | 280,4 ± 143,1 | | 822,3 ± 104 | 759,3 ± 28,9 | |
| 221593_s_at | | ribosomal protein L31 | RPL31 | -2,03 | | 456,5 ± 97,8 | 232,6 ± 53,2 | | 534 ± 13,1 | 359,7 ± 27,6 | |
| 224763_at | | Ribosomal protein L37 | RPL37 | -2,32 | | 466,3 ± 122,5 | 199,6 ± 5,5 | | 583,7 ± 33,8 | 407,3 ± 39,9 | |
| 219684_at | | receptor (chemosensory) transporter protein 4 | RTP4 | -10,31 | | 97,3 ± 20,8 | 14,5 ± 16,6 | | 126,8 ± 10 | 95,1 ± 30,9 | |
| 209360_s_at | | runt-related transcription factor 1 | RUNX1 | -5,04 | | 779 ± 292,6 | 179,9 ± 115,6 | | 1091 ± 124,4 | 805,7 ± 66,1 | |
| 203408_s_at | | SATB homeobox 1 | SATB1 | -2,70 | | 492,6 ± 106,1 | 192,1 ± 44,9 | | 596,6 ± 5,2 | 513,5 ± 46,3 | |
| 213435_at | | SATB homeobox 2 | SATB2 | -2,03 | | 515,3 ± 189 | 225,5 ± 16,4 | | 454,9 ± 13,7 | 435 ± 26,3 | |
| 229057_at | | sodium channel, voltage-gated, type II, alpha subunit | SCN2A | -27,01 | | 143,8 ± 54 | 17,2 ± 22 | | 186,2 ± 10,7 | 148 ± 20,6 | |
| 206805_at | | sema domain, immunoglobulin domain (Ig), short basic domain, secreted, (semaphorin) 3A | SEMA3A | -3,70 | | 156,7 ± 28,9 | 36,9 ± 14,2 | | 82,6 ± 7 | 95,9 ± 6,4 | |
| 203789_s_at | | sema domain, immunoglobulin domain (Ig), short basic domain, secreted, (semaphorin) 3C | SEMA3C | -5,74 | | 1862,7 ± 731 | 444,6 ± 378,9 | | 1182,3 ± 81,8 | 972,2 ± 97,1 | |
| 205405_at | | sema domain, seven thrombospondin repeats (type 1 and type 1-like), transmembrane domain (TM) and short cytoplasmic domain, (semaphorin) 5A | SEMA5A | -5,83 | | 211,3 ± 29,2 | 68,3 ± 51,6 | | 201,5 ± 16,5 | 137,4 ± 8,4 | |
| 204051_s_at | | secreted frizzled-related protein 4 | SFRP4 | -7,46 | | 1103,4 ± 943 | 114,9 ± 38,5 | | 8392,9 ± 438,7 | 1797,9 ± 365,1 | |
| 213649_at | | splicing factor, arginine/serine-rich 7, 35kDa | SFRS7 | -2,21 | | 354 ± 46,6 | 192,1 ± 69,7 | | 431,5 ± 13,3 | 413,2 ± 49,1 | |
| 223672_at | | SH3-domain GRB2-like (endophilin) interacting protein 1 | SGIP1 | -19,55 | | 32,1 ± 5,1 | 1,6 ± 1,7 | | 15,8 ± 9 | 21,8 ± 5,8 | |
| 230494_at | | Solute carrier family 20 (phosphate transporter), member 1 | SLC20A1 | -7,94 | | 161,3 ± 52,2 | 25,7 ± 28,9 | | 95 ± 10,2 | 115,8 ± 14,6 | |
| 202499_s_at | | solute carrier family 2 (facilitated glucose transporter), member 3 | SLC2A3 | -4,63 | | 424,9 ± 74,9 | 127,8 ± 96,3 | | 360,9 ± 15,4 | 478,7 ± 54,9 | |
| 213113_s_at | | solute carrier family 43, member 3 | SLC43A3 | -3,10 | | 469,9 ± 217,7 | 123,1 ± 59,2 | | 646,1 ± 25 | 671,1 ± 5,1 | |
| 203908_at | | solute carrier family 4, sodium bicarbonate cotransporter, member 4 | SLC4A4 | -7,41 | | 528 ± 11 | 98,9 ± 78,5 | | 335,4 ± 12,8 | 265,4 ± 15 | |
| 235518_at | | solute carrier family 8 (sodium/calcium exchanger), member 1 | SLC8A1 | -9,62 | | 92,2 ± 28,9 | 16,3 ± 14,9 | | 94,8 ± 10,8 | 62,2 ± 11 | |
| 231718_at | | SLU7 splicing factor homolog (S. cerevisiae) | SLU7 | -2,62 | | 298,2 ± 43 | 159,1 ± 73,4 | | 302,4 ± 46,6 | 231,4 ± 12,1 | |
| 218284_at | | SMAD family member 3 | SMAD3 | -3,54 | | 454,7 ± 23,1 | 165,5 ± 48,2 | | 467,6 ± 21 | 582,8 ± 23,9 | |
| 206544_x_at | | SWI/SNF related, matrix associated, actin dependent regulator of chromatin, subfamily a, member 2 | SMARCA2 | -3,48 | | 437,7 ± 36,4 | 161,9 ± 125,4 | | 351 ± 25,3 | 421,9 ± 32 | |
| 226685_at | | syntrophin, beta 2 (dystrophin-associated protein A1, 59kDa, basic component 2) | SNTB2 | -2,08 | | 1406,2 ± 132,4 | 750,6 ± 299 | | 1507,6 ± 69,4 | 1923,4 ± 151 | |
| 223027_at | | sorting nexin 9 | SNX9* | -3,03 | | 1807,7 ± 800,1 | 519,6 ± 169,6 | | 2010,7 ± 103,6 | 1615 ± 87,9 | |
| 227697_at | | suppressor of cytokine signaling 3 | SOCS3* | -3,97 | | 1206 ± 271,7 | 301,8 ± 95,6 | | 1181,6 ± 37,3 | 1801,7 ± 108,8 | |
| 209647_s_at | | suppressor of cytokine signaling 5 | SOCS5 | -6,50 | | 1357,7 ± 83,7 | 275,6 ± 267,3 | | 1030,7 ± 102,3 | 1077,6 ± 31,6 | |
| 201086_x_at | | SON DNA binding protein | SON | -2,14 | | 1293,7 ± 134,5 | 669,9 ± 406 | | 1195,1 ± 89,6 | 1160,4 ± 7,3 | |
| 229331_at | | spermatogenesis associated 18 homolog (rat) | SPATA18 | -2,79 | | 566,3 ± 154,9 | 214,7 ± 82,6 | | 627,7 ± 71,1 | 629,9 ± 54,5 | |
| 234929_s_at | | spermatogenesis associated 7 | SPATA7 | -2,66 | | 153,7 ± 22 | 57,8 ± 19,4 | | 135,2 ± 35,2 | 122,5 ± 27,8 | |
| 222431_at | | spindlin 1 | SPIN1 | -2,76 | | 2132,4 ± 156,6 | 806,9 ± 391,3 | | 1799,5 ± 43,6 | 1882,7 ± 33,7 | |
| 228654_at | | spindlin family, member 4 | SPIN4 | -4,13 | | 231,1 ± 48,5 | 66,2 ± 46,1 | | 155 ± 25 | 167,7 ± 8 | |
| 202363_at | | sparc/osteonectin, cwcv and kazal-like domains proteoglycan (testican) 1 | SPOCK1 | -5,28 | | 3096,8 ± 662,3 | 770 ± 591,1 | | 3024,9 ± 220,8 | 2972,1 ± 52,3 | |
| 227752_at | | serine palmitoyltransferase, long chain base subunit 3 | SPTLC3* | -14,48 | | 140,8 ± 18,4 | 14,6 ± 14 | | 124,4 ± 14,9 | 181,9 ± 31,1 | |
| 227484_at | | SLIT-ROBO Rho GTPase activating protein 1 | SRGAP1 | -2,79 | | 255,9 ± 65,7 | 86,2 ± 20,1 | | 278,9 ± 38 | 266,7 ± 50,2 | |
| 224974_at | | suppressor of defective silencing 3 homolog (S. cerevisiae) | SUDS3 | -2,54 | | 260 ± 55,4 | 114,9 ± 52,5 | | 272,6 ± 11,9 | 334,2 ± 12,1 | |
| 212354_at | | sulfatase 1 | SULF1 | -44,56 | | 1840,5 ± 485,8 | 175,1 ± 266,1 | | 1047,5 ± 89 | 635,6 ± 33,7 | |
| 210612_s_at | | synaptojanin 2 | SYNJ2* | -80,63 | | 683,6 ± 174,5 | 31,7 ± 51,7 | | 397,6 ± 31,7 | 633,9 ± 64,4 | |
| 226388_at | | transcription elongation factor A (SII), 3 | TCEA3 | -3,43 | | 299,9 ± 80,4 | 89,2 ± 42,5 | | 323,5 ± 12,1 | 524,4 ± 33,4 | |
| 212387_at | | transcription factor 4 | TCF4 | -17,96 | | 605,2 ± 72,6 | 79,5 ± 96,3 | | 279,8 ± 13,7 | 265,7 ± 27,6 | |
| 224560_at | | TIMP metallopeptidase inhibitor 2 | TIMP2 | -2,54 | | 3776,3 ± 693,4 | 1439,9 ± 132,2 | | 4700 ± 355,9 | 5488,5 ± 143,1 | |
| 205122_at | | transmembrane protein with EGF-like and two follistatin-like domains 1 | TMEFF1 | -4,85 | | 211,1 ± 58,3 | 39,3 ± 18,7 | | 260,7 ± 49 | 143,4 ± 35,9 | |
| 234994_at | | transmembrane protein 200A | TMEM200A | -10,80 | | 2744,7 ± 845,8 | 359,8 ± 399,1 | | 1626,8 ± 73,6 | 2132,4 ± 133,3 | |
| 226338_at | | transmembrane protein 55A | TMEM55A | -2,54 | | 509,7 ± 28,5 | 225,5 ± 164,5 | | 496,3 ± 88,3 | 481,5 ± 121,1 | |
| 202687_s_at | | tumor necrosis factor (ligand) superfamily, member 10 | TNFSF10 | -5,57 | | 80,5 ± 22,9 | 14 ± 5,3 | | 104 ± 7,6 | 116,4 ± 17,2 | |
| 202561_at | | tankyrase, TRF1-interacting ankyrin-related ADP-ribose polymerase | TNKS | -2,68 | | 430,3 ± 55,7 | 177,6 ± 52,2 | | 379,9 ± 35,8 | 447,6 ± 116,5 | |
| 200662_s_at | | translocase of outer mitochondrial membrane 20 homolog (yeast) | TOMM20 | -2,11 | | 4750,9 ± 267,5 | 2393,7 ± 997,2 | | 4350,4 ± 200,9 | 3641,5 ± 165 | |
| 204529_s_at | | thymocyte selection-associated high mobility group box | TOX | -4,13 | | 58,3 ± 2,8 | 13,1 ± 8,5 | | 109,2 ± 19,1 | 46,4 ± 2,2 | |
| 202080_s_at | | trafficking protein, kinesin binding 1 | TRAK1 | -2,08 | | 504,4 ± 160,8 | 227,8 ± 19,9 | | 673,5 ± 19,7 | 498,5 ± 36 | |
| 238520_at | | transcriptional regulating factor 1 | TRERF1* | -11,31 | | 118,2 ± 23,5 | 12 ± 6,3 | | 104,2 ± 11,9 | 147,4 ± 9,6 | |
| 221044_s_at | | tripartite motif-containing 34 /// TRIM6-TRIM34 readthrough transcript | TRIM34 /// TRIM6-TRIM34 | -4,42 | | 210,8 ± 26,1 | 72,3 ± 53,7 | | 219,3 ± 9,3 | 314,7 ± 21,6 | |
| 227610_at | | tetraspanin 11 | TSPAN11 | -6,35 | | 276,3 ± 139,3 | 58,7 ± 24,3 | | 198,2 ± 23,2 | 261,1 ± 22,7 | |
| 214983_at | | testis-specific transcript, Y-linked 15 (non-protein coding) | TTTY15 | -4,00 | | 94 ± 40 | 24,3 ± 16,4 | | 60,6 ± 6,5 | 67,3 ± 13 | |
| 208884_s_at | | ubiquitin protein ligase E3 component n-recognin 5 | UBR5 | -2,24 | | 687,1 ± 109,1 | 363,2 ± 157 | | 664,4 ± 45 | 574 ± 42 | |
| 217100_s_at | | UBX domain protein 7 | UBXN7 | -3,01 | | 127 ± 9,9 | 52,2 ± 16,8 | | 131,3 ± 9,9 | 110,8 ± 23,8 | |
| 225655_at | | ubiquitin-like with PHD and ring finger domains 1 | UHRF1 | -15,88 | | 353,2 ± 176 | 55,2 ± 86,7 | | 135,3 ± 9 | 132,7 ± 15 | |
| 204254_s_at | | vitamin D (1,25- dihydroxyvitamin D3) receptor | VDR | -3,56 | | 349,3 ± 42,9 | 92,5 ± 49,7 | | 351,9 ± 35 | 366,1 ± 12,6 | |
| 209946_at | | vascular endothelial growth factor C | VEGFC | -2,58 | | 1118,9 ± 76 | 406,3 ± 155,8 | | 992,6 ± 42,7 | 1072,6 ± 56,5 | |
| 202172_at | | vascular endothelial zinc finger 1 | VEZF1 | -2,64 | | 482,9 ± 51,6 | 180,4 ± 81,2 | | 418,5 ± 17,9 | 454,7 ± 5,8 | |
| 235625_at | | vacuolar protein sorting 41 homolog (S. cerevisiae) | VPS41 | -6,11 | | 299,6 ± 45 | 112,4 ± 104,8 | | 393,6 ± 48,3 | 239,9 ± 37,4 | |
| 205792_at | | WNT1 inducible signaling pathway protein 2 | WISP2* | -33,00 | | 948,5 ± 460,4 | 28,4 ± 21,9 | | 1091,6 ± 72,2 | 505,8 ± 48,1 | |
| 212455_at | | YTH domain containing 1 | YTHDC1 | -2,18 | | 785,9 ± 79,4 | 397,8 ± 237,6 | | 807,1 ± 89,1 | 641,6 ± 86,2 | |
| 224718_at | | YY1 transcription factor | YY1 | -2,32 | | 511,9 ± 105,9 | 194,5 ± 32,5 | | 411,4 ± 30,4 | 472,3 ± 10,6 | |
| 226137_at | | zinc finger homeobox 3 | ZFHX3 | -2,08 | | 467,7 ± 180 | 234,5 ± 37,4 | | 415,4 ± 59,6 | 470,1 ± 72 | |
| 214670_at | | zinc finger with KRAB and SCAN domains 1 | ZKSCAN1 | -2,35 | | 351,5 ± 40,6 | 196,1 ± 127,4 | | 416,1 ± 11,1 | 406,8 ± 14,7 | |
| 219628_at | | zinc finger, matrin type 3 | ZMAT3 | -2,41 | | 1138,6 ± 128,1 | 502 ± 44,7 | | 1168,3 ± 47,9 | 1052,9 ± 196,7 | |
| 217403_s_at | | zinc finger protein 227 | ZNF227 | -7,94 | | 79,8 ± 9,3 | 9,3 ± 6 | | 63,4 ± 8,2 | 44,8 ± 5 | |
| 227768_at | | zinc finger protein 407 | ZNF407 | -2,70 | | 183,6 ± 18,2 | 66,5 ± 37,1 | | 182,9 ± 35,7 | 140,3 ± 19,1 | |
| **Cluster 4 genes** | | | | | | | | | | | |
| 225663_at | | acyl-Coenzyme A binding domain containing 5 | ACBD5 | -2,18 | | 646 ± 49,9 | 337,6 ± 167,2 | | 719,5 ± 70 | 382,9 ± 54 | |
| 1555326_a_at | | ADAM metallopeptidase domain 9 (meltrin gamma) | ADAM9 | -11,49 | | 296,8 ± 84,8 | 48,2 ± 67,2 | | 203,9 ± 27,4 | 69,5 ± 7,4 | |
| 229357_at | | ADAM metallopeptidase with thrombospondin type 1 motif, 5 | ADAMTS5 | -22,11 | | 1085,4 ± 414,8 | 72,8 ± 53,5 | | 294,3 ± 28,1 | 124,2 ± 10,7 | |
| 225889_at | | AE binding protein 2 | AEBP2 | -2,54 | | 397,1 ± 9,9 | 181,2 ± 35,6 | | 433,4 ± 56,5 | 224,2 ± 21,6 | |
| 225229_at | | AF4/FMR2 family, member 4 | AFF4 | -2,60 | | 1100,6 ± 207 | 467,7 ± 152,1 | | 1122,4 ± 81,8 | 607,7 ± 119,2 | |
| 232007_at | | 1-acylglycerol-3-phosphate O-acyltransferase 5 (lysophosphatidic acid acyltransferase, epsilon) | AGPAT5 | -3,17 | | 43,8 ± 10,8 | 11,5 ± 2,1 | | 32,2 ± 6,7 | 19,9 ± 4,6 | |
| 203156_at | | A kinase (PRKA) anchor protein 11 | AKAP11 | -3,03 | | 1090,6 ± 87,6 | 379,1 ± 165,8 | | 850,9 ± 58 | 320,5 ± 38,7 | |
| 226421_at | | Alport syndrome, mental retardation, midface hypoplasia and elliptocytosis chromosomal region gene 1 | AMMECR1 | -2,19 | | 160,4 ± 43,5 | 66,7 ± 15,9 | | 77,7 ± 15,5 | 80 ± 4,8 | |
| 224682_at | | ankyrin repeat and IBR domain containing 1 | ANKIB1 | -2,48 | | 784,2 ± 58,7 | 400,4 ± 221,6 | | 905 ± 96,1 | 459,5 ± 35,9 | |
| 225731_at | | ankyrin repeat domain 50 | ANKRD50 | -2,83 | | 485,5 ± 6,2 | 187,8 ± 93,2 | | 444 ± 28,7 | 129,2 ± 13,6 | |
| 218158_s_at | | adaptor protein, phosphotyrosine interaction, PH domain and leucine zipper containing 1 | APPL1 | -2,46 | | 227 ± 30,5 | 98,6 ± 34,7 | | 294,7 ± 36,1 | 110 ± 13,8 | |
| 201334_s_at | | Rho guanine nucleotide exchange factor (GEF) 12 | ARHGEF12 | -3,15 | | 1198,4 ± 75,9 | 467,3 ± 180,4 | | 770,4 ± 152,3 | 717,3 ± 130,9 | |
| 221230_s_at | | AT rich interactive domain 4B (RBP1-like) | ARID4B | -3,17 | | 427,1 ± 147,4 | 146,7 ± 62,9 | | 506,5 ± 44,9 | 122,3 ± 27,6 | |
| 212474_at | | AVL9 homolog (S. cerevisiase) | AVL9 | -2,11 | | 445,3 ± 47,3 | 221,2 ± 14,3 | | 516,9 ± 21,6 | 332,4 ± 42,7 | |
| 204194_at | | BTB and CNC homology 1, basic leucine zipper transcription factor 1 | BACH1 | -3,76 | | 233,8 ± 53 | 68 ± 54,1 | | 247,3 ± 52,3 | 72,6 ± 16,6 | |
| 211946_s_at | | BAT2 domain containing 1 | BAT2D1 | -2,42 | | 1226,9 ± 80,9 | 679,1 ± 334,3 | | 1166,2 ± 185,9 | 680,9 ± 26,5 | |
| 225285_at | | branched chain aminotransferase 1, cytosolic | BCAT1 | -3,82 | | 862 ± 20,4 | 276,5 ± 248,1 | | 561,7 ± 59,2 | 183,3 ± 23,7 | |
| 209271_at | | bromodomain PHD finger transcription factor | BPTF | -2,62 | | 348,8 ± 54 | 139,7 ± 68,6 | | 387,9 ± 69,6 | 186,2 ± 22,2 | |
| 207483_s_at | | cullin-associated and neddylation-dissociated 1 | CAND1 | -2,35 | | 664,9 ± 58,5 | 307,3 ± 168,3 | | 724,5 ± 61,5 | 382,7 ± 54,2 | |
| 220301_at | | coiled-coil domain containing 102B | CCDC102B | -9,70 | | 101,1 ± 29,8 | 21,1 ± 23 | | 118,5 ± 28,5 | 45,9 ± 15,1 | |
| 217523_at | | CD44 molecule (Indian blood group) | CD44 | -5,83 | | 119,6 ± 63,7 | 15,4 ± 10,6 | | 99,6 ± 9,1 | 35 ± 10,2 | |
| 212899_at | | cell division cycle 2-like 6 (CDK8-like) | CDC2L6 | -2,19 | | 381,1 ± 24,6 | 188,7 ± 37,6 | | 381,2 ± 11 | 264,2 ± 31,6 | |
| 212746_s_at | | centrosomal protein 170kDa | CEP170 | -7,46 | | 567,1 ± 177,7 | 88,2 ± 91,5 | | 401 ± 54 | 193,3 ± 44,6 | |
| 218827_s_at | | centrosomal protein 192kDa | CEP192 | -2,14 | | 136,8 ± 8,4 | 65,6 ± 37,7 | | 123 ± 30,6 | 54,2 ± 7,8 | |
| 203494_s_at | | centrosomal protein 57kDa | CEP57 | -2,83 | | 439,2 ± 61,4 | 151,1 ± 46,1 | | 589,9 ± 33,4 | 154,2 ± 13,3 | |
| 224599_at | | CGG triplet repeat binding protein 1 | CGGBP1 | -2,42 | | 1210,2 ± 59,2 | 546,3 ± 291,7 | | 1324,6 ± 114,3 | 480,9 ± 111 | |
| 228345_at | | cysteine-rich hydrophobic domain 1 | CHIC1 | -4,39 | | 179,8 ± 24,6 | 53,8 ± 45,7 | | 196,2 ± 15,8 | 63,7 ± 19,8 | |
| 205830_at | | calmegin | CLGN | -4,92 | | 289,7 ± 149,9 | 74,6 ± 84,6 | | 101,8 ± 17,8 | 115,9 ± 8,6 | |
| 226939_at | | cytoplasmic polyadenylation element binding protein 2 | CPEB2 | -2,13 | | 1087,4 ± 145,9 | 506,4 ± 73,3 | | 887,8 ± 56,3 | 675 ± 63,1 | |
| 228365_at | | copine VIII | CPNE8 | -2,32 | | 135,8 ± 17,1 | 78,5 ± 26,3 | | 177 ± 14,6 | 74,8 ± 7,3 | |
| 218871_x_at | | chondroitin sulfate N-acetylgalactosaminyltransferase 2 | CSGALNACT2 | -2,24 | | 870,9 ± 25,2 | 435,3 ± 229,8 | | 702,6 ± 67 | 467,4 ± 12 | |
| 222235_s_at | | chondroitin sulfate N-acetylgalactosaminyltransferase 2 /// novel protein similar to chondroitin sulfate GalNAcT-2 (GALNACT-2) | CSGALNACT2 /// RP1-19N1.1 | -2,89 | | 956,3 ± 77,8 | 337 ± 153,7 | | 709,8 ± 64,4 | 519,2 ± 28,4 | |
| 218923_at | | chitobiase, di-N-acetyl- | CTBS | -2,19 | | 260,2 ± 48,5 | 131,4 ± 84 | | 243,7 ± 43,8 | 147,1 ± 27 | |
| 202468_s_at | | catenin (cadherin-associated protein), alpha-like 1 | CTNNAL1 | -4,35 | | 2195,3 ± 254,5 | 742 ± 692,6 | | 3125,9 ± 21,5 | 1166,7 ± 82 | |
| 210257_x_at | | cullin 4B | CUL4B | -4,09 | | 370 ± 78,5 | 118,2 ± 61,7 | | 253 ± 20,8 | 147,4 ± 11,5 | |
| 203302_at | | deoxycytidine kinase | DCK | -2,48 | | 407,9 ± 42,8 | 174,5 ± 114,3 | | 302,1 ± 12,6 | 263,1 ± 25,4 | |
| 225442_at | | discoidin domain receptor tyrosine kinase 2 | DDR2 | -3,76 | | 3038,1 ± 308,4 | 1109,5 ± 985,8 | | 3918,7 ± 123,7 | 1717,4 ± 316,8 | |
| 205000_at | | DEAD (Asp-Glu-Ala-Asp) box polypeptide 3, Y-linked | DDX3Y | -10,64 | | 724,2 ± 182,3 | 187,2 ± 185,8 | | 629,5 ± 48,8 | 354,3 ± 32,2 | |
| 218986_s_at | | DEAD (Asp-Glu-Ala-Asp) box polypeptide 60 | DDX60 | -3,59 | | 129,1 ± 36,7 | 36,5 ± 18,5 | | 213,9 ± 38,5 | 52,2 ± 13,7 | |
| 200934_at | | DEK oncogene | DEK | -2,54 | | 895,6 ± 114,1 | 382,2 ± 161,9 | | 952,8 ± 126,8 | 192,6 ± 3,7 | |
| 228551_at | | DENN/MADD domain containing 5B | DENND5B | -2,30 | | 194,7 ± 16,2 | 97,9 ± 11,6 | | 223 ± 32,2 | 92,1 ± 24,9 | |
| 229588_at | | DnaJ (Hsp40) homolog, subfamily C, member 10 | DNAJC10 | -4,67 | | 88,6 ± 17,1 | 27,7 ± 20,1 | | 74,3 ± 18 | 31,1 ± 3,1 | |
| 212467_at | | DnaJ (Hsp40) homolog, subfamily C, member 13 | DNAJC13 | -3,01 | | 514,1 ± 7,9 | 245,6 ± 190,1 | | 604,8 ± 90,2 | 211,6 ± 17,5 | |
| 230893_at | | DnaJ (Hsp40) homolog, subfamily C, member 21 | DNAJC21 | -3,88 | | 101 ± 25 | 35,5 ± 18,6 | | 118,7 ± 28,2 | 44,2 ± 9,2 | |
| 212253_x_at | | dystonin | DST | -14,25 | | 153,7 ± 51,6 | 33,2 ± 52,6 | | 172,1 ± 17,2 | 12 ± 4 | |
| 236649_at | | DTW domain containing 1 | DTWD1 | -6,86 | | 251 ± 66,3 | 68,6 ± 81,9 | | 386,1 ± 17,7 | 158,9 ± 12,3 | |
| 219469_at | | dynein, cytoplasmic 2, heavy chain 1 | DYNC2H1 | -3,73 | | 180,6 ± 20,5 | 47,4 ± 47,2 | | 220,2 ± 51,4 | 54,5 ± 11,8 | |
| 204464_s_at | | endothelin receptor type A | EDNRA | -5,92 | | 720,4 ± 169,1 | 171,8 ± 129,3 | | 266,8 ± 10,2 | 237,5 ± 41 | |
| 226800_at | | EF-hand calcium binding domain 7 | EFCAB7 | -3,13 | | 209,7 ± 40,2 | 69,7 ± 27,3 | | 304,5 ± 18,4 | 104,1 ± 14,4 | |
| 219532_at | | elongation of very long chain fatty acids (FEN1/Elo2, SUR4/Elo3, yeast)-like 4 | ELOVL4 | -2,54 | | 145,4 ± 43,2 | 51,5 ± 13,8 | | 168,1 ± 12 | 95,8 ± 4,1 | |
| 226789_at | | embigin homolog (mouse) | EMB | -10,56 | | 397,5 ± 184,2 | 36,8 ± 28,9 | | 182,3 ± 16,8 | 102,8 ± 17,1 | |
| 223068_at | | echinoderm microtubule associated protein like 4 | EML4 | -3,56 | | 735,3 ± 111,8 | 296,8 ± 214,8 | | 876,9 ± 58,8 | 319,3 ± 22 | |
| 204774_at | | ecotropic viral integration site 2A | EVI2A | -9,85 | | 215,5 ± 130,9 | 25,3 ± 24,3 | | 3086,1 ± 248,2 | 52,7 ± 12,2 | |
| 230183_at | | exostoses (multiple) 1 | EXT1 | -4,92 | | 273,9 ± 28,1 | 80,4 ± 63,7 | | 324,2 ± 30,8 | 155,8 ± 21,5 | |
| 226568_at | | family with sequence similarity 102, member B | FAM102B | -2,23 | | 229,2 ± 47,8 | 118,6 ± 37,7 | | 209,9 ± 29 | 150,7 ± 32,3 | |
| 241808_at | | family with sequence similarity 164, member A | FAM164A | -3,08 | | 149,4 ± 17,8 | 48,1 ± 28,8 | | 158,3 ± 17,9 | 31,2 ± 6,1 | |
| 203482_at | | family with sequence similarity 178, member A | FAM178A | -3,85 | | 183,5 ± 7,4 | 43,5 ± 8,3 | | 152,1 ± 6,6 | 62,5 ± 11,4 | |
| 212929_s_at | | family with sequence similarity 21, member A /// family with sequence similarity 21, member B /// family with sequence similarity 21, member C /// family with sequence similarity 21, member D | FAM21A /// FAM21B /// FAM21C /// FAM21D | -2,60 | | 885 ± 47,2 | 348,7 ± 166,6 | | 972,4 ± 42,1 | 571 ± 34,4 | |
| 225667_s_at | | family with sequence similarity 84, member A | FAM84A | -4,46 | | 180,8 ± 55,8 | 46,8 ± 42,8 | | 328,7 ± 22,1 | 70,1 ± 10,4 | |
| 226294_x_at | | family with sequence similarity 91, member A1 | FAM91A1 | -2,60 | | 626,1 ± 61,3 | 224,4 ± 70,8 | | 589,7 ± 27 | 266,3 ± 55,3 | |
| 209955_s_at | | fibroblast activation protein, alpha | FAP | -10,97 | | 1593,4 ± 426,4 | 146,9 ± 111,1 | | 1805,1 ± 165,2 | 436,7 ± 44,8 | |
| 203184_at | | fibrillin 2 | FBN2 | -2,87 | | 493,3 ± 51,2 | 163,3 ± 58,4 | | 250,4 ± 17,6 | 166,8 ± 13,9 | |
| 228220_at | | FCH domain only 2 | FCHO2 | -2,21 | | 412,3 ± 38,3 | 158,3 ± 46,7 | | 448,4 ± 39,6 | 108,6 ± 7,4 | |
| 204359_at | | fibronectin leucine rich transmembrane protein 2 | FLRT2 | -8,06 | | 1091,2 ± 396,5 | 144,8 ± 94,3 | | 718 ± 73,4 | 382,7 ± 37,9 | |
| 228768_at | | folliculin interacting protein 1 | FNIP1 | -2,28 | | 411,8 ± 76,7 | 214,9 ± 43,9 | | 557,9 ± 196,6 | 222,5 ± 69,1 | |
| 225481_at | | FERM domain containing 6 | FRMD6 | -5,00 | | 3435,8 ± 664,4 | 1097,4 ± 1271,7 | | 4576,7 ± 61,2 | 1862,7 ± 268,6 | |
| 201637_s_at | | fragile X mental retardation, autosomal homolog 1 | FXR1 | -2,39 | | 1824,3 ± 231,3 | 904,4 ± 515,1 | | 2228,5 ± 62,5 | 889,1 ± 60,2 | |
| 237183_at | | UDP-N-acetyl-alpha-D-galactosamine:polypeptide N-acetylgalactosaminyltransferase 5 (GalNAc-T5) | GALNT5 | -4,81 | | 105 ± 14,4 | 24,5 ± 20,8 | | 63,4 ± 2 | 43,8 ± 6,9 | |
| 214085_x_at | | GLI pathogenesis-related 1 | GLIPR1 | -23,16 | | 3263,9 ± 1090,6 | 586,6 ± 760,5 | | 3366,2 ± 125,4 | 1319,8 ± 86,8 | |
| 212959_s_at | | N-acetylglucosamine-1-phosphate transferase, alpha and beta subunits | GNPTAB | -2,89 | | 538,5 ± 46,4 | 217,9 ± 63,3 | | 838,1 ± 76,6 | 303,9 ± 41,2 | |
| 219313_at | | GRAM domain containing 1C | GRAMD1C | -32,00 | | 42,1 ± 10 | 2 ± 2,3 | | 40,1 ± 9,1 | 10,3 ± 4,4 | |
| 222685_at | | HAUS augmin-like complex, subunit 6 | HAUS6 | -2,42 | | 239,3 ± 15,3 | 105,9 ± 62,9 | | 231,8 ± 28,2 | 35,5 ± 4,7 | |
| 209314_s_at | | HBS1-like (S. cerevisiae) | HBS1L | -2,03 | | 493,1 ± 53,2 | 234,8 ± 101,5 | | 360,2 ± 41,1 | 239,7 ± 33,9 | |
| 209526_s_at | | hepatoma-derived growth factor, related protein 3 | HDGFRP3 | -3,08 | | 591,8 ± 66,4 | 243,1 ± 142,6 | | 609,5 ± 44,5 | 363 ± 30,4 | |
| 235944_at | | hemicentin 1 | HMCN1 | -10,80 | | 496,6 ± 204,4 | 75,1 ± 97,3 | | 369,2 ± 35,1 | 175,2 ± 27,9 | |
| 221919_at | | heterogeneous nuclear ribonucleoprotein A1 | HNRNPA1 | -3,73 | | 185,2 ± 22,3 | 51,8 ± 31 | | 189 ± 27,9 | 103,7 ± 17,9 | |
| 203284_s_at | | heparan sulfate 2-O-sulfotransferase 1 | HS2ST1 | -2,14 | | 312,6 ± 36,3 | 148,3 ± 43,9 | | 349,1 ± 30,8 | 107,8 ± 8,4 | |
| 1558956_s_at | | intraflagellar transport 80 homolog (Chlamydomonas) | IFT80 | -4,00 | | 744 ± 129,1 | 221,5 ± 166 | | 878,4 ± 130 | 173,7 ± 10,4 | |
| 206693_at | | interleukin 7 | IL7 | -11,31 | | 519,3 ± 137,7 | 66,3 ± 68,1 | | 608,7 ± 24,3 | 192 ± 25 | |
| 242979_at | | insulin receptor substrate 1 | IRS1 | -3,76 | | 377,5 ± 64,8 | 114 ± 44,8 | | 206,8 ± 27,7 | 192,4 ± 24,9 | |
| 226189_at | | integrin, beta 8 | ITGB8 | -12,90 | | 350,9 ± 117,4 | 18,9 ± 19,8 | | 293,4 ± 54,2 | 69,6 ± 23,7 | |
| 212943_at | | KIAA0528 | KIAA0528 | -3,51 | | 533,2 ± 75,3 | 146,2 ± 104,7 | | 679,7 ± 49,7 | 269,8 ± 9,3 | |
| 228446_at | | KIAA2026 | KIAA2026 | -2,83 | | 189,6 ± 33,6 | 72,5 ± 42,6 | | 236,1 ± 36,8 | 58,6 ± 4,8 | |
| 227261_at | | Kruppel-like factor 12 | KLF12 | -6,81 | | 520,1 ± 74,8 | 130,6 ± 109,8 | | 395 ± 16,6 | 173,6 ± 20,8 | |
| 226671_at | | lysosomal-associated membrane protein 2 | LAMP2 | -3,79 | | 512,8 ± 129,9 | 171,9 ± 144 | | 700,6 ± 65,5 | 175,9 ± 25,6 | |
| 208933_s_at | | lectin, galactoside-binding, soluble, 8 | LGALS8 | -5,16 | | 512,7 ± 39 | 181,9 ± 154,6 | | 659,3 ± 81,1 | 245,1 ± 43,6 | |
| 225575_at | | leukemia inhibitory factor receptor alpha | LIFR | -2,19 | | 434,1 ± 120,7 | 231,3 ± 52,8 | | 616,3 ± 134 | 202,5 ± 43,2 | |
| 222457_s_at | | LIM domain and actin binding 1 | LIMA1 | -3,05 | | 3357,2 ± 411,4 | 1392,9 ± 927,5 | | 4620,6 ± 130,9 | 2454,5 ± 78,8 | |
| 218589_at | | lysophosphatidic acid receptor 6 | LPAR6 | -3,73 | | 419,2 ± 21,2 | 167,7 ± 169,5 | | 426,7 ± 27 | 116 ± 9,5 | |
| 219338_s_at | | leucine rich repeat containing 49 | LRRC49 | -2,60 | | 186 ± 3,8 | 79,5 ± 34 | | 161,9 ± 13 | 100,2 ± 11,5 | |
| 212131_at | | LSM14A, SCD6 homolog A (S. cerevisiae) | LSM14A | -2,96 | | 757 ± 154,1 | 320,1 ± 207,4 | | 669,6 ± 14,8 | 449,9 ± 41,5 | |
| 208634_s_at | | microtubule-actin crosslinking factor 1 | MACF1 | -2,19 | | 1415 ± 208,5 | 663,6 ± 341 | | 1509,6 ± 72,2 | 1019,5 ± 130,5 | |
| 202350_s_at | | matrilin 2 | MATN2 | -3,03 | | 462,6 ± 162,3 | 160,1 ± 19,1 | | 565,8 ± 51 | 222,8 ± 24,8 | |
| 226420_at | | MDS1 and EVI1 complex locus | MECOM | -6,40 | | 101,7 ± 23 | 18,9 ± 18,1 | | 102,6 ± 16,7 | 48,3 ± 7,2 | |
| 209861_s_at | | methionyl aminopeptidase 2 | METAP2 | -2,56 | | 1610,8 ± 121,8 | 757,6 ± 475,5 | | 1599,1 ± 58,3 | 901,5 ± 29,7 | |
| 222447_at | | methyltransferase like 9 | METTL9 | -2,96 | | 337 ± 56,3 | 159,9 ± 83,3 | | 288,6 ± 18,5 | 221,6 ± 26 | |
| 225475_at | | mesoderm induction early response 1 homolog (Xenopus laevis) | MIER1 | -2,44 | | 398,4 ± 9,7 | 199,2 ± 98,8 | | 474,6 ± 41,2 | 129,7 ± 3,5 | |
| 228961_at | | mesoderm induction early response 1, family member 3 | MIER3 | -2,14 | | 180,6 ± 63,8 | 69,5 ± 26,3 | | 181,1 ± 22,1 | 52,1 ± 9,1 | |
| 206860_s_at | | missing oocyte, meiosis regulator, homolog (Drosophila) | MIOS | -2,39 | | 432,2 ± 95 | 216,1 ± 72,1 | | 226,8 ± 33,8 | 174,4 ± 11,2 | |
| 218259_at | | MKL/myocardin-like 2 | MKL2 | -2,44 | | 324,5 ± 90,9 | 167,9 ± 73,8 | | 350,8 ± 63,3 | 225 ± 34,8 | |
| 223614_at | | matrix metallopeptidase 16 (membrane-inserted) | MMP16 | -14,59 | | 498,6 ± 266,6 | 39,6 ± 24,6 | | 165,3 ± 13,2 | 96,8 ± 3,9 | |
| 209421_at | | mutS homolog 2, colon cancer, nonpolyposis type 1 (E. coli) | MSH2 | -3,15 | | 180,3 ± 24,5 | 62,3 ± 33 | | 184,5 ± 23,8 | 80,9 ± 7 | |
| 227761_at | | myosin VA (heavy chain 12, myoxin) | MYO5A | -6,20 | | 326,9 ± 77,6 | 80,2 ± 58,4 | | 187 ± 30,2 | 95,1 ± 10 | |
| 202259_s_at | | NEDD4 binding protein 2-like 2 | N4BP2L2 | -3,54 | | 233,8 ± 25,6 | 80,1 ± 57,6 | | 163,2 ± 8,6 | 64,9 ± 7,2 | |
| 208754_s_at | | nucleosome assembly protein 1-like 1 | NAP1L1 | -3,25 | | 1039,3 ± 191,1 | 377,2 ± 269 | | 1112,3 ± 87,6 | 670,1 ± 124,8 | |
| 225344_at | | nuclear receptor coactivator 7 | NCOA7 | -2,11 | | 1130,7 ± 103,7 | 520,9 ± 14,8 | | 1141,4 ± 91,2 | 794,2 ± 42,7 | |
| 224984_at | | nuclear factor of activated T-cells 5, tonicity-responsive | NFAT5 | -4,19 | | 917,2 ± 24,9 | 217,6 ± 162,6 | | 753,2 ± 28,6 | 460,7 ± 130,3 | |
| 223218_s_at | | nuclear factor of kappa light polypeptide gene enhancer in B-cells inhibitor, zeta | NFKBIZ | -4,70 | | 591,4 ± 104,5 | 166,1 ± 145,7 | | 441,5 ± 16 | 193,7 ± 10 | |
| 218129_s_at | | nuclear transcription factor Y, beta | NFYB | -3,56 | | 397,2 ± 218,4 | 110,1 ± 75,7 | | 494 ± 36,8 | 177,7 ± 8,8 | |
| 228933_at | | Nance-Horan syndrome (congenital cataracts and dental anomalies) | NHS | -5,20 | | 569,6 ± 112,2 | 114 ± 65,5 | | 276,1 ± 16,7 | 236 ± 21 | |
| 209798_at | | nuclear protein, ataxia-telangiectasia locus | NPAT | -2,41 | | 199 ± 11,2 | 89,2 ± 33,8 | | 215,5 ± 23,9 | 67,3 ± 2 | |
| 225768_at | | nuclear receptor subfamily 1, group D, member 2 | NR1D2 | -4,09 | | 422,1 ± 50,5 | 120,7 ± 33,4 | | 241,6 ± 23,1 | 200,6 ± 19,3 | |
| 216321_s_at | | nuclear receptor subfamily 3, group C, member 1 (glucocorticoid receptor) | NR3C1 | -2,32 | | 1080,2 ± 165,7 | 478,6 ± 215,7 | | 946,1 ± 112,6 | 655,2 ± 31,2 | |
| 224985_at | | neuroblastoma RAS viral (v-ras) oncogene homolog | NRAS | -2,24 | | 743,8 ± 62,4 | 375,3 ± 166,4 | | 583,8 ± 49,6 | 390,9 ± 29,5 | |
| 202599_s_at | | nuclear receptor interacting protein 1 | NRIP1 | -2,68 | | 830,2 ± 169,4 | 376,9 ± 176,9 | | 603,9 ± 15,4 | 186,4 ± 18 | |
| 224830_at | | nudix (nucleoside diphosphate linked moiety X)-type motif 21 | NUDT21 | -3,15 | | 774,5 ± 164,9 | 279,9 ± 150,1 | | 720,3 ± 52 | 283,2 ± 30,5 | |
| 226810_at | | opioid growth factor receptor-like 1 | OGFRL1 | -3,38 | | 490,8 ± 6,8 | 174,3 ± 125,7 | | 512,3 ± 102,8 | 133,6 ± 12,5 | |
| 212585_at | | oxysterol binding protein-like 8 | OSBPL8 | -2,99 | | 998 ± 133,7 | 379,1 ± 280 | | 1549,9 ± 176,4 | 215,5 ± 30,5 | |
| 225563_at | | PAN3 poly(A) specific ribonuclease subunit homolog (S. cerevisiae) | PAN3 | -2,72 | | 481 ± 31,7 | 195,7 ± 106 | | 511,2 ± 41,3 | 300,1 ± 17,7 | |
| 223238_s_at | | polybromo 1 | PBRM1 | -2,21 | | 477,8 ± 17,5 | 273,3 ± 73,7 | | 499,3 ± 44 | 275,6 ± 41,9 | |
| 1554868_s_at | | PEST proteolytic signal containing nuclear protein | PCNP | -2,48 | | 1146,2 ± 83,1 | 510,3 ± 271 | | 871 ± 59,8 | 625,9 ± 35 | |
| 244774_at | | phosphatase and actin regulator 2 | PHACTR2 | -2,74 | | 147,3 ± 26,4 | 50,9 ± 18,6 | | 140,9 ± 20,8 | 51,7 ± 4,5 | |
| 226508_at | | polyhomeotic homolog 3 (Drosophila) | PHC3 | -2,28 | | 569,1 ± 74,6 | 272,9 ± 93,9 | | 632,5 ± 50,3 | 230,9 ± 33,7 | |
| 217954_s_at | | PHD finger protein 3 | PHF3 | -2,70 | | 597,3 ± 69,4 | 232,6 ± 63,9 | | 665,4 ± 38,2 | 123,3 ± 19,3 | |
| 213074_at | | pleckstrin homology domain interacting protein | PHIP | -3,20 | | 353,4 ± 51,7 | 185,3 ± 124,1 | | 532,9 ± 53,9 | 104,9 ± 22 | |
| 227639_at | | phosphatidylinositol glycan anchor biosynthesis, class K | PIGK | -3,79 | | 718 ± 16,2 | 289,6 ± 236,1 | | 705,1 ± 33,3 | 189,4 ± 17,7 | |
| 220952_s_at | | pleckstrin homology domain containing, family A member 5 | PLEKHA5 | -5,44 | | 857,6 ± 229,4 | 157,8 ± 97,8 | | 1338,3 ± 216,6 | 388,9 ± 42,1 | |
| 201939_at | | polo-like kinase 2 (Drosophila) | PLK2 | -7,13 | | 468,4 ± 262,2 | 62,3 ± 46,9 | | 482,2 ± 6,8 | 132,3 ± 26,3 | |
| 213518_at | | protein kinase C, iota | PRKCI | -3,62 | | 459,1 ± 68,5 | 147,4 ± 94,3 | | 395,6 ± 24,4 | 213,6 ± 20,7 | |
| 218236_s_at | | protein kinase D3 | PRKD3 | -2,56 | | 585,1 ± 24,8 | 277,9 ± 162,4 | | 602,8 ± 61,6 | 230,3 ± 24,4 | |
| 218053_at | | PRP40 pre-mRNA processing factor 40 homolog A (S. cerevisiae) | PRPF40A | -2,26 | | 941,9 ± 39,5 | 509,1 ± 266,7 | | 1168 ± 148,2 | 338,7 ± 31,2 | |
| 205961_s_at | | PC4 and SFRS1 interacting protein 1 | PSIP1 | -4,42 | | 370,1 ± 44,8 | 88,2 ± 23,6 | | 308,3 ± 23 | 127,3 ± 18,7 | |
| 225363_at | | phosphatase and tensin homolog | PTEN | -2,23 | | 1692,3 ± 271,6 | 769,6 ± 320,5 | | 1489,5 ± 49,3 | 1023,6 ± 172,1 | |
| 204748_at | | prostaglandin-endoperoxide synthase 2 (prostaglandin G/H synthase and cyclooxygenase) | PTGS2 | -3,40 | | 150,8 ± 34,9 | 55,7 ± 35,3 | | 28,5 ± 1,5 | 32,3 ± 13,5 | |
| 211737_x_at | | pleiotrophin | PTN | -17,55 | | 1032,6 ± 94,3 | 159,5 ± 233,4 | | 1875,3 ± 67 | 239,2 ± 6,9 | |
| 204201_s_at | | protein tyrosine phosphatase, non-receptor type 13 (APO-1/CD95 (Fas)-associated phosphatase) | PTPN13 | -2,33 | | 444,6 ± 7,8 | 221 ± 96,8 | | 244,6 ± 51,1 | 206,2 ± 10,8 | |
| 225120_at | | purine-rich element binding protein B | PURB | -2,13 | | 561,1 ± 70,7 | 275,6 ± 45,8 | | 434,9 ± 4,5 | 363,1 ± 23,6 | |
| 226265_at | | glutamine and serine rich 1 | QSER1 | -2,68 | | 694,8 ± 66,7 | 259,2 ± 119,1 | | 686,5 ± 60,6 | 201,1 ± 32,3 | |
| 213405_at | | RAB22A, member RAS oncogene family | RAB22A | -2,52 | | 357,2 ± 40,4 | 152,9 ± 59,5 | | 371,4 ± 29,5 | 223,2 ± 44,9 | |
| 226633_at | | RAB8B, member RAS oncogene family | RAB8B | -2,28 | | 627,8 ± 90 | 315,4 ± 95,6 | | 943 ± 79,5 | 226,7 ± 55,5 | |
| 227224_at | | Ral GEF with PH domain and SH3 binding motif 2 | RALGPS2 | -4,16 | | 732,1 ± 123,9 | 180,9 ± 168,3 | | 737,7 ± 22,8 | 280,3 ± 24,9 | |
| 227036_at | | RAS protein activator like 2 | RASAL2 | -5,66 | | 314,7 ± 10,6 | 101,8 ± 109,6 | | 257,2 ± 15 | 177,1 ± 9,4 | |
| 225946_at | | Ras association (RalGDS/AF-6) domain family (N-terminal) member 8 | RASSF8 | -2,66 | | 485,6 ± 68,7 | 177,9 ± 64,6 | | 391,7 ± 55,4 | 183,4 ± 29,9 | |
| 203132_at | | retinoblastoma 1 | RB1 | -2,39 | | 392,6 ± 23,4 | 166,8 ± 71,7 | | 307,1 ± 58,2 | 130,4 ± 25,7 | |
| 203344_s_at | | retinoblastoma binding protein 8 | RBBP8 | -2,41 | | 435,8 ± 24,6 | 194,1 ± 106,1 | | 403,1 ± 30,7 | 142,8 ± 14,2 | |
| 225310_at | | RNA binding motif protein, X-linked | RBMX | -2,81 | | 774,7 ± 117,9 | 334,3 ± 254,5 | | 886,2 ± 24,8 | 445,8 ± 4,8 | |
| 222605_at | | REST corepressor 3 | RCOR3 | -2,21 | | 423,4 ± 92 | 189,8 ± 86,3 | | 578,6 ± 31,1 | 296 ± 46,2 | |
| 222630_at | | regulatory factor X, 7 | RFX7 | -2,18 | | 321,7 ± 39,6 | 147,1 ± 33,3 | | 289,9 ± 31,5 | 131,6 ± 30,4 | |
| 226312_at | | RPTOR independent companion of MTOR, complex 2 | RICTOR | -2,94 | | 290,9 ± 23 | 115,2 ± 51,4 | | 321,8 ± 37,9 | 37,1 ± 7,2 | |
| 212724_at | | Rho family GTPase 3 | RND3 | -4,13 | | 4145,1 ± 193,9 | 1137,7 ± 557,5 | | 5566,6 ± 179,9 | 1400,5 ± 102 | |
| 218738_s_at | | ring finger protein 138 | RNF138 | -2,14 | | 284,6 ± 40,5 | 141,4 ± 28,2 | | 245,1 ± 25,1 | 135,9 ± 20,2 | |
| 202683_s_at | | RNA (guanine-7-) methyltransferase | RNMT | -2,68 | | 292,8 ± 62,9 | 123,5 ± 46,9 | | 263,7 ± 29 | 168,1 ± 14,6 | |
| 228963_at | | Round spermatid basic protein 1-like | RSBN1L | -3,13 | | 217,4 ± 11,3 | 62 ± 34,4 | | 272,2 ± 29,2 | 106,1 ± 35,9 | |
| 213939_s_at | | RUN and FYVE domain containing 3 | RUFY3 | -4,00 | | 365,9 ± 110,7 | 96,1 ± 78,2 | | 476,5 ± 9,1 | 133,2 ± 17,4 | |
| 228531_at | | sterile alpha motif domain containing 9 | SAMD9 | -4,67 | | 176,2 ± 32,1 | 39 ± 33,9 | | 216,1 ± 28,9 | 40,2 ± 8,4 | |
| 228174_at | | suppressor of cancer cell invasion | SCAI | -7,52 | | 104 ± 24,3 | 17,4 ± 17,1 | | 116,9 ± 4,4 | 33,9 ± 6 | |
| 212416_at | | secretory carrier membrane protein 1 | SCAMP1 | -3,10 | | 1315,7 ± 108,4 | 515,8 ± 365,7 | | 1177 ± 92,6 | 519,5 ± 60,5 | |
| 212493_s_at | | SET domain containing 2 | SETD2 | -2,42 | | 256,9 ± 21,5 | 107,8 ± 25,8 | | 236,5 ± 25,3 | 114,5 ± 29 | |
| 226412_at | | splicing factor, arginine/serine-rich 18 | SFRS18 | -2,28 | | 274,8 ± 35,2 | 128,5 ± 32,2 | | 307 ± 14 | 162,1 ± 11,7 | |
| 226601_at | | solute carrier family 30 (zinc transporter), member 7 | SLC30A7 | -3,30 | | 474,3 ± 61 | 135,2 ± 75,4 | | 420,8 ± 42,9 | 183,9 ± 38,5 | |
| 225295_at | | solute carrier family 39 (zinc transporter), member 10 | SLC39A10 | -6,75 | | 479,4 ± 92,3 | 148,5 ± 146,5 | | 302,5 ± 17,7 | 142,4 ± 34,1 | |
| 209884_s_at | | solute carrier family 4, sodium bicarbonate cotransporter, member 7 | SLC4A7 | -4,06 | | 507,2 ± 132,6 | 117 ± 91,1 | | 1280,7 ± 111,3 | 130 ± 21,4 | |
| 213164_at | | solute carrier family 5 (sodium/myo-inositol cotransporter), member 3 | SLC5A3 | -2,81 | | 1788,8 ± 341,8 | 713,2 ± 512,4 | | 1005 ± 22,6 | 691,6 ± 160,3 | |
| 238430_x_at | | schlafen family member 5 | SLFN5 | -2,24 | | 646,2 ± 141,5 | 314,4 ± 177,2 | | 1186,5 ± 76,4 | 431,3 ± 77,3 | |
| 225219_at | | SMAD family member 5 | SMAD5 | -2,60 | | 775 ± 109,3 | 336 ± 151,7 | | 807,8 ± 54,2 | 389 ± 75,9 | |
| 211988_at | | SWI/SNF related, matrix associated, actin dependent regulator of chromatin, subfamily e, member 1 | SMARCE1 | -3,76 | | 2064,3 ± 200,1 | 764,1 ± 639,1 | | 1755,5 ± 122,4 | 1296,6 ± 56,8 | |
| 212927_at | | structural maintenance of chromosomes 5 | SMC5 | -5,00 | | 168,3 ± 7,1 | 46,2 ± 38,6 | | 177,1 ± 49,4 | 33 ± 2,5 | |
| 227542_at | | suppressor of cytokine signaling 6 | SOCS6 | -2,72 | | 255,3 ± 36,6 | 102,7 ± 14,4 | | 248,5 ± 38,4 | 79,1 ± 19,3 | |
| 204914_s_at | | SRY (sex determining region Y)-box 11 | SOX11 | -18,24 | | 87,6 ± 25,3 | 7,2 ± 8,6 | | 31,6 ± 4 | 3,9 ± 2,2 | |
| 209748_at | | spastin | SPAST | -2,18 | | 228,7 ± 37,9 | 86,6 ± 13,1 | | 231,6 ± 46,5 | 99,2 ± 24,6 | |
| 235074_at | | sprouty-related, EVH1 domain containing 1 | SPRED1 | -3,91 | | 223,1 ± 64 | 48,2 ± 18,1 | | 92,9 ± 12,7 | 82,2 ± 6 | |
| 203017_s_at | | synovial sarcoma, X breakpoint 2 interacting protein | SSX2IP | -5,57 | | 165,7 ± 47,3 | 30,2 ± 30,6 | | 78,5 ± 3,7 | 38,8 ± 3,4 | |
| 202293_at | | stromal antigen 1 | STAG1 | -2,46 | | 227 ± 48,3 | 111,4 ± 39,6 | | 203 ± 11,4 | 130,9 ± 9,6 | |
| 209022_at | | stromal antigen 2 | STAG2 | -3,03 | | 1021,6 ± 114,1 | 388,3 ± 239,7 | | 1197,7 ± 94,8 | 201,5 ± 13,2 | |
| 226525_at | | serine/threonine kinase 17b | STK17B | -3,03 | | 1129,1 ± 504,3 | 369,5 ± 139,1 | | 2211,8 ± 242,2 | 728,7 ± 34,6 | |
| 217815_at | | suppressor of Ty 16 homolog (S. cerevisiae) | SUPT16H | -2,06 | | 713,9 ± 69,9 | 351,1 ± 143,9 | | 728,1 ± 38,3 | 451,3 ± 49 | |
| 218520_at | | TANK-binding kinase 1 | TBK1 | -2,44 | | 448,5 ± 50,2 | 220,9 ± 103,2 | | 457,5 ± 37,6 | 149,3 ± 15,5 | |
| 227624_at | | tet oncogene family member 2 | TET2 | -4,39 | | 84,5 ± 15,2 | 31,3 ± 26,9 | | 67,1 ± 11,5 | 20,3 ± 3,6 | |
| 213258_at | | tissue factor pathway inhibitor (lipoprotein-associated coagulation inhibitor) | TFPI | -2,92 | | 3214,2 ± 636,2 | 1153 ± 619,1 | | 2932 ± 188,4 | 1204,5 ± 134,4 | |
| 202606_s_at | | tousled-like kinase 1 | TLK1 | -2,09 | | 527,4 ± 60,5 | 282,3 ± 64,1 | | 629,8 ± 61,1 | 284 ± 26,4 | |
| 225411_at | | transmembrane protein 87B | TMEM87B | -4,06 | | 364,3 ± 29,7 | 106,7 ± 90,1 | | 244,3 ± 19,4 | 184,5 ± 21,6 | |
| 223077_at | | tropomodulin 3 (ubiquitous) | TMOD3 | -3,17 | | 572,6 ± 73,3 | 180,7 ± 99,1 | | 412 ± 72,4 | 286,8 ± 39,7 | |
| 224944_at | | thymopoietin | TMPO | -3,05 | | 320,4 ± 99 | 105,8 ± 44,8 | | 250,7 ± 22,8 | 149,4 ± 9,6 | |
| 205347_s_at | | thymosin beta 15a | TMSB15A | -5,70 | | 217 ± 112,3 | 26,3 ± 13,4 | | 81,8 ± 5,7 | 59,4 ± 1,4 | |
| 206025_s_at | | tumor necrosis factor, alpha-induced protein 6 | TNFAIP6 | -4,89 | | 1269 ± 379,8 | 312,5 ± 152,1 | | 628 ± 58,6 | 422 ± 19,5 | |
| 218228_s_at | | tankyrase, TRF1-interacting ankyrin-related ADP-ribose polymerase 2 | TNKS2 | -2,05 | | 615,6 ± 91,5 | 360,8 ± 192,1 | | 520 ± 104,8 | 354,7 ± 60,2 | |
| 224704_at | | trinucleotide repeat containing 6A | TNRC6A | -2,70 | | 464,7 ± 58,1 | 160,3 ± 111,2 | | 480,5 ± 96,9 | 242,4 ± 19,7 | |
| 203375_s_at | | tripeptidyl peptidase II | TPP2 | -3,20 | | 396,3 ± 24,7 | 141,5 ± 102,8 | | 340,2 ± 45,7 | 157,9 ± 23,7 | |
| 215945_s_at | | tripartite motif-containing 2 | TRIM2 | -2,74 | | 140,9 ± 79 | 47 ± 11,8 | | 126 ± 33,5 | 41,5 ± 12,4 | |
| 212435_at | | tripartite motif-containing 33 | TRIM33 | -2,56 | | 199,3 ± 37 | 80,3 ± 26,1 | | 229 ± 34,1 | 40 ± 9,9 | |
| 218502_s_at | | trichorhinophalangeal syndrome I | TRPS1 | -3,79 | | 479,2 ± 195,8 | 162 ± 95,7 | | 305,3 ± 27,5 | 198,6 ± 42,5 | |
| 201534_s_at | | ubiquitin-like 3 | UBL3 | -2,19 | | 791,3 ± 113,2 | 329,8 ± 181,1 | | 805 ± 48,8 | 431,5 ± 41,3 | |
| 213118_at | | UHRF1 binding protein 1-like | UHRF1BP1L | -3,73 | | 309,8 ± 55,4 | 109,8 ± 86,6 | | 222,9 ± 41,8 | 116,3 ± 14,1 | |
| 202413_s_at | | ubiquitin specific peptidase 1 | USP1 | -2,24 | | 574,6 ± 45,5 | 286,4 ± 148,6 | | 544,3 ± 58,7 | 198,2 ± 14,4 | |
| 212066_s_at | | ubiquitin specific peptidase 34 | USP34 | -2,96 | | 755,7 ± 27,3 | 322 ± 189,8 | | 711,1 ± 36,2 | 234,5 ± 23,5 | |
| 228492_at | | ubiquitin specific peptidase 9, Y-linked | USP9Y | -19,40 | | 176 ± 36,8 | 19,7 ± 22,7 | | 176,6 ± 16,2 | 41,7 ± 8,1 | |
| 233632_s_at | | 5'-3' exoribonuclease 1 | XRN1 | -3,73 | | 368,1 ± 71,1 | 120,9 ± 88,3 | | 420,4 ± 72,4 | 83,1 ± 20,6 | |
| 202932_at | | v-yes-1 Yamaguchi sarcoma viral oncogene homolog 1 | YES1 | -2,92 | | 516,5 ± 65,4 | 192,7 ± 113,8 | | 397,4 ± 31 | 107,7 ± 11,3 | |
| 225662_at | | sterile alpha motif and leucine zipper containing kinase AZK | ZAK | -2,83 | | 1194 ± 23,5 | 485,9 ± 239,4 | | 1026,6 ± 55,4 | 406,2 ± 55,4 | |
| 218263_s_at | | zinc finger, BED-type containing 5 | ZBED5 | -2,99 | | 694,7 ± 40,6 | 259,8 ± 136,9 | | 690,6 ± 79,2 | 371,2 ± 28 | |
| 226255_at | | zinc finger and BTB domain containing 33 | ZBTB33 | -2,16 | | 439,2 ± 57,6 | 233,7 ± 105,2 | | 525,4 ± 30,9 | 163,8 ± 33,8 | |
| 222731_at | | zinc finger, DHHC-type containing 2 | ZDHHC2 | -4,03 | | 571,8 ± 77,6 | 187,1 ± 173,1 | | 402,7 ± 40,8 | 264,7 ± 4 | |
| 202051_s_at | | zinc finger, MYM-type 4 | ZMYM4 | -3,05 | | 463,6 ± 35,1 | 200,7 ± 152,6 | | 458,4 ± 46 | 204,8 ± 32,5 | |
| 228785_at | | Zinc finger protein 281 | ZNF281 | -2,02 | | 514 ± 25,2 | 254,9 ± 130,4 | | 579,7 ± 40,3 | 265,4 ± 16,2 | |
| 227077_at | | zinc finger protein 286A /// zinc finger protein 286B | ZNF286A /// ZNF286B | -2,24 | | 386,5 ± 96,5 | 168,2 ± 35 | | 419 ± 45,9 | 237,8 ± 20,8 | |
| 231864_at | | zinc finger protein 33A | ZNF33A | -7,46 | | 130,5 ± 66,6 | 17,8 ± 20,4 | | 175,4 ± 16,8 | 38 ± 7,6 | |
| 226327_at | | zinc finger protein 507 | ZNF507 | -2,52 | | 241,5 ± 12 | 107,2 ± 60,5 | | 239,4 ± 25,3 | 88,9 ± 9,1 | |
| 204453_at | | zinc finger protein 84 | ZNF84 | -3,22 | | 221 ± 56,3 | 66,9 ± 39,6 | | 330,9 ± 25,6 | 47,1 ± 24,2 | |
| 212893_at | | zinc finger, ZZ-type containing 3 | ZZZ3 | -2,32 | | 427,7 ± 67,9 | 190,2 ± 53,3 | | 479,2 ± 49,6 | 199,6 ± 19,7 | |
