# Supplementary material for: Reverse Differentiation as a Gene Filtering Tool in Genome Expression Profiling of Adipogenesis for Fat Marker Gene Selection and Their Analysis
Source: PLoS One. 2013 Jul 26;8(7):e69754. doi: 10.1371/journal.pone.0069754 (PMC3724870; doi:10.1371/journal.pone.0069754)
Supplement: Table S2 — Transcription factor binding sites (TFBS) included in each cluster. The numbers of transcription factor binding sites (TFBS) are given in this table, and are organized according to ascending alphabetical order. The number in brackets represents the number of transcription factor binding sites (TFBS), and these TFBS are specific to each cluster gene as given in the Suppl. Table S1. For more detail of gene titles and expression values of the respective cluster genes, see Suppl. Table S1. (DOCX) [file pone.0069754.s005.docx]

# SUPPLEMENTARY TABLE

## Table S2. Transcription factor binding sites (TFBS) included in each cluster.

## The numbers of transcription factor binding sites (TFBS) are given in this table, and are organized according to ascending alphabetical order. The number in brackets represents the number of transcription factor binding sites (TFBS), and these TFBS are specific to each cluster gene as given in the Suppl. Table S1. For more detail of gene titles and expression values of the respective cluster genes, see Suppl. Table S1.

| **Transcrption Factors Binding Sites (TFBS) in each cluster** | | | | | | | | |
| --- | --- | --- | --- | --- | --- | --- | --- | --- |
| **Cluster 1** | **Cluster 2** | | | **Cluster 3** | | | **Cluster 4** |  |
| *AHRARNT (149)* | *AHR (65)* | *GATA (107)* | *NKX25 (121)* | *AML1 (215)* | *GATA (147)* | *NGFIC (83)* | *AML1 (167)* | *IRF2 (106)* |
| *AP1 (174)* | *AML1 (158)* | *GATA1 (160)* | *NKX61 (93)* | *AP1 (187)* | *GATA1 (226)* | *NKX22 (167)* | *AP1 (132)* | *IRF7 (121)* |
| *AP2REP (128)* | *AP1 (135)* | *GATA3 (51)* | *NMYC (67)* | *AP1FJ (95)* | *GATA2 (98)* | *NKX25 (191)* | *AP1FJ (63)* | *ISRE (108)* |
| *ARNT (136)* | *AP1FJ (65)* | *GATA6 (38)* | *NRSF (102)* | *AP2REP (119)* | *GATA3 (82)* | *NKX3A (174)* | *AREB6 (173)* | *LHX3 (117)* |
| *ARP1 (139)* | *AP2REP (99)* | *GCNF (119)* | *OCT (99)* | *AP4 (169)* | *GATA6 (73)* | *NKX61 (152)* | *BACH1 (106)* | *MEF2 (190)* |
| *BACH1 (144)* | *AP4 (127)* | *GFI1 (99)* | *OCT1 (171)* | *AREB6 (226)* | *GCNF (162)* | *OCT (158)* | *BRACH (125)* | *MEIS1 (95)* |
| *BACH2 (134)* | *ARNT (90)* | *GR (105)* | *P300 (78)* | *ARNT (132)* | *GFI1 (146)* | *OCT1 (235)* | *BRN2 (133)* | *MRF2 (129)* |
| *CEBPA (84)* | *ATF6 (104)* | *GRE (99)* | *P53 (132)* | *ATF (83)* | *GR (157)* | *P300 (107)* | *CART1 (154)* | *MSX1 (108)* |
| *CEBPB (177)* | *BACH1 (113)* | *HFH1 (102)* | *PAX2 (114)* | *ATF6 (119)* | *GRE (143)* | *P53 (183)* | *CDC5 (152)* | *MYB (89)* |
| *COUP (118)* | *BACH2 (104)* | *HFH3 (103)* | *PAX3 (88)* | *BACH1 (174)* | *HFH1 (161)* | *PAX2 (158)* | *CDP (173)* | *NCX (96)* |
| *E47 (171)* | *BRACH (114)* | *HLF (85)* | *PAX4 (150)* | *BACH2 (146)* | *HFH3 (143)* | *PAX3 (131)* | *CDPCR1 (96)* | *NFAT (125)* |
| *EGR3 (52)* | *BRN2 (99)* | *HMX1 (92)* | *PAX5 (108)* | *BRACH (161)* | *HLF (146)* | *PAX4 (206)* | *CEBP (169)* | *NFE2 (77)* |
| *FREAC2 (94)* | *CART1 (90)* | *HNF1 (113)* | *PBX1 (118)* | *BRN2 (157)* | *HMX1 (126)* | *PAX6 (171)* | *CEBPA (80)* | *NFY (101)* |
| *GCNF (162)* | *CDC5 (91)* | *HNF3B (86)* | *POU3F2 (117)* | *CART1 (158)* | *HNF1 (175)* | *PBX1 (184)* | *CEBPB (133)* | *NKX22 (142)* |
| *HAND1E47 (134)* | *CDP (127)* | *HOX13 (91)* | *POU6F1 (81)* | *CDC5 (167)* | *HNF3B (151)* | *POU3F2 (204)* | *CHOP (98)* | *NKX25 (165)* |
| *HNF4 (146)* | *CEBP (152)* | *HOXA3 (88)* | *PPARA (108)* | *CDP (202)* | *HNF4 (133)* | *POU6F1 (154)* | *CHX10 (112)* | *NKX3A (156)* |
| *IK3 (125)* | *CEBPA (67)* | *HSF1 (71)* | *PPARG (155)* | *CDPCR1 (135)* | *HOX13 (121)* | *PPARA (141)* | *CREBP1 (130)* | *NKX61 (137)* |
| *MYB (131)* | *CHOP (102)* | *HSF2 (89)* | *RFX1 (118)* | *CDPCR3 (157)* | *HOXA3 (130)* | *PPARG (216)* | *E2F (118)* | *OCT (139)* |
| *MYCMAX (184)* | *CHX10 (89)* | *IK3 (89)* | *RORA1 (85)* | *CEBP (216)* | *HSF1 (100)* | *RFX1 (179)* | *E4BP4 (141)* | *OCT1 (200)* |
| *NFE2 (105)* | *CMYB (90)* | *IRF1 (60)* | *RORA2 (94)* | *CEBPA (105)* | *HSF2 (138)* | *RORA1 (145)* | *EN1 (132)* | *PAX4 (170)* |
| *NFKAPPAB (102)* | *COMP1 (94)* | *IRF7 (104)* | *RP58 (115)* | *CEBPB (186)* | *HTF (148)* | *RORA2 (153)* | *EVI1 (197)* | *PAX6 (133)* |
| *NFKB (144)* | *COUP (87)* | *ISRE (100)* | *RSRFC4 (105)* | *CETS1P54 (71)* | *IK1 (102)* | *RP58 (175)* | *FAC1 (112)* | *PBX1 (154)* |
| *NFY (142)* | *CP2 (78)* | *LHX3 (82)* | *S8 (103)* | *CHOP (153)* | *IK2 (86)* | *RREB1 (99)* | *FOXD3 (120)* | *POU3F2 (176)* |
| *NMYC (91)* | *CREB (84)* | *LYF1 (78)* | *SEF1 (106)* | *CHX10 (146)* | *IK3 (143)* | *RSRFC4 (166)* | *FOXJ2 (177)* | *POU6F1 (145)* |
| *NRSF (157)* | *E2F (113)* | *MAX (37)* | *SOX5 (105)* | *CMYB (124)* | *IRF1 (114)* | *S8 (156)* | *FOXO1 (123)* | *RFX1 (119)* |
| *PPARA (160)* | *E47 (124)* | *MEF2 (145)* | *SOX9 (100)* | *COMP1 (137)* | *IRF2 (149)* | *SEF1 (136)* | *FOXO3 (89)* | *RORA1 (106)* |
| *PPARG (234)* | *E4BP4 (90)* | *MEIS1 (95)* | *SREBP1 (114)* | *COUP (130)* | *IRF7 (149)* | *SOX5 (163)* | *FOXO4 (148)* | *RORA2 (124)* |
| *ROAZ (137)* | *EGR2 (21)* | *MIF1 (80)* | *SRF (133)* | *CP2 (106)* | *ISRE (161)* | *SOX9 (148)* | *FREAC2 (102)* | *RP58 (115)* |
| *RSRFC4 (137)* | *ELK1 (97)* | *MRF2 (97)* | *SRY (92)* | *CREBP1 (143)* | *LHX3 (135)* | *SPZ1 (92)* | *FREAC3 (115)* | *RSRFC4 (129)* |
| *SEF1 (149)* | *EN1 (116)* | *MSX1 (90)* | *STAT (81)* | *CREL (49)* | *LUN1 (139)* | *SREBP1 (169)* | *FREAC4 (129)* | *S8 (121)* |
| *TCF11 (150)* | *EVI1 (160)* | *MYOD (111)* | *STAT1 (88)* | *E2F (162)* | *LYF1 (121)* | *SRF (202)* | *FREAC7 (146)* | *SEF1 (100)* |
| *USF (174)* | *FAC1 (96)* | *MZF1 (95)* | *STAT3 (110)* | *E47 (168)* | *MAX (49)* | *SRY (150)* | *GATA (123)* | *SOX5 (151)* |
|  | *FOXD3 (76)* | *NCX (96)* | *STAT5A (117)* | *E4BP4 (156)* | *MEF2 (216)* | *STAT (115)* | *GATA1 (178)* | *SOX9 (116)* |
|  | *FOXJ2 (141)* | *NF1 (76)* | *STAT5B (72)* | *EN1 (159)* | *MEIS1 (132)* | *STAT1 (133)* | *GATA6 (50)* | *SRF (146)* |
|  | *FOXO1 (90)* | *NFAT (89)* | *TATA (112)* | *ER (138)* | *MIF1 (108)* | *STAT3 (140)* | *GCNF (120)* | *SRY (136)* |
|  | *FOXO3 (61)* | *NFE2 (78)* | *TBP (74)* | *EVI1 (240)* | *MRF2 (164)* | *STAT5A (175)* | *GFI1 (101)* | *STAT (78)* |
|  | *FOXO4 (111)* | *NFKB (115)* | *TCF11 (104)* | *FAC1 (154)* | *MSX1 (128)* | *STAT5B (121)* | *GRE (96)* | *STAT1 (80)* |
|  | *FREAC2 (81)* | *NFY (92)* | *TGIF (96)* | *FOXD3 (133)* | *MYB (124)* | *TATA (168)* | *HAND1E47 (98)* | *STAT3 (101)* |
|  | *FREAC3 (98)* | *NGFIC (64)* | *TST1 (86)* | *FOXJ2 (200)* | *MYOD (164)* | *TBP (133)* | *HFH1 (142)* | *STAT5A (116)* |
|  | *FREAC4 (101)* | *NKX22 (94)* | *USF (111)* | *FOXO1 (159)* | *MZF1 (143)* | *TCF11 (150)* | *HFH3 (133)* | *STAT5B (91)* |
|  | *FREAC7 (102)* |  | *ZIC3 (80)* | *FOXO3 (109)* | *NCX (134)* | *TGIF (135)* | *HLF (127)* | *TATA (152)* |
|  |  |  |  | *FOXO4 (185)* | *NF1 (111)* | *TST1 (149)* | *HNF1 (152)* | *TBP (105)* |
|  |  |  |  | *FREAC2 (124)* | *NFAT (152)* | *USF (157)* | *HNF3B (124)* | *TCF11 (107)* |
|  |  |  |  | *FREAC3 (153)* | *NFE2 (112)* | *XBP1 (130)* | *HOXA3 (95)* | *TGIF (92)* |
|  |  |  |  | *FREAC4 (168)* | *NFKB (151)* | *YY1 (192)* | *HSF1 (73)* | *TST1 (111)* |
|  |  |  |  | *FREAC7 (162)* | *NFY (131)* | *ZIC2 (64)* | *HSF2 (94)* | *YY1 (140)* |
|  |  |  |  |  |  | *ZIC3 (107)* | *IK3 (97)* |  |
